# Construction of Phenanthridinone Skeletons through Palladium-Catalyzed Annulation

**DOI:** 10.1021/acs.joc.3c01429

**Published:** 2023-08-23

**Authors:** Xin Geng, Heng He, Andrey Shatskiy, Elena V. Stepanova, Gregory R. Alvey, Jian-Quan Liu, Markus D. Kärkäs, Xiang-Shan Wang

**Affiliations:** † School of Chemistry and Materials Science, Jiangsu Key Laboratory of Green Synthesis for Functional Materials Jiangsu Normal University, Xuzhou, Jiangsu 221116, China; ‡ Department of Chemistry, KTH Royal Institute of Technology, SE-100 44 Stockholm, Sweden; ∥ Tomsk Polytechnic University, Lenin Avenue 30, 634050 Tomsk, Russia

## Abstract

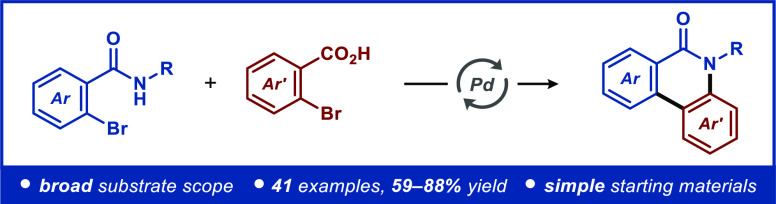

Herein, a straightforward synthetic approach for the construction of phenanthridin-6(5*H*)-one skeletons is disclosed. The developed protocol relies on palladium catalysis, providing controlled access to a range of functionalized phenanthridin-6(5*H*)-ones in 59–88% yields. Furthermore, plausible reaction pathways are proposed based on mechanistic experiments.

## Introduction

**Figure 1:**
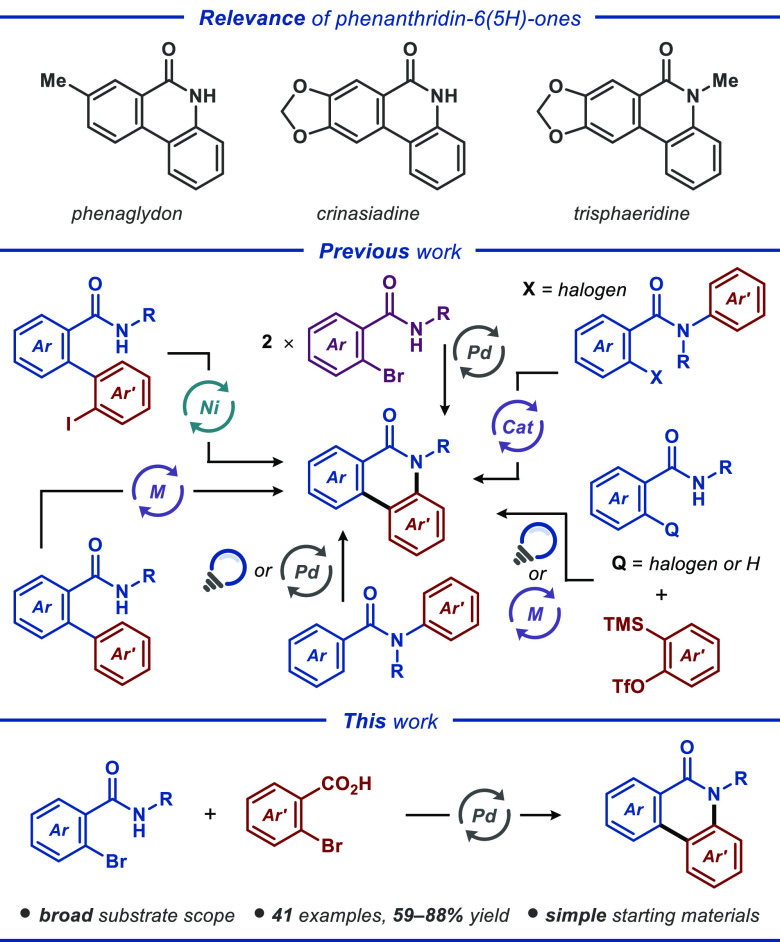
Relevance and synthetic approaches to phenanthridin-6(5*H*)-one derivatives.

Phenanthridin-6(5*H*)-one represents a class of tricyclic *N*-heterocycles that is frequently encountered in alkaloids, such as phenaglydon, crinasiadine, and trisphaeridine (Figure 1, top). These compounds have been documented to possess biological and pharmaceutical activities, including antimycobacterial,^1^ antagonistic,^2^ antiproliferative,^3^ and antitubercular activities.^4^ Significant attention has been devoted to developing novel synthetic methods for the construction of phenanthridin-6(5*H*)-one derivatives (Figure 1, middle).^6^ The Yamada group demonstrated the synthesis of phenanthridin-6(5*H*)-ones through nickel-catalyzed amidation of aryl iodides.^7^ At the same time, Chaudhary and co-workers disclosed an organocatalytic protocol proceeding through direct C(sp^2^)–H bond arylation.^8^ Similarly, phenanthridin-6(5*H*)-one derivatives have also been accessed in high yields using the free radical initiator AIBN^9^ or microwave irradiation.^10^ Furthermore, phenanthridin-6(5*H*)-one derivatives have been efficiently assembled from 2-bromophenylbenzamides through a palladium-catalyzed process involving aryl–aryl coupling and deamidation.^11^ Various strategies have utilized the oxidative coupling of benzamides to construct phenanthridin-6(5*H*)-one scaffolds. These annulation approaches do not require *ortho*-halogenation and have been realized with transition-metal-catalyzed^12^ or photoinduced^13^ manifolds. In recent years, a direct *ortho*-C–H/N–H annulation was developed to yield phenanthridin-6(5*H*)-one derivatives from benzamide and the aryne precursor 2-(trimethylsilyl)phenyl trifluoromethanesulfonate using O_2_ or K_2_S_2_O_8_ as oxidizing agents.^14^

It has been demonstrated that 2-bromobenzoic acid can be easily converted to the corresponding aryne in the presence of a Pd catalyst.^15^ However, the generated aryne quickly undergoes a trimerization reaction to yield triphenylenes. In this context, we recently reported that 2-(2-bromophenyl)-1*H*-benzo[*d*]-imidazole derivatives can be harnessed as an effective coupling partner in combination with 2-bromobenzoic acids to give the corresponding *N*-fused (benzo)imidazophenanthridine scaffolds in high yields.^16^ In continuation of our previous studies directed to transition-metal-assisted synthesis of heterocycles,^17^ we envisaged that phenanthridin-6(5*H*)-one derivatives could be directly assembled from *N*-substituted 2-bromobenzamides **1** and 2-bromobenzoic acids **2** in the presence of a metal catalyst (Figure 1, bottom).

## Results and Discussion

We commenced our investigation by utilizing 2-bromo-*N*-methylbenzamide (**1a**) and 2-bromobenzoic acid (**2a**) as the model substrates, CuI as the catalyst precursor, and K_2_CO_3_ as the base in DMF at 100 °C. To our disappointment, the desired product **3a** was not detected under these reaction conditions (Table 1, entry 1). A similar outcome was observed with AgOTf as the metal catalyst (Table 1, entry 2). Gratifyingly, formation of the desired annulation product **3a** could be promoted by palladium-based catalysts, including Pd(OAc)_2_, PdCl_2_, Pd(PPh_3_)_2_Cl_2_, and Pd(PPh_3_)_4_ (Table 1, entries 3–6), with Pd(OAc)_2_ displaying the best reactivity and furnishing the desired product in 54% yield (Table 1, entry 3). Notably, the addition of auxiliary phosphine-based ligands, such as PPh_3_, Xantphos, P(4-MeOC_6_H_4_)_3_, and P(4-MeC_6_H_4_)_3_, promoted the desired reactivity (Table 1, entries 7–10) with PPh_3_ providing product **3a** in 70% yield (Table 1, entry 7). Apart from K_2_CO_3_, other common bases, such as Na_2_CO_3_, Cs_2_CO_3_, and ^*t*^BuOK, were evaluated and found less critical for the desired transformation (Table 1, entries 11–13). Carrying out the reaction under the optimized conditions for our previously disclosed protocol^16^ for the synthesis of *N*-fused (benzo)imidazophenanthridine scaffolds did not afford the desired annulation product **3a** (Table 1, entry 14). Instead, the trimerization product (triphenylene) was afforded under these reaction conditions. Next, the effect of the reaction temperature was examined (Table 1, entries 15–18) with 120 °C being the most suitable for the developed protocol. The use of polar aprotic solvents, such as DMF, DMSO, and DMA, was revealed to be beneficial (Table 1, entries 17, 19–20), while the nonpolar solvents xylene and toluene resulted in slightly diminished yields (Table 1, entries 21–22). Finally, a control experiment conducted in the absence of Pd(OAc)_2_ highlights the critical role of the palladium precursor in achieving effective coupling (Table 1, entry 23).

**Table 1:**
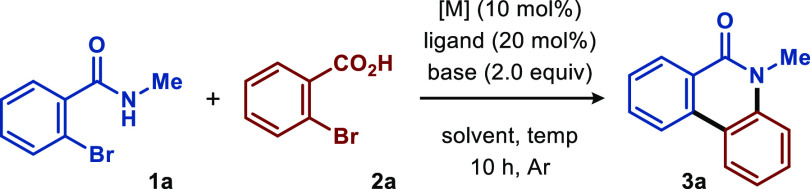
Optimization of Reaction Conditions^a^

entry	[M]	ligand	base	solvent	temp (°C)	yield (%)^b^
1	CuI	–	K_2_CO_3_	DMF	100	0
2	AgOTf	–	K_2_CO_3_	DMF	100	0
3	Pd(OAc)_2_	–	K_2_CO_3_	DMF	100	54
4	PdCl_2_	–	K_2_CO_3_	DMF	100	31
5	Pd(PPh_3_)_2_Cl_2_	–	K_2_CO_3_	DMF	100	40
6	Pd(PPh_3_)_4_	–	K_2_CO_3_	DMF	100	48
7	Pd(OAc)_2_	PPh_3_	K_2_CO_3_	DMF	100	70
8	Pd(OAc)_2_	Xantphos	K_2_CO_3_	DMF	100	68
9	Pd(OAc)_2_	P(4-MeOC_6_H_4_)_3_	K_2_CO_3_	DMF	100	64
10	Pd(OAc)_2_	P(4-MeC_6_H_4_)_3_	K_2_CO_3_	DMF	100	69
11	Pd(OAc)_2_	PPh_3_	Na_2_CO_3_	DMF	100	64
12	Pd(OAc)_2_	PPh_3_	Cs_2_CO_3_	DMF	100	72
13	Pd(OAc)_2_	PPh_3_	^*t*^BuOK	DMF	100	67
14^c^	Pd(OAc)_2_/CuI	PPh_3_	Cs_2_CO_3_	DMF	110	0
15	Pd(OAc)_2_	PPh_3_	Cs_2_CO_3_	DMF	80	57
16	Pd(OAc)_2_	PPh_3_	Cs_2_CO_3_	DMF	110	73
**17**	**Pd(OAc)_2_**	**PPh_3_**	**Cs_2_CO_3_**	**DMF**	**120**	**75**
18	Pd(OAc)_2_	PPh_3_	Cs_2_CO_3_	DMF	130	73
19	Pd(OAc)_2_	PPh_3_	Cs_2_CO_3_	DMSO	120	73
20	Pd(OAc)_2_	PPh_3_	Cs_2_CO_3_	DMA	120	72
21	Pd(OAc)_2_	PPh_3_	Cs_2_CO_3_	xylene	120	65
22	Pd(OAc)_2_	PPh_3_	Cs_2_CO_3_	toluene	120	70
23	–	PPh_3_	Cs_2_CO_3_	DMF	120	0

a Reaction conditions: Reactions were carried out with **1a** (107 mg, 0.50 mmol), **2a** (121 mg, 0.60 mmol), catalyst (10 mol %), ligand (20 mol %), and base (1.0 mmol) in solvent (5.0 mL) under argon for 10 h.

b Isolated yields of **3a** after purification by column chromatography.

c Reaction was carried out with **1a** (107 mg, 0.50 mmol), 2a (121 mg, 0.60 mmol), Pd(OAc)_2_ (5 mol %), CuI (10 mol %), PPh_3_ (20 mol %), and Cs_2_CO_3_ (0.5 mmol) in DMF (5.0 mL) under argon for 8 h.

After the optimal reaction conditions were identified, the scope and limitations of the developed protocol were evaluated. Initially, a series of *N*-substituted 2-bromobenzamides **1** were engaged in a reaction with 2-bromobenzoic acid **2a**. Aliphatic groups, such as methyl, ethyl, ^*n*^propyl, and ^*n*^butyl, all furnished the corresponding products **3b**–**3f** and **3h**–**3i** in moderate to high yields (61–75%). However, *N*-^*t*^butyl-2-bromobenzamide failed to produce the desired annulation product **3g**, presumably due to ample steric hindrance. The use of 2-bromobenzamides **1** bearing various *N*-substituted aromatic and heteroaromatic moieties demonstrated that various functional groups, such as halogens, ethers, nitrile, furan, and thiophene, were compatible with the developed protocol, furnishing products **3j**–**3t** in moderate to high yields (66–82%). The structure of product **3j** was confirmed by single-crystal X-ray analysis (CCDC 2210960).

**Scheme 1:**
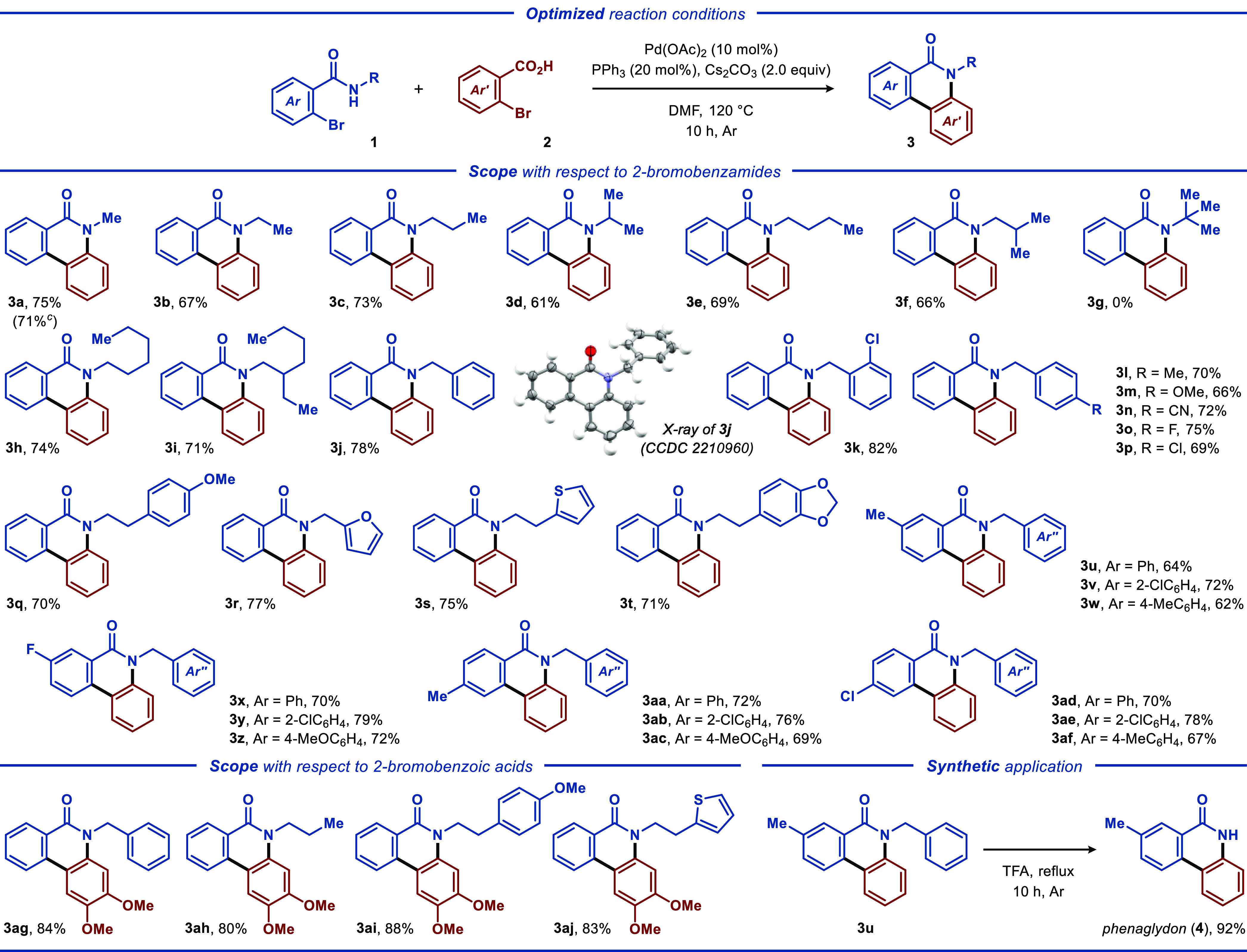
Reaction Scope and Synthetic Application^,^ Reaction conditions: Reactions were carried out with **1** (0.50 mmol), **2** (0.60 mmol), Pd(OAc)_2_ (12 mg, 0.05 mmol), PPh_3_ (26 mg, 0.10 mmol), and Cs_2_CO_3_ (326 mg, 1.0 mmol) in DMF (5.0 mL) under argon at 120 °C for 10 h. Isolated product yields are reported. Reaction carried out on a 1 mmol scale.

**Scheme 2:**
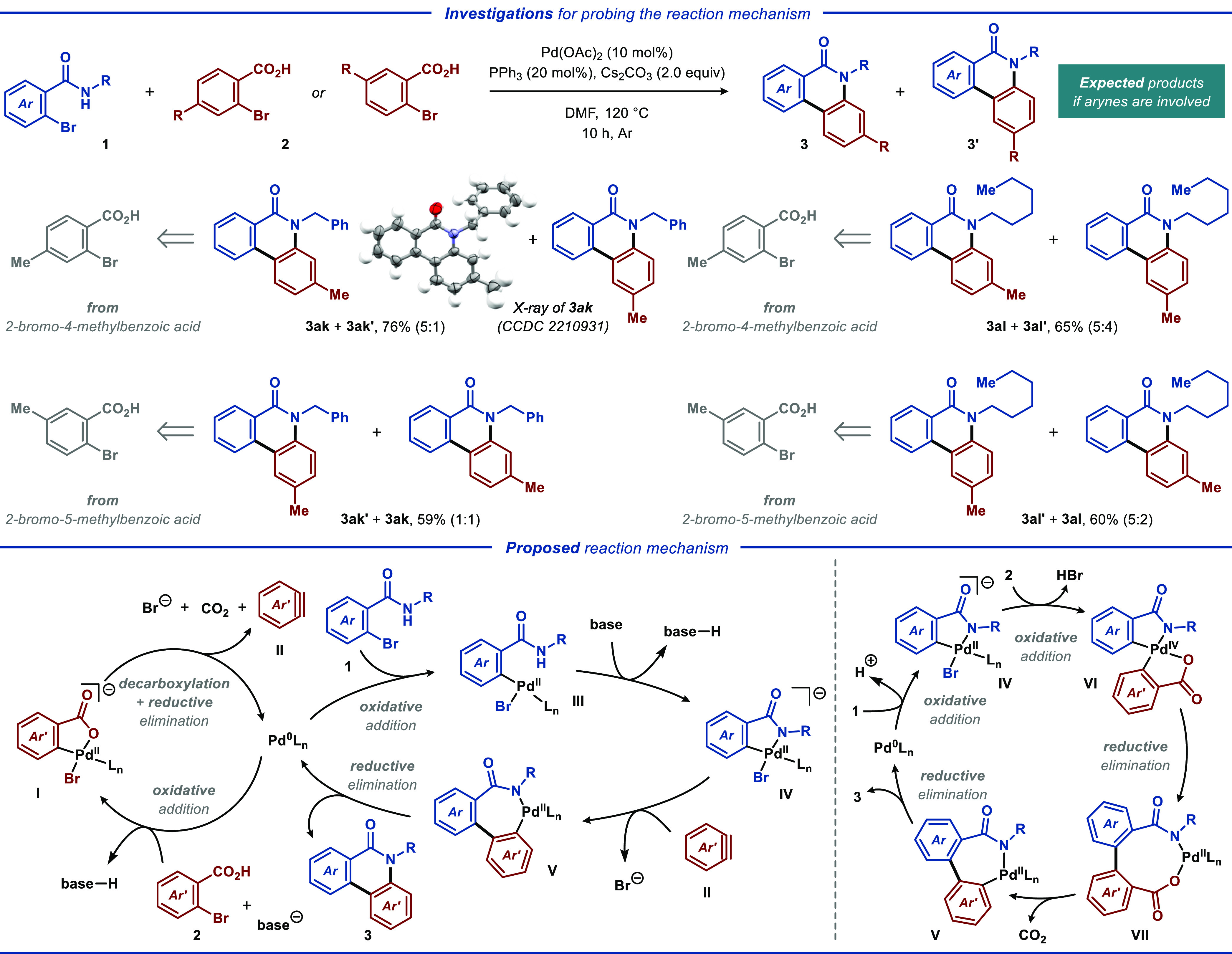
Investigations for Probing the Reaction Mechanism and Proposed Reaction Mechanism^,^ Reaction conditions: Reactions were carried out with **1** (0.50 mmol), **2** (0.60 mmol), Pd(OAc)_2_ (12 mg, 0.05 mmol), PPh_3_ (26 mg, 0.10 mmol), and Cs_2_CO_3_ (326 mg, 1.0 mmol) in DMF (5.0 mL) under argon at 120 °C for 10 h. Isolated product yields are reported. Regioisomeric ratios were determined by ^1^H NMR analysis.

The synthetic versatility of the developed protocol was further explored by employing 2-bromobenzamides **1** with a range of substituents at the aromatic core (Scheme 1). The reactions with 2-bromobenzamides **1** substituted with various aliphatic, chloro, and fluoro groups all provided the expected annulation products **3u**–**3af** in moderate to high yields (62–79%). Next, the scope of compatible 2-bromobenzoic acid annulation partners **2** was evaluated (Schemes 1 and 2). Here, 4,5-dimethoxy-2-bromobenzoic acid (**2b**) underwent effective annulation with *N*-substituted 2-bromobenzamides to produce **3ag**–**3aj** in high yields (84–88%, Scheme 1). Finally, the disclosed protocol was successfully applied to access quinolone-derived alkaloid phenaglydon (**4**). Thus, subjecting annulation product **3u** to refluxing trifluoroacetic acid afforded the debenzylated product phenaglydone (**4**) in an excellent yield of 92% (Scheme 1).

To probe the reaction mechanism, a set of control reactions were carried out under the optimized reaction conditions. When 4- or 5-substituted 2-bromobenzoic acids were used as the coupling partners, the respective annulated products were obtained as mixtures of two regioisomers (Scheme 2, *top*). Such poor regioselectivity indicates that the reaction proceeds through arynes as the key intermediates, as has been proposed for related transformations featuring palladium catalysis.^18^ Based on the literature precedents,^19^ a plausible mechanism that does not contradict the above control reactions is proposed (Scheme 2, *bottom left*). Initially, base-assisted oxidative addition of 2-bromobenzoic acid **2** to Pd^0^ provides the key aryl-Pd^II^ species **I**. This species undergoes extrusion of CO_2_ to afford aryne intermediate **II** while regenerating Pd^0^ and completing the first of the catalytic cycles. Meanwhile, the second of the catalytic cycles is onset by oxidative addition of the Pd^0^ catalyst to 2-bromobenzamide **1** to give aryl-Pd^II^ species **III**, which in the presence of a base furnishes the five-membered palladacycle **IV**. Insertion of previously produced aryne **II** into the Pd^II^–C bond of **IV** results in C–C bond formation, while subsequent reductive elimination from the seven-membered palladacycle **V** forges the desired C–N bond. Thereby, the latter step regenerates the Pd^0^ catalyst, concluding the second of the catalytic cycles, and furnishes the desired annulation product **3**. An alternative mechanism proceeding without formation of an aryne intermediate features a single catalytic cycle and Pd^IV^ species as the key intermediate (Scheme 2, *bottom right*).^20^ Here, the reaction is onset by oxidative addition of 2-bromobenzamide **1** to the Pd^0^ catalyst, furnishing aryl-Pd^II^ intermediate **IV**. In the key step of the reaction, this intermediate undergoes a second oxidative addition reaction to 2-bromobenzoic acid **2**, producing diaryl-Pd^IV^ species **VI**. Subsequently, this species undergoes reductive elimination to produce the biaryl Pd^II^-metallacycle **VII**, which eliminates CO_2_ to furnish the Pd^II^ intermediate **V**. Finally, the latter intermediate undergoes reductive elimination, concluding the catalytic cycle and furnishing desired product **3**.

## Conclusions

In conclusion, we disclosed a simple procedure for accessing phenanthridin-6(5*H*)-one derivatives through palladium-mediated annulation of 2-bromobenzamides and 2-bromobenzoic acids. The annulation reaction delivers the phenanthridin-6(5*H*)-one derivatives in high yields and is compatible with a variety of functional groups, providing a modular method for accessing a range of structurally diversified phenanthridin-6(5*H*)-one motifs.

## Experimental Section

### General Information

All reagents were purchased from commercial sources and used without treatment, unless otherwise indicated. The products were purified by column chromatography over silica gel. ^1^H NMR and ^13^C NMR spectra were recorded at 25 °C on a Varian spectrometer at 400 and 101 MHz, respectively, with TMS as the internal standard. High-resolution mass spectra (HRMS) were recorded on a BRUKER AutoflexIII Smartbeam mass spectrometer. High-resolution mass spectra (HRMS) were recorded on a Bruker microTof using electrospray ionization (ESI).

### General Procedure for the Synthesis of Phenanthridinones 3

To a 10 mL Schlenk tube equipped with a magnetic stir bar were added 2-bromobenzamide **1** (0.500 mmol, 1.00 equiv), *o*-bromobenzoic acid **2** (0.750 mmol, 1.50 equiv), DMF (4.0 mL), Cs_2_CO_3_ (163 mg, 0.500 mmol, 1.00 equiv), PPh_3_ (26 mg, 0.100 mmol, 0.200 equiv), and Pd(OAc)_2_ (11 mg, 0.05 mmol, 0.100 equiv). The reaction mixture was stirred at 120 °C in an oil bath for about 10 h. The resulting mixture was concentrated, and the residue was taken up in ethyl acetate. The organic layer was washed with brine, dried over Na_2_SO_4_, and concentrated. Purification of the crude product by column chromatography (silica gel; petroleum ether/ethyl acetate 30:1) afforded **3**.

## Data Availability

The data underlying this study are available in the published article and its online Supporting Information.

## Supporting Information Available

Experimental procedures, characterization data, and copies of NMR spectra for all obtained products (PDF)
FAIR data, including the primary NMR FID files, for compounds **3a**–**3z**, **3aa**–**3al**, and **4** (ZIP)
