# Supplementary material for: Construction of Phenanthridinone Skeletons through Palladium-Catalyzed Annulation
Source: J Org Chem. 2023 Aug 23;88(17):12738–43. doi: 10.1021/acs.joc.3c01429 (PMC10476191; doi:10.1021/acs.joc.3c01429)
Supplement: Supplementary file 1 — jo3c01429_si_001.pdf [file jo3c01429_si_001.pdf]

Supporting Information

Construction of Phenanthridinone Skeletons through Palladium-Catalyzed Annulation

Xin Geng,[†] Heng He,[†] Andrey Shatskiy,[‡] Elena V. Stepanova,[‡,‖] Gregory R. Alvey,[‡] Jian-Quan Liu,[†,‡,*] Markus D. Kärkäs,[‡,*] Xiang-Shan Wang[†,*]

† School of Chemistry and Materials Science, Jiangsu Normal University, Xuzhou, Jiangsu 221116, China

‡ Department of Chemistry, KTH Royal Institute of Technology, SE-100 44 Stockholm, Sweden

‖ Tomsk Polytechnic University, Lenin Avenue 30, 634050 Tomsk, Russia

* E-mail: liujq316@jsnu.edu.cn (J.-Q.L.); karkas@kth.se (M.D.K.); xswang@jsnu.edu.cn (X.-S.W)

Table of Contents

I. General information **S1**

II. Crystallography **S1**

III. Synthesis and analytical data of compounds 1, 3 and 4 .... **S4**

General procedure for preparation of 2-bromo-*N*-isopropylbenzamide S4

5-Methylphenanthridin-6(5*H*)-one (**3a**) S4

1 mmol Scale synthesis of benzo[4,5]imidazo[1,2-*f*]phenanthridine (**3a**) S5

5-Ethylphenanthridin-6(5*H*)-one (**3b**) S5

5-Propylphenanthridin-6(5*H*)-one (**3c**) S6

5-Isopropylphenanthridin-6(5*H*)-one (**3d**) S6

5-Butylphenanthridin-6(5*H*)-one (**3e**) S7

5-Isobutylphenanthridin-6(5*H*)-one (**3f**) S8

5-Hexylphenanthridin-6(5*H*)-one (**3h**) S8

5-(2-Ethylhexyl)phenanthridin-6(5*H*)-one (**3i**) S9

5-Benzylphenanthridin-6(5*H*)-one (**3j**) S10

5-(2-Chlorobenzyl)phenanthridin-6(5*H*)-one (**3k**) S10

5-(4-Methylbenzyl)phenanthridin-6(5*H*)-one (**3l**) S11

5-(4-Methoxybenzyl)phenanthridin-6(5*H*)-one (**3m**) S12

4-((6-Oxophenanthridin-5(6*H*)-yl)methyl)benzonitrile (**3n**) S12

5-(4-Fluorobenzyl)phenanthridin-6(5*H*)-one (**3o**) S13

5-(4-Chlorobenzyl)phenanthridin-6(5*H*)-one (**3p**) S14

5-(4-Methoxyphenethyl)phenanthridin-6(5*H*)-one (**3q**) S14

5-(Furan-2-ylmethyl)phenanthridin-6(5*H*)-one (**3r**) S15

5-(2-(Thiophen-2-yl)ethyl)phenanthridin-6(5*H*)-one (**3s**) S16

5-(2-(Benzo[*d*][1,3]dioxol-5-yl)ethyl)phenanthridin-6(5*H*)-one (**3t**) S16

5-Benzyl-8-methylphenanthridin-6(5*H*)-one (**3u**) S17

5-(2-Chlorobenzyl)-8-methylphenanthridin-6(5*H*)-one (**3v**) S18

8-Methyl-5-(4-methylbenzyl)phenanthridin-6(5*H*)-one (**3w**) S18

5-Benzyl-8-fluorophenanthridin-6(5*H*)-one (**3x**) S19

5-(2-Chlorobenzyl)-8-fluorophenanthridin-6(5*H*)-one (**3y**) S20

8-Fluoro-5-(4-methoxybenzyl)phenanthridin-6(5*H*)-one (**3z**) S20

5-Benzyl-9-methylphenanthridin-6(5*H*)-one (**3aa**) S21

5-(2-Chlorobenzyl)-9-methylphenanthridin-6(5*H*)-one (**3ab**) S22

5-(4-Methoxybenzyl)-9-methylphenanthridin-6(5*H*)-one (**3ac**) S22

5-Benzyl-9-chlorophenanthridin-6(5*H*)-one (**3ad**) S23

9-Chloro-5-(2-chlorobenzyl)phenanthridin-6(5*H*)-one (**3ae**) S24

9-Chloro-5-(4-methylbenzyl)phenanthridin-6(5*H*)-one (**3af**) S24

5-Benzyl-2,3-dimethoxyphenanthridin-6(5*H*)-one (**3ag**) S25

2,3-Dimethoxy-5-propylphenanthridin-6(5*H*)-one (**3ah**) S26

2,3-Dimethoxy-5-(4-methoxyphenethyl)phenanthridin-6(5*H*)-one (**3ai**) S26

2,3-Dimethoxy-5-(4-methoxyphenethyl)phenanthridin-6(5*H*)-one (**3aj**) S27

5-Benzyl-3-methylphenanthridin-6(5*H*)-one & 5-Benzyl-2-methylphenanthridin-6(5*H*)-one (**3ak**, **3ak'**) S28

5-Hexyl-3-methylphenanthridin-6(5*H*)-one & 5-Hexyl-2-methylphenanthridin-6(5*H*)-one (**3al**, **3al'**) S29

8-Methylphenanthridin-6(5*H*)-one (**4**) S30

IV. NMR spectra S32

5-Methylphenanthridin-6(5*H*)-one (**3a**) S32

5-Ethylphenanthridin-6(5*H*)-one (**3b**) S33

5-Propylphenanthridin-6(5*H*)-one (**3c**) S34

5-Isopropylphenanthridin-6(5*H*)-one (**3d**) S35

5-Butylphenanthridin-6(5*H*)-one (**3e**) S36

5-Isobutylphenanthridin-6(5*H*)-one (**3f**) S37

5-Hexylphenanthridin-6(5*H*)-one (**3h**) S38

5-(2-Ethylhexyl)phenanthridin-6(5*H*)-one (**3i**) S39

5-Benzylphenanthridin-6(5*H*)-one (**3j**) S40

5-(2-Chlorobenzyl)phenanthridin-6(5*H*)-one (**3k**) S41

5-(4-Methylbenzyl)phenanthridin-6(5*H*)-one (**3l**) S42

5-(4-Methoxybenzyl)phenanthridin-6(5*H*)-one (**3m**) S43

4-((6-Oxophenanthridin-5(6*H*)-yl)methyl)benzonitrile (**3n**) S44

5-(4-Fluorobenzyl)phenanthridin-6(5*H*)-one (**3o**) S45

5-(4-Chlorobenzyl)phenanthridin-6(5*H*)-one (**3p**) S46

5-(4-Methoxyphenethyl)phenanthridin-6(5*H*)-one (**3q**) S47

5-(Furan-2-ylmethyl)phenanthridin-6(5*H*)-one (**3r**) S48

5-(2-(Thiophen-2-yl)ethyl)phenanthridin-6(5*H*)-one (**3s**) S49

5-(2-(Benzo[*d*][1,3]dioxol-5-yl)ethyl)phenanthridin-6(5*H*)-one (**3t**) S50

5-Benzyl-8-methylphenanthridin-6(5*H*)-one (**3u**) S51

5-(2-Chlorobenzyl)-8-methylphenanthridin-6(5*H*)-one (**3v**) S52

8-Methyl-5-(4-methylbenzyl)phenanthridin-6(5*H*)-one (**3w**) S53

5-Benzyl-8-fluorophenanthridin-6(5*H*)-one (**3x**) S54

5-(2-Chlorobenzyl)-8-fluorophenanthridin-6(5*H*)-one (**3y**) S55

8-Fluoro-5-(4-methoxybenzyl)phenanthridin-6(5*H*)-one (**3z**) S56

5-Benzyl-9-methylphenanthridin-6(5*H*)-one (**3aa**) S57

5-(2-Chlorobenzyl)-9-methylphenanthridin-6(5*H*)-one (**3ab**) S58

5-(4-Methoxybenzyl)-9-methylphenanthridin-6(5*H*)-one (**3ac**) S59

5-Benzyl-9-chlorophenanthridin-6(5*H*)-one (**3ad**) S60

9-Chloro-5-(2-chlorobenzyl)phenanthridin-6(5*H*)-one (**3ae**) S61

9-Chloro-5-(4-methylbenzyl)phenanthridin-6(5*H*)-one (**3af**) S62

5-Benzyl-2,3-dimethoxyphenanthridin-6(5H)-one (**3ag**) S63

2,3-Dimethoxy-5-propylphenanthridin-6(5*H*)-one (**3ah**) S64

2,3-Dimethoxy-5-(4-methoxyphenethyl)phenanthridin-6(5*H*)-one (**3ai**) S65

2,3-Dimethoxy-5-(4-methoxyphenethyl)phenanthridin-6(5*H*)-one (**3aj**) S66

5-Benzyl-3-methylphenanthridin-6(5*H*)-one & 5-Benzyl-2-methylphenanthridin-6(5*H*)-one (**3ak**, **3ak'**) S67

5-Hexyl-3-methylphenanthridin-6(5*H*)-one & 5-Hexyl-2-methylphenanthridin-6(5*H*)-one (**3al**, **3al'**) S69

8-Methylphenanthridin-6(5*H*)-one (**4**) S71

V. References S72

I. General information

All reagents were purchased from commercial sources and used without treatment unless otherwise indicated. The products were purified by column chromatography over silica gel. ^{1}H NMR and ^{13}C NMR spectra were recorded at 25 °C on a Varian spectrometer at 400 MHz and 101 MHz, respectively, with TMS as the internal standard. High-resolution mass spectra (HRMS) were recorded on a BRUKER AutoflexIII Smartbeam mass spectrometer. High resolution mass spectra (HRMS) were recorded on a Bruker microTof using electrospray ionization (ESI).

II. Crystallography

Compound **3j** (50 mg) was dissolved in a centrifuge tube in 150 µL $CDCl_3$. Upon standing for several days (seven days), crystals suitable for X-ray diffraction of **3j** and **3ak** were obtained. The structures of the *N*-fused heterocyclic scaffolds were further established by X-ray diffraction. Single-crystal X-ray diffraction data for the reported compound was recorded at a temperature of 296(2) K on an Oxford Diffraction Gemini R Ultra diffractometer using a ω scan technique with Mo-Kα radiation (λ = 0.71073 Å). The structures were solved by the Direct Method of SHELXS-97 and refined by full-matrix least-squares techniques using the SHELXL-97 program.[1] Non-hydrogen atoms were refined with anisotropic temperature parameters, and hydrogen atoms of the ligands were refined as rigid groups. Basic information pertaining to crystal parameters and structure refinement is summarized in Tables S1 and S2.

Table S1. Crystal structure and refinement data for compound **3j** (thermal ellipsoids at 30% probability).

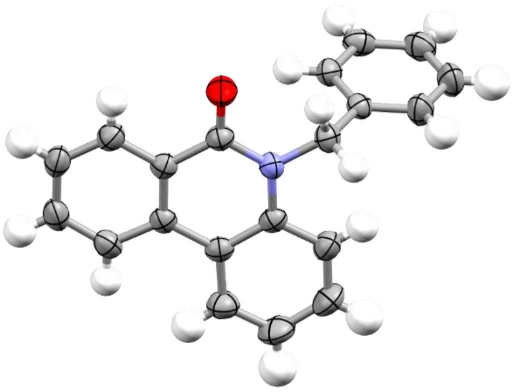

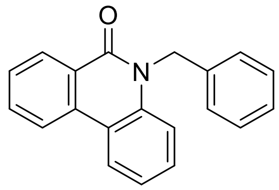

Empirical formula	$C_{20}H_{15}NO$
Temperature	296(2) K
Wavelength	0.71073 Å
Space group	P2(1)/c
Unit cell dimensions	a = 11.0901(14) Å<br>b = 16.076(2) Å<br>c = 8.5187(10) Å<br>alpha = 90 deg.<br>beta = 107.462(2) deg.<br>gamma = 90 deg.
Volume	1448.8(3) $Å^3$
Z	4
Calculated density	1.308 Mg/m^3
Absorption coefficient	0.080 mm^{-1}
F(000)	600
Theta range for data collection	2.809 to 25.018 deg.
Reflections collected / unique	7214 / 2542 [R(int) = 0.0214]
Data / restraints / parameters	2542 / 0 / 200
Goodness-of-fit on F^2	1.033
Final R indices [I>2sigma(I)]	R1 = 0.0362, wR2 = 0.0965
R indices (all data)	R1 = 0.0444, wR2 = 0.1042

Table S2. Crystal structure and refinement data for compound **3ak** (thermal ellipsoids at 30% probability).

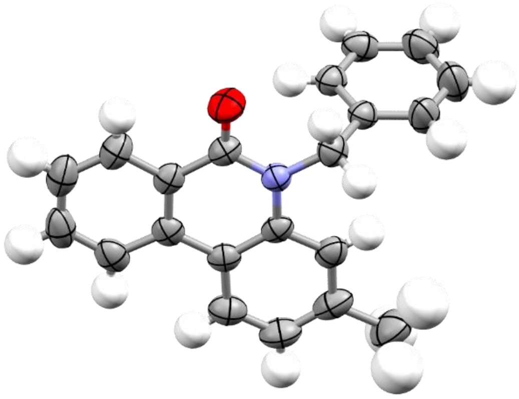

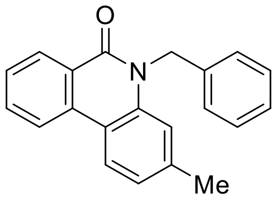

Empirical formula	$C_{30}H_{24}N_2O_2$
Temperature	296(2) K
Wavelength	0.71073 Å
Space group	P2(1)/c
Unit cell dimensions	a = 11.879(8) Å<br>b = 15.051(11) Å<br>c = 9.058(4) Å<br>alpha = 90 deg.<br>beta = 105.835(9) deg.<br>gamma = 90 deg.
Volume	1558.1(19) $Å^3$
Z	4
Calculated density	1.276 Mg/m^3
Absorption coefficient	0.078 mm^{-1}
F(000)	632
Crystal size	0.145 x 0.126 x 0.098 mm
Theta range for data collection	2.707 to 25.009 deg.
Reflections collected / unique	7800 / 2738 [R(int) = 0.0201]
Data / restraints / parameters	2738 / 0 / 208
Goodness-of-fit on F^2	1.058
Final R indices [I>2sigma(I)]	R1 = 0.0539, wR2 = 0.1574
R indices (all data)	R1 = 0.0682, wR2 = 0.1698

III. Synthesis and analytical data of compounds 1, 3 and 4

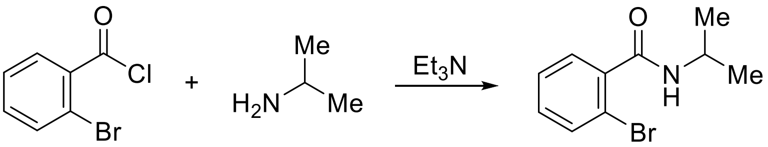

General procedure for preparation of 2-bromo-*N*-isopropylbenzamide:[1] To a dry round-bottom flask (100 mL), isopropylamine (2.1 g, 10 mmol) and triethylamine (25 mL) were added. The reaction was stirred in an ice water bath and *o*-bromobenzoyl chloride (2.2 g, 10 mmol) was slowly added dropwise to the round-bottom flask. The resulting mixture was stirred in an ice water bath for 30 min, and then stirred at ambient temperature for 2 hours. Then, the mixture was poured into a beaker with water, let to stand to allow formation of solids, and then filtered to obtain the desired amide.

Note: All *o*-bromobenzoic acids 2 are commercially available.

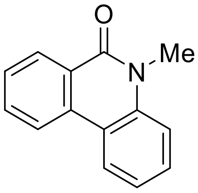

5-Methylphenanthridin-6(5*H*)-one (3a)[1]

To a 10 mL Schlenk tube equipped with a magnetic stir bar was added 2-bromo-*N*-methylbenzamide **1a** (107 mg, 0.500 mmol, 1.00 equiv), *o*-bromobenzoic acid **2a** (151 mg, 0.750 mmol, 1.50 equiv), DMF (4.0 mL), Cs_2CO_3 (163 mg, 0.500 mmol, 1.00 equiv), PPh_3 (26 mg, 0.100 mmol, 0.200 equiv), $Pd(OAc)_2$ (11 mg, 0.05 mmol, 0.100 equiv). The reaction mixture was stirred at 120 °C in an oil bath for about 10 h. The resulting mixture was concentrated and the residue was taken up in ethyl acetate. The organic layer was washed with brine, dried over Na_2SO_4 and concentrated. Purification of the crude product by column chromatography (silica gel; petroleum ether/ethyl acetate 30:1) afforded **3a** in 75% yield (78 mg).

White solid; mp 147–148 °C; ^1H NMR ($CDCl_3$, 400 MHz): δ_H 8.55 (d, J = 8.0 Hz, 1H), 8.32–8.25 (m, 2H), 7.76 (t, J = 7.6 Hz, 1H), 7.61–7.53 (m, 2H), 7.42 (d, J = 8.4 Hz, 1H), 7.33 (t, J = 7.6 Hz, 1H), 3.82 (s, 3H); $^{13}C\{^1H\}$ NMR ($CDCl_3$, 101 MHz): δ_C 161.7, 138.0, 133.5, 132.4, 129.6, 128.9, 127.9, 125.6, 123.2, 122.5, 121.6, 119.3, 115.0, 30.0; HRMS (ESI-TOF, m/z): calcd for $C_{14}H_{12}NO$ $[M + H]^+$, 210.0913; found, 210.0922.

1 mmol Scale synthesis of benzo[4,5]imidazo[1,2-*f*]phenanthridine (3a)

To a 25 mL Schlenk tube equipped with a magnetic stir bar was added 2-bromo-*N*-methylbenzamide **1a** (214 mg, 1.00 mmol, 1.00 equiv), *o*-bromobenzoic acid **2a** (302 mg, 1.50 mmol, 1.50 equiv), DMF (8 mL), Cs_2CO_3 (326 mg, 1.00 mmol, 1.00 equiv), PPh_3 (52 mg, 0.20 mmol, 0.20 equiv), $Pd(OAc)_2$ (22 mg, 0.2 mmol, 0.40 equiv). The reaction mixture was stirred at 120 °C in an oil bath for about 10 h. The resulting mixture was concentrated and the residue was taken up in ethyl acetate. The organic layer was washed with brine, dried over Na_2SO_4 and concentrated. Purification of the crude product by column chromatography (silica gel; petroleum ether/ethyl acetate 30:1) afforded **3a** as white solid in 71% yield (148 mg).

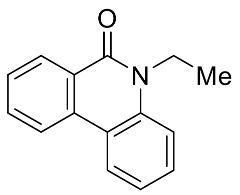

5-Ethylphenanthridin-6(5*H*)-one (3b)[2]

To a 10 mL Schlenk tube equipped with a magnetic stir bar was added 2-bromo-*N*-ethylbenzamide **1b** (114 mg, 0.500 mmol, 1.00 equiv), *o*-bromobenzoic acid **2a** (151 mg, 0.750 mmol, 1.50 equiv), DMF (4.0 mL), Cs_2CO_3 (163 mg, 0.500 mmol, 1.00 equiv), PPh_3 (26 mg, 0.100 mmol, 0.200 equiv), $Pd(OAc)_2$ (11 mg, 0.05 mmol, 0.100 equiv). The reaction mixture was stirred at 120 °C in an oil bath for about 10 h. The resulting mixture was concentrated and the residue was taken up in ethyl acetate. The organic layer was washed with brine, dried over Na_2SO_4 and concentrated. Purification of the crude product by column chromatography (silica gel; petroleum ether/ethyl acetate 30:1) afforded **3b** in 67% yield (75 mg).

White solid; mp 87–89 °C; ^{1}H NMR ($CDCl_3$, 400 MHz): δ_H 8.62 (d, *J* = 7.6 Hz, 1H), 8.39–8.34 (m, 2H), 7.82 (t, *J* = 8.0 Hz, 1H), 7.67–7.60 (m, 2H), 7.51 (d, *J* = 8.4 Hz, 1H), 7.38 (t, *J* = 8.0 Hz, 1H), 4.54 (q, *J* = 6.8 Hz, 2H), 1.48 (t, *J* = 7.2 Hz, 3H); $^{13}C\{^1H\}$ NMR ($CDCl_3$, 101 MHz): δ_C 161.1, 136.9, 133.5, 132.3, 129.5, 128.7, 127.9, 125.6, 123.5, 122.2, 121.5, 119.5, 114.9, 37.7, 12.7; HRMS (ESI-TOF, *m/z*): calcd for $C_{15}H_{14}NO$ $[M + H]^+$, 224.1070; found, 224.1071.

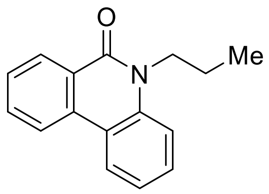

5-Propylphenanthridin-6(5*H*)-one (3c)[3]

To a 10 mL Schlenk tube equipped with a magnetic stir bar was added 2-bromo-*N*-propylbenzamide **1c** (121 mg, 0.500 mmol, 1.00 equiv), *o*-bromobenzoic acid **2a** (151 mg, 0.750 mmol, 1.50 equiv), DMF (4.0 mL), Cs_2CO_3 (163 mg, 0.500 mmol, 1.00 equiv), PPh_3 (26 mg, 0.100 mmol, 0.200 equiv), $Pd(OAc)_2$ (11 mg, 0.05 mmol, 0.100 equiv). The reaction mixture was stirred at 120 °C in an oil bath for about 10 h. The resulting mixture was concentrated and the residue was taken up in ethyl acetate. The organic layer was washed with brine, dried over Na_2SO_4 and concentrated. Purification of the crude product by column chromatography (silica gel; petroleum ether/ethyl acetate 30:1) afforded **3c** in 73% yield (86 mg).

White oil; ^{1}H NMR (CDCl$_3$, 400 MHz): δ_H 8.56 (d, *J* = 8.0 Hz, 1H), 8.29 (t, *J* = 9.2 Hz, 2H), 7.76 (t, *J* = 7.2 Hz, 1H), 7.61–7.52 (m, 2H), 7.41 (d, *J* = 8.4 Hz, 1H), 7.31 (t, *J* = 7.6 Hz, 1H), 4.36 (t, *J* = 7.6 Hz, 2H), 1.90–1.80 (m, 2H), 1.09 (t, *J* = 7.2 Hz, 3H); ^{13}C{^{1}H} NMR (CDCl$_3$, 101 MHz): δ_C 161.4, 137.1, 133.6, 132.4, 129.5, 128.8, 127.9, 125.5, 123.4, 122.2, 121.5, 119.5, 115.1, 44.2, 20.7, 11.4; HRMS (ESI-TOF, *m/z*): calcd for $C_{16}H_{16}NO$ [M + H]$^+$, 238.1226; found, 238.1241.

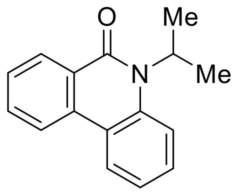

5-Isopropylphenanthridin-6(5*H*)-one (3d)[2]

To a 10 mL Schlenk tube equipped with a magnetic stir bar was added 2-bromo-*N*-isopropylbenzamide **1d** (121 mg, 0.500 mmol, 1.00 equiv), *o*-bromobenzoic acid **2a** (151 mg, 0.750 mmol, 1.50 equiv), DMF (4.0 mL), Cs_2CO_3 (163 mg, 0.500 mmol, 1.00 equiv), PPh_3 (26 mg, 0.100 mmol, 0.200 equiv), $Pd(OAc)_2$ (11 mg, 0.05 mmol, 0.100 equiv). The reaction mixture was stirred at 120 °C in an oil bath for about 10 h. The resulting mixture was concentrated and the residue was taken up in ethyl acetate. The organic layer was washed with brine, dried over Na_2SO_4 and concentrated. Purification of the crude

product by column chromatography (silica gel; petroleum ether/ethyl acetate 30:1) afforded **3d** in 61% yield (72 mg).

White solid; mp 99–101 °C; ^{1}H NMR ($CDCl_3$, 400 MHz): δ_H 8.52 (d, *J* = 7.6 Hz, 1H), 8.29 (d, *J* = 8.0 Hz, 1H), 8.25 (d, *J* = 8.4 Hz, 1H), 7.74 (t, *J* = 7.6 Hz, 1H), 7.64–7.55 (m, 2H), 7.50 (t, *J* = 7.6 Hz, 1H), 7.29 (t, *J* = 7.6 Hz, 1H), 5.50 (br, 1H), 1.72 (d, *J* = 7.2 Hz, 6H); $^{13}C\{^1H\}$ NMR ($CDCl_3$, 101 MHz): δ_C 161.9, 137.3, 133.5, 132.2, 128.9, 128.7, 127.9, 126.4, 123.7, 122.1, 121.4, 120.1, 115.9, 19.9; HRMS (ESI-TOF, *m/z*): calcd for $C_{16}H_{16}NO$ $[M + H]^+$, 238.1226; found, 238.1241.

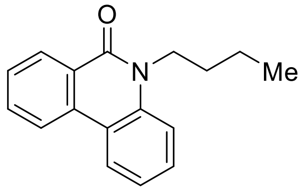

5-Butylphenanthridin-6(5*H*)-one (3e)[3]

To a 10 mL Schlenk tube equipped with a magnetic stir bar was added 2-bromo-*N*-butylbenzamide **1e** (128 mg, 0.500 mmol, 1.00 equiv), *o*-bromobenzoic acid **2a** (151 mg, 0.750 mmol, 1.50 equiv), DMF (4.0 mL), Cs_2CO_3 (163 mg, 0.500 mmol, 1.00 equiv), PPh_3 (26 mg, 0.100 mmol, 0.200 equiv), $Pd(OAc)_2$ (11 mg, 0.05 mmol, 0.100 equiv). The reaction mixture was stirred at 120 °C in an oil bath for about 10 h. The resulting mixture was concentrated and the residue was taken up in ethyl acetate. The organic layer was washed with brine, dried over Na_2SO_4 and concentrated. Purification of the crude product by column chromatography (silica gel; petroleum ether/ethyl acetate 30:1) afforded **3e** in 69% yield (86 mg).

White oil; ^{1}H NMR ($CDCl_3$, 400 MHz): δ_H 8.55 (d, *J* = 8.0 Hz, 1H), 8.28 (t, *J* = 8.8 Hz, 2H), 7.75 (t, *J* = 8.0 Hz, 1H), 7.60–7.52 (m, 2H), 7.41 (d, *J* = 8.4 Hz, 1H), 7.31 (t, *J* = 7.6 Hz, 1H), 4.40 (t, *J* = 7.6 Hz, 2H), 1.83–1.76 (m, 2H), 1.58–1.49 (m, 2H), 1.02 (t, *J* = 7.6 Hz, 3H); $^{13}C\{^1H\}$ NMR ($CDCl_3$, 101 MHz): δ_C 161.3, 137.1, 133.5, 132.3, 129.5, 128.8, 127.9, 125.5, 123.4, 122.2, 121.5, 119.5, 115.1, 42.5, 29.5, 20.4, 13.9; HRMS (ESI-TOF, *m/z*): calcd for $C_{17}H_{18}NO$ $[M + H]^+$, 252.1383; found, 252.1391.

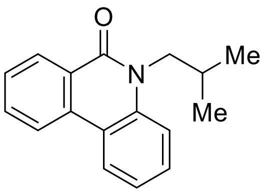

5-Isobutylphenanthridin-6(5*H*)-one (3f)

To a 10 mL Schlenk tube equipped with a magnetic stir bar was added 2-bromo-*N*-isobutylbenzamide **1f** (128 mg, 0.500 mmol, 1.00 equiv), *o*-bromobenzoic acid **2a** (151 mg, 0.750 mmol, 1.50 equiv), DMF (4.0 mL), Cs_2CO_3 (163 mg, 0.500 mmol, 1.00 equiv), PPh_3 (26 mg, 0.100 mmol, 0.200 equiv), $Pd(OAc)_2$ (11 mg, 0.05 mmol, 0.100 equiv). The reaction mixture was stirred at 120 °C in an oil bath for about 10 h. The resulting mixture was concentrated and the residue was taken up in ethyl acetate. The organic layer was washed with brine, dried over Na_2SO_4 and concentrated. Purification of the crude product by column chromatography (silica gel; petroleum ether/ethyl acetate 30:1) afforded **3f** in 66% yield (82 mg).

White oil; ^{1}H NMR ($CDCl_3$, 400 MHz): δ_H 8.56 (d, *J* = 7.6 Hz, 1H), 8.30 (t, *J* = 8.0 Hz, 2H), 7.76 (t, *J* = 7.6 Hz, 1H), 7.61–7.51 (m, 2H), 7.41 (d, *J* = 8.4 Hz, 1H), 7.33–7.26 (m, 1H), 4.32 (d, *J* = 3.6 Hz, 2H), 2.34–2.27 (m, 1H), 1.03 (d, *J* = 6.4 Hz, 6H); ^{13}C{^{1}H} NMR ($CDCl_3$, 101 MHz): δ_C 161.9, 137.4, 133.6, 132.4, 129.3, 129.1, 127.9, 125.5, 123.4, 122.2, 121.5, 119.5, 115.6, 49.0, 27.2, 20.2; HRMS (ESI-TOF, *m/z*): calcd for $C_{17}H_{18}NO$ $[M + H]^+$, 252.1383; found, 252.1391.

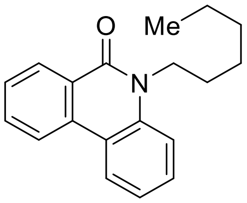

5-Hexylphenanthridin-6(5*H*)-one (3h)

To a 10 mL Schlenk tube equipped with a magnetic stir bar was added 2-bromo-*N*-cyclohexylbenzamide **1h** (141 mg, 0.500 mmol, 1.00 equiv), *o*-bromobenzoic acid **2a** (151 mg, 0.750 mmol, 1.50 equiv), DMF (4.0 mL), Cs_2CO_3 (163 mg, 0.500 mmol, 1.00 equiv), PPh_3 (26 mg, 0.100 mmol, 0.200 equiv), $Pd(OAc)_2$ (11 mg, 0.05 mmol, 0.100 equiv). The reaction mixture was stirred at 120 °C in an oil bath for about 10 h. The resulting mixture was concentrated and the residue was taken up in ethyl acetate. The organic layer was washed with brine, dried over Na_2SO_4 and concentrated.

Purification of the crude product by column chromatography (silica gel; petroleum ether/ethyl acetate 30:1) afforded **3h** in 74% yield (102 mg).

White oil; ^{1}H NMR ($CDCl_3$, 400 MHz): δ_H 8.56 (d, *J* = 8.0 Hz, 1H), 8.30 (t, *J* = 9.2 Hz, 2H), 7.76 (t, *J* = 7.2 Hz, 1H), 7.61–7.53 (m, 2H), 7.41 (d, *J* = 8.4 Hz, 1H), 7.32 (t, *J* = 7.6 Hz, 1H), 4.39 (t, *J* = 7.6 Hz, 2H), 1.85–1.77 (m, 2H), 1.54–1.47 (m, 2H), 1.38–1.36 (m, 4H), 0.91 (t, *J* = 6.8 Hz, 3H); ^{13}C{^{1}H} NMR ($CDCl_3$, 101 MHz): δ_C 161.3, 137.1, 133.5, 132.3, 129.5, 128.8, 127.9, 125.6, 123.4, 122.2, 121.5, 119.5, 115.1, 42.8, 31.6, 27.4, 26.8, 22.6, 14.0; HRMS (ESI-TOF, *m/z*): calcd for $C_{19}H_{22}NO$ [M + H]$^+$, 280.1696; found, 280.1688.

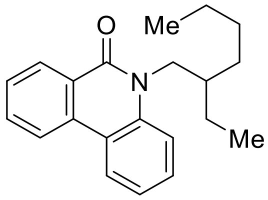

5-(2-Ethylhexyl)phenanthridin-6(5*H*)-one (3i)

To a 10 mL Schlenk tube equipped with a magnetic stir bar was added 2-bromo-*N*-(2-ethylhexyl)benzamide **1i** (156 mg, 0.500 mmol, 1.00 equiv), *o*-bromobenzoic acid **2a** (151 mg, 0.750 mmol, 1.50 equiv), DMF (4.0 mL), Cs_2CO_3 (163 mg, 0.500 mmol, 1.00 equiv), PPh_3 (26 mg, 0.100 mmol, 0.200 equiv), $Pd(OAc)_2$ (11 mg, 0.05 mmol, 0.100 equiv). The reaction mixture was stirred at 120 °C in an oil bath for about 10 h. The resulting mixture was concentrated and the residue was taken up in ethyl acetate. The organic layer was washed with brine, dried over Na_2SO_4 and concentrated. Purification of the crude product by column chromatography (silica gel; petroleum ether/ethyl acetate 30:1) afforded **3i** in 71% yield (109 mg).

White oil; ^{1}H NMR ($CDCl_3$, 400 MHz): δ_H 8.56 (d, *J* = 7.6 Hz, 1H), 8.30 (t, *J* = 7.6 Hz, 2H), 7.76 (t, *J* = 7.2 Hz, 1H), 7.61–7.51 (m, 2H), 7.43 (d, *J* = 8.4 Hz, 1H), 7.31 (t, *J* = 7.6 Hz, 1H), 4.77 (d, *J* = 5.6 Hz, 1H), 4.47 (s, 1H), 4.33 (s, 1H), 2.01–1.98 (m, 1H), 1.45–1.40 (m, 3H), 1.29–1.26 (m, 4H), 0.93 (t, *J* = 7.2 Hz, 3H), 0.87 (t, *J* = 6.0 Hz, 3H); ^{13}C{^{1}H} NMR ($CDCl_3$, 101 MHz): δ_C 162.0, 137.5, 133.6, 132.3, 129.3, 129.1, 127.9, 125.5, 123.4, 122.2, 121.5, 119.5, 115.6, 46.1, 37.6, 30.6, 28.8, 23.9, 23.1, 14.0, 10.8; HRMS (ESI-TOF, *m/z*): calcd for $C_{21}H_{26}NO$ [M + H]$^+$, 308.2009; found, 308.2002.

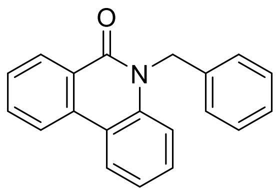

5-Benzylphenanthridin-6(5*H*)-one (3j)[2]

To a 10 mL Schlenk tube equipped with a magnetic stir bar was added *N*-benzyl-2-bromobenzamide **1j** (145 mg, 0.500 mmol, 1.00 equiv), *o*-bromobenzoic acid **2a** (151 mg, 0.750 mmol, 1.50 equiv), DMF (4.0 mL), Cs_2CO_3 (163 mg, 0.500 mmol, 1.00 equiv), PPh_3 (26 mg, 0.100 mmol, 0.200 equiv), $Pd(OAc)_2$ (11 mg, 0.05 mmol, 0.100 equiv). The reaction mixture was stirred at 120 °C in an oil bath for about 10 h. The resulting mixture was concentrated and the residue was taken up in ethyl acetate. The organic layer was washed with brine, dried over Na_2SO_4 and concentrated. Purification of the crude product by column chromatography (silica gel; petroleum ether/ethyl acetate 30:1) afforded **3j** in 78% yield (111 mg).

White solid; mp 126–129 °C; ^{1}H NMR ($CDCl_3$, 400 MHz): δ_H 8.63 (d, *J* = 8.0 Hz, 1H), 8.30 (t, *J* = 7.6 Hz, 2H), 7.80 (t, *J* = 7.6 Hz, 1H), 7.62 (t, *J* = 7.6 Hz, 1H), 7.40 (t, *J* = 8.0 Hz, 1H), 7.32–7.21 (m, 7H), 5.67 (s, 2H); ^{13}C{^{1}H} NMR ($CDCl_3$, 101 MHz): δ_C 161.9, 137.3, 136.6, 133.8, 132.7, 129.5, 129.2, 128.8, 128.0, 127.2, 126.5, 125.4, 123.3, 122.6, 121.7, 119.5, 116.0, 46.5; HRMS (ESI-TOF, *m/z*): calcd for $C_{20}H_{16}NO$ $[M + H]^+$, 286.1226; found, 286.1220.

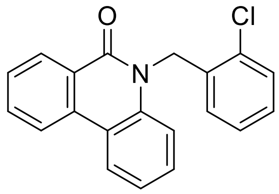

5-(2-Chlorobenzyl)phenanthridin-6(5*H*)-one (3k)

To a 10 mL Schlenk tube equipped with a magnetic stir bar was added 2-bromo-*N*-(2-chlorobenzyl)benzamide **1k** (162 mg, 0.500 mmol, 1.00 equiv), *o*-bromobenzoic acid **2a** (151 mg, 0.750 mmol, 1.50 equiv), DMF (4.0 mL), Cs_2CO_3 (163 mg, 0.500 mmol, 1.00 equiv), PPh_3 (26 mg, 0.100 mmol, 0.200 equiv), $Pd(OAc)_2$ (11 mg, 0.05 mmol, 0.100 equiv). The reaction mixture was stirred at 120 °C in an oil bath for about 10 h. The resulting mixture was concentrated and the residue was taken up in ethyl acetate. The organic layer was washed with brine, dried over Na_2SO_4 and concentrated.

Purification of the crude product by column chromatography (silica gel; petroleum ether/ethyl acetate 30:1) afforded **3k** in 82% yield (131 mg).

White crystals; mp 142–143 °C; ^{1}H NMR ($CDCl_3$, 400 MHz): δ_H 8.62 (d, *J* = 8.0 Hz, 1H), 8.32 (t, *J* = 7.6 Hz, 2H), 7.82 (t, *J* = 7.2 Hz, 1H), 7.63 (t, J = 7.6 Hz, 1H), 7.46–7.39 (m, 2H), 7.30 (t, *J* = 7.6 Hz, 1H), 7.18 (t, *J* = 7.7 Hz, 1H), 7.11–7.04 (m, 2H), 6.83 (d, *J* = 8.0 Hz, 1H), 5.72 (s, 2H); ^{13}C{^{1}H} NMR ($CDCl_3$, 101 MHz): δ_C 161.9, 137.0, 133.9, 133.5, 132.8, 132.6, 129.8, 129.6, 129.2, 128.4, 128.1, 127.2, 126.9, 125.3, 123.3, 122.8, 121.7, 119.5, 115.8, 44.3; HRMS (ESI-TOF, *m/z*): calcd for $C_{20}H_{15}ClNO$ [M + H]$^+$, 320.0837; found, 320.0822.

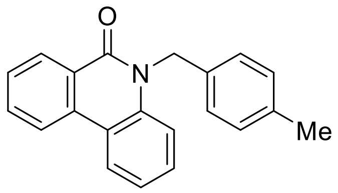

5-(4-Methylbenzyl)phenanthridin-6(5*H*)-one (3l)[2]

To a 10 mL Schlenk tube equipped with a magnetic stir bar was added 2-bromo-*N*-(4-methylbenzyl)benzamide **1l** (152 mg, 0.500 mmol, 1.00 equiv), *o*-bromobenzoic acid **2a** (151 mg, 0.750 mmol, 1.50 equiv), DMF (4.0 mL), Cs_2CO_3 (163 mg, 0.500 mmol, 1.00 equiv), PPh_3 (26 mg, 0.100 mmol, 0.200 equiv), $Pd(OAc)_2$ (11 mg, 0.05 mmol, 0.100 equiv). The reaction mixture was stirred at 120 °C in an oil bath for about 10 h. The resulting mixture was concentrated and the residue was taken up in ethyl acetate. The organic layer was washed with brine, dried over Na_2SO_4 and concentrated. Purification of the crude product by column chromatography (silica gel; petroleum ether/ethyl acetate 30:1) afforded **3l** in 70% yield (105 mg).

White solid; mp 106–108 °C; ^{1}H NMR ($CDCl_3$, 400 MHz): δ_H 8.62 (d, *J* = 8.0 Hz, 1H), 8.30 (t, *J* = 8.0 Hz, 2H), 7.79 (t, *J* = 7.6 Hz, 1H), 7.62 (t, *J* = 7.6 Hz, 1H), 7.42–7.38 (m, 1H), 7.33 (d, *J* = 8.4 Hz, 1H), 7.29–7.26 (m, 1H), 7.16 (d, *J* = 7.6 Hz, 2H), 7.10 (d, *J* = 8.0 Hz, 2H), 5.63 (s, 2H), 2.29 (s, 3H); ^{13}C{^{1}H} NMR ($CDCl_3$, 101 MHz): δ_C 161.9, 137.4, 136.8, 133.8, 133.5, 132.6, 129.5, 129.4, 129.2, 128.0, 126.5, 125.5, 123.2, 122.5, 121.7, 119.5, 116.0, 46.3, 21.0; HRMS (ESI-TOF, *m/z*): calcd for $C_{21}H_{17}NNaO$ [M + Na]$^+$, 322.1202; found, 322.1199.

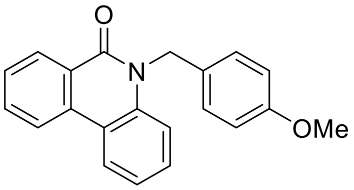

5-(4-Methoxybenzyl)phenanthridin-6(5*H*)-one (3m)[2]

To a 10 mL Schlenk tube equipped with a magnetic stir bar was added 2-bromo-*N*-(4-methoxybenzyl)benzamide **1m** (160 mg, 0.500 mmol, 1.00 equiv), *o*-bromobenzoic acid **2a** (151 mg, 0.750 mmol, 1.50 equiv), DMF (4.0 mL), Cs_2CO_3 (163 mg, 0.500 mmol, 1.00 equiv), PPh_3 (26 mg, 0.100 mmol, 0.200 equiv), $Pd(OAc)_2$ (11 mg, 0.05 mmol, 0.100 equiv). The reaction mixture was stirred at 120 °C in an oil bath for about 10 h. The resulting mixture was concentrated and the residue was taken up in ethyl acetate. The organic layer was washed with brine, dried over Na_2SO_4 and concentrated. Purification of the crude product by column chromatography (silica gel; petroleum ether/ethyl acetate 30:1) afforded **3m** in 66% yield (104 mg).

White solid; mp 136–137 °C; ^{1}H NMR ($CDCl_3$, 400 MHz): δ_H 8.62 (d, *J* = 8.0 Hz, 1H), 8.30 (t, *J* = 7.6 Hz, 2H), 7.79 (t, *J* = 7.6 Hz, 1H), 7.62 (t, *J* = 7.6 Hz, 1H), 7.43–7.35 (m, 2H), 7.29–7.20 (m, 3H), 6.83 (d, *J* = 8.4 Hz, 2H), 5.61 (s, 2H), 3.75 (s, 3H); ^{13}C{^{1}H} NMR ($CDCl_3$, 101 MHz): δ_C 161.9, 158.7, 137.4, 133.8, 132.6, 129.5, 129.1, 128.6, 128.0, 127.9, 125.5, 123.3, 122.5, 121.7, 119.5, 116.0, 114.2, 55.2, 45.9; HRMS (ESI-TOF, *m/z*): calcd for $C_{21}H_{17}N\ Na\ O_2$ $[M + Na]^+$, 338.1151; found, 338.1145.

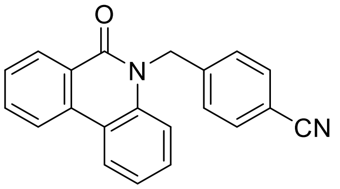

4-((6-Oxophenanthridin-5(6*H*)-yl)methyl)benzonitrile (3n)

To a 10 mL Schlenk tube equipped with a magnetic stir bar was added 2-bromo-*N*-(4-cyanobenzyl)benzamide **1n** (158 mg, 0.500 mmol, 1.00 equiv), *o*-bromobenzoic acid **2a** (151 mg, 0.750 mmol, 1.50 equiv), DMF (4.0 mL), Cs_2CO_3 (163 mg, 0.500 mmol, 1.00 equiv), PPh_3 (26 mg, 0.100 mmol, 0.200 equiv), $Pd(OAc)_2$ (11 mg, 0.05 mmol, 0.100 equiv). The reaction mixture was stirred at 120 °C in an oil bath for about 10 h. The resulting mixture was concentrated and the residue was taken up in ethyl acetate. The organic layer was washed with brine, dried over Na_2SO_4 and concentrated.

Purification of the crude product by column chromatography (silica gel; petroleum ether/ethyl acetate 30:1) afforded **3n** in 72% yield (111 mg).

White crystals; mp 162–163 °C; ^{1}H NMR ($CDCl_3$, 400 MHz): δ_H 8.60 (d, *J* = 8.0 Hz, 1H), 8.33 (d, *J* = 8.0 Hz, 2H), 7.83 (t, *J* = 7.6 Hz, 1H), 7.64 (t, *J* = 7.6 Hz, 1H), 7.60 (d, *J* = 8.0 Hz, 2H), 7.42 (t, *J* = 8.0 Hz, 1H), 7.38–7.30 (m, 3H), 7.16 (d, *J* = 8.4 Hz, 1H), 5.71 (s, 2H); ^{13}C{^{1}H} NMR ($CDCl_3$, 101 MHz): δ_C 161.9, 142.2, 136.9, 133.8, 133.0, 132.7, 129.7, 129.2, 128.3, 127.2, 125.1, 123.6, 123.0, 121.8, 119.6, 118.6, 115.4, 111.3, 46.2; HRMS (ESI-TOF, *m/z*): calcd for $C_{21}H_{15}N_2O$ [M + H]$^+$, 311.1179; found, 311.1187.

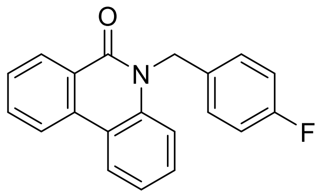

5-(4-Fluorobenzyl)phenanthridin-6(5*H*)-one (3o)

To a 10 mL Schlenk tube equipped with a magnetic stir bar was added 2-bromo-*N*-(4-fluorobenzyl)benzamide **1o** (154 mg, 0.500 mmol, 1.00 equiv), *o*-bromobenzoic acid **2a** (151 mg, 0.750 mmol, 1.50 equiv), DMF (4.0 mL), Cs_2CO_3 (163 mg, 0.500 mmol, 1.00 equiv), PPh_3 (26 mg, 0.100 mmol, 0.200 equiv), $Pd(OAc)_2$ (11 mg, 0.05 mmol, 0.100 equiv). The reaction mixture was stirred at 120 °C in an oil bath for about 10 h. The resulting mixture was concentrated and the residue was taken up in ethyl acetate. The organic layer was washed with brine, dried over Na_2SO_4 and concentrated. Purification of the crude product by column chromatography (silica gel; petroleum ether/ethyl acetate 30:1) afforded **3o** in 75% yield (114 mg).

White crystals; mp 137–139 °C; ^{1}H NMR ($CDCl_3$, 400 MHz): δ_H 8.61 (d, *J* = 8.0 Hz, 1H), 8.31 (d, *J* = 8.0 Hz, 1H), 8.29 (d, *J* = 8.4 Hz, 1H), 7.80 (t, *J* = 7.6 Hz, 1H), 7.62 (t, *J* = 7.6 Hz, 1).H), 7.41 (t, *J* = 7.6 Hz, 1H), 7.30–7.23 (m, 4H), 6.98 (t, *J* = 8.4 Hz, 2H), 5.62 (s, 2H); ^{13}C{^{1}H} NMR ($CDCl_3$, 101 MHz): δ_C 162.0 (d, $J_{C–F}$ = 244.0 Hz), 161.9, 137.2, 133.8, 132.8, 132.3 (d, $J_{C–F}$ = 3.0 Hz), 129.5, 129.1, 128.3, 128.1 (d, $J_{C–F}$ = 8.0 Hz), 125.3, 123.4, 122.7, 121.7, 119.5, 115.8, 115.6, 45.8; HRMS (ESI-TOF, *m/z*): calcd for $C_{20}H_{15}FNO$ [M + H]$^+$, 304.1132; found, 304.1138.

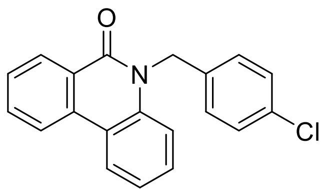

5-(4-Chlorobenzyl)phenanthridin-6(5*H*)-one (3p)

To a 10 mL Schlenk tube equipped with a magnetic stir bar was added 2-bromo-*N*-(4-chlorobenzyl)benzamide **1p** (162 mg, 0.500 mmol, 1.00 equiv), *o*-bromobenzoic acid **2a** (151 mg, 0.750 mmol, 1.50 equiv), DMF (4.0 mL), Cs_2CO_3 (163 mg, 0.500 mmol, 1.00 equiv), PPh_3 (26 mg, 0.100 mmol, 0.200 equiv), $Pd(OAc)_2$ (11 mg, 0.05 mmol, 0.100 equiv). The reaction mixture was stirred at 120 °C in an oil bath for about 10 h. The resulting mixture was concentrated and the residue was taken up in ethyl acetate. The organic layer was washed with brine, dried over Na_2SO_4 and concentrated. Purification of the crude product by column chromatography (silica gel; petroleum ether/ethyl acetate 30:1) afforded **3p** in 69% yield (110 mg).

White crystals; mp 133–134 °C; ^{1}H NMR ($CDCl_3$, 400 MHz): δ_H 8.61 (d, *J* = 8.0 Hz, 1H), 8.32–8.29 (m, 2H), 7.81 (t, *J* = 7.6 Hz, 1H), 7.63 (t, *J* = 7.6 Hz, 1H), 7.41 (t, *J* = 7.6 Hz, 1H), 7.31–7.20 (m, 6H), 5.63 (s, 2H); $^{13}C\{^1H\}$ NMR ($CDCl_3$, 101 MHz): δ_C 161.9, 137.1, 135.1, 133.8, 133.0, 132.8, 129.6, 129.1, 129.0, 128.1, 128.0, 125.3, 123.4, 122.7, 121.7, 119.5, 115.8, 45.9; HRMS (ESI-TOF, *m/z*): calcd for $C_{20}H_{15}ClNO$ $[M + H]^+$, 320.0837; found, 320.0822.

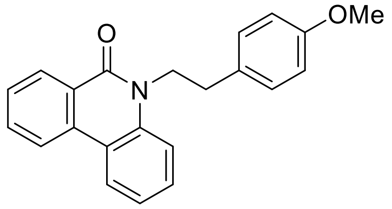

5-(4-Methoxyphenethyl)phenanthridin-6(5*H*)-one (3q)

To a 10 mL Schlenk tube equipped with a magnetic stir bar was added 2-bromo-*N*-(4-methoxyphenethyl)benzamide **1q** (167 mg, 0.500 mmol, 1.00 equiv), *o*-bromobenzoic acid **2a** (151 mg, 0.750 mmol, 1.50 equiv), DMF (4.0 mL), Cs_2CO_3 (163 mg, 0.500 mmol, 1.00 equiv), PPh_3 (26 mg, 0.100 mmol, 0.200 equiv), $Pd(OAc)_2$ (11 mg, 0.05 mmol, 0.100 equiv). The reaction mixture was stirred at 120 °C in an oil bath for about 10 h. The resulting mixture was concentrated and the residue was taken up in ethyl acetate. The organic layer was washed with brine, dried over Na_2SO_4 and concentrated.

Purification of the crude product by column chromatography (silica gel; petroleum ether/ethyl acetate 30:1) afforded **3q** in 70% yield (115 mg).

White crystals; mp 82–83 °C; ^{1}H NMR ($CDCl_3$, 400 MHz): δ_H 8.57 (d, *J* = 8.0 Hz, 1H), 8.34–8.29 (m, 2H), 7.78 (t, *J* = 7.2 Hz, 1H), 7.63–7.56 (m, 2H), 7.49 (d, *J* = 8.4 Hz, 1H), 7.36–7.32 (m, 3H), 6.90 (d, *J* = 8.0 Hz, 2H), 4.56 (t, *J* = 8.0 Hz, 2H), 3.82 (s, 3H), 3.04 (t, *J* = 8.0 Hz, 2H); ^{13}C{^{1}H} NMR ($CDCl_3$, 101 MHz): δ_C 161.3, 158.4, 137.0, 133.6, 132.5, 130.6, 129.8, 129.6, 128.8, 128.0, 125.5, 123.6, 122.4, 121.6, 119.5, 114.9, 114.1, 55.3, 44.5, 32.7; HRMS (ESI-TOF, *m/z*): calcd for $C_{22}H_{19}NNaO_2$ $[M + Na]^+$, 352.1308; found, 352.1312.

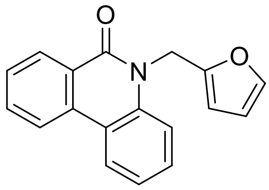

5-(Furan-2-ylmethyl)phenanthridin-6(5*H*)-one (3r)

To a 10 mL Schlenk tube equipped with a magnetic stir bar was added 2-bromo-*N*-(furan-2-ylmethyl)benzamide **1r** (140 mg, 0.500 mmol, 1.00 equiv), *o*-bromobenzoic acid **2a** (151 mg, 0.750 mmol, 1.50 equiv), DMF (4.0 mL), Cs_2CO_3 (163 mg, 0.500 mmol, 1.00 equiv), PPh_3 (26 mg, 0.100 mmol, 0.200 equiv), $Pd(OAc)_2$ (11 mg, 0.05 mmol, 0.100 equiv). The reaction mixture was stirred at 120 °C in an oil bath for about 10 h. The resulting mixture was concentrated and the residue was taken up in ethyl acetate. The organic layer was washed with brine, dried over Na_2SO_4 and concentrated. Purification of the crude product by column chromatography (silica gel; petroleum ether/ethyl acetate 30:1) afforded **3r** in 77% yield (106 mg).

White solid; mp 99–100 °C; ^{1}H NMR ($CDCl_3$, 400 MHz): δ_H 8.58 (d, *J* = 8.0 Hz, 1H), 8.27 (d, *J* = 8.0 Hz, 2H), 7.76 (t, *J* = 7.2 Hz, 1H), 7.64–7.57 (m, 2H), 7.52 (t, *J* = 7.2 Hz, 1H), 7.34–7.29 (m, 2H), 6.32 (d, *J* = 11.6 Hz, 2H), 5.61 (s, 2H); ^{13}C{^{1}H} NMR ($CDCl_3$, 101 MHz): δ_C 161.5, 150.3, 142.0, 137.2, 133.7, 132.7, 129.5, 129.0, 128.0, 125.4, 123.3, 122.6, 121.6, 119.5, 115.6, 110.5, 108.5, 39.7; HRMS (ESI-TOF, *m/z*): calcd for $C_{18}H_{13}NNaO_2$ $[M + Na]^+$, 298.0838; found, 298.0846.

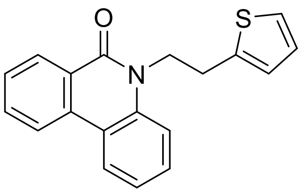

5-(2-(Thiophen-2-yl)ethyl)phenanthridin-6(5*H*)-one (3s)

To a 10 mL Schlenk tube equipped with a magnetic stir bar was added 2-bromo-*N*-(2-(thiophen-2-yl)ethyl)benzamide **1s** (155 mg, 0.500 mmol, 1.00 equiv), *o*-bromobenzoic acid **2a** (151 mg, 0.750 mmol, 1.50 equiv), DMF (4.0 mL), Cs_2CO_3 (163 mg, 0.500 mmol, 1.00 equiv), PPh_3 (26 mg, 0.100 mmol, 0.200 equiv), $Pd(OAc)_2$ (11 mg, 0.05 mmol, 0.100 equiv). The reaction mixture was stirred at 120 °C in an oil bath for about 10 h. The resulting mixture was concentrated and the residue was taken up in ethyl acetate. The organic layer was washed with brine, dried over Na_2SO_4 and concentrated. Purification of the crude product by column chromatography (silica gel; petroleum ether/ethyl acetate 30:1) afforded **3s** in 75% yield (114 mg).

White solid; mp 93–94 °C; ^{1}H NMR ($CDCl_3$, 400 MHz): δ_H 8.56 (d, *J* = 8.0 Hz, 1H), 8.36–8.29 (m, 2H), 7.78 (t, *J* = 7.2 Hz, 1H), 7.63–7.55 (m, 2H), 7.44 (d, *J* = 8.4 Hz, 1H), 7.34 (t, *J* = 8.0 Hz, 1H), 7.21–7.20 (m, 1H), 6.99–6.98 (m, 2H), 4.65 (t, *J* = 8.0 Hz, 2H), 3.32 (t, *J* = 7.6 Hz, 2H); ^{13}C{^{1}H} NMR ($CDCl_3$, 101 MHz): δ_C 161.3, 140.4, 136.9, 133.6, 132.6, 129.7, 128.8, 128.0, 127.1, 125.5, 125.4, 124.0, 123.6, 122.5, 121.6, 119.5, 114.7, 44.3, 27.6; HRMS (ESI-TOF, *m/z*): calcd for $C_{19}H_{15}NNaOS$ [M + Na]$^+$, 328.0767; found, 328.0775.

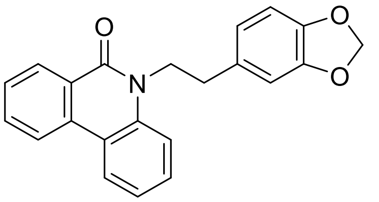

5-(2-(Benzo[*d*][1,3]dioxol-5-yl)ethyl)phenanthridin-6(5*H*)-one (3t)

To a 10 mL Schlenk tube equipped with a magnetic stir bar was added *N*-(2-(benzo[*d*][1,3]dioxol-5-yl)ethyl)-2-bromobenzamide **1t** (174 mg, 0.500 mmol, 1.00 equiv), *o*-bromobenzoic acid **2a** (151 mg, 0.750 mmol, 1.50 equiv), DMF (4.0 mL), Cs_2CO_3 (163 mg, 0.500 mmol, 1.00 equiv), PPh_3 (26 mg, 0.100 mmol, 0.200 equiv), $Pd(OAc)_2$ (11 mg, 0.05 mmol, 0.100 equiv). The reaction mixture was stirred at 120 °C in an oil bath for about 10 h. The resulting mixture was concentrated and the residue was taken up in ethyl acetate. The organic layer was washed with brine, dried over Na_2SO_4 and concentrated.

Purification of the crude product by column chromatography (silica gel; petroleum ether/ethyl acetate 30:1) afforded **3t** in 71% yield (122 mg).

White solid; mp 126–127 °C; ^{1}H NMR ($CDCl_3$, 400 MHz): δ_H 8.57 (d, *J* = 8.0 Hz, 1H), 8.34–8.29 (m, 2H), 7.78 (t, *J* = 7.6 Hz, 1H), 7.63–7.56 (m, 2H), 7.47 (d, *J* = 8.4 Hz, 1H), 7.34 (t, *J* = 7.6 Hz, 1H), 6.91 (s, 1H), 6.86–6.79 (m, 2H), 5.96 (s, 2H), 4.55 (t, *J* = 8.0 Hz, 2H), 3.01 (t, *J* = 8.0 Hz, 2H); $^{13}C\{^1H\}$ NMR ($CDCl_3$, 101 MHz): δ_C 161.3, 147.8, 146.3, 137.0, 133.6, 132.5, 132.3, 129.7, 128.8, 128.0, 125.5, 123.6, 122.4, 121.7, 121.6, 119.6, 114.8, 109.3, 108.5, 100.9, 44.5, 33.4; HRMS (ESI-TOF, *m/z*): calcd for $C_{22}H_{17}NNaO_3$ [M + Na]$^+$, 366.1101; found, 366.1109.

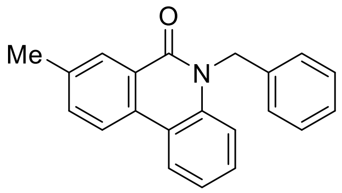

5-Benzyl-8-methylphenanthridin-6(5*H*)-one (3u)[2]

To a 10 mL Schlenk tube equipped with a magnetic stir bar was added *N*-benzyl-2-bromo-5-methylbenzamide **1u** (152 mg, 0.500 mmol, 1.00 equiv), *o*-bromobenzoic acid **2a** (151 mg, 0.750 mmol, 1.50 equiv), DMF (4.0 mL), Cs_2CO_3 (163 mg, 0.500 mmol, 1.00 equiv), PPh_3 (26 mg, 0.100 mmol, 0.200 equiv), $Pd(OAc)_2$ (11 mg, 0.05 mmol, 0.100 equiv). The reaction mixture was stirred at 120 °C in an oil bath for about 10 h. The resulting mixture was concentrated and the residue was taken up in ethyl acetate. The organic layer was washed with brine, dried over Na_2SO_4 and concentrated. Purification of the crude product by column chromatography (silica gel; petroleum ether/ethyl acetate 30:1) afforded **3u** in 64% yield (96 mg).

White solid; mp 173–175 °C; ^{1}H NMR ($CDCl_3$, 400 MHz): δ_H 8.43 (s, 1H), 8.26 (d, *J* = 8.0 Hz, 1H), 8.20 (d, *J* = 8.4 Hz, 1H), 7.62 (d, *J* = 8.4 Hz, 1H), 7.39–7.35 (m, 1H), 7.31–7.21 (m, 7H), 5.67 (s, 2H), 2.55 (s, 3H); $^{13}C\{^1H\}$ NMR ($CDCl_3$, 101 MHz): δ_C 162.0, 138.2, 137.0, 136.6, 134.0, 131.4, 129.0, 128.9, 128.8, 127.1, 126.5, 125.2, 123.0, 122.5, 121.7, 119.7, 116.0, 46.4, 21.4; HRMS (ESI-TOF, *m/z*): calcd for $C_{21}H_{17}NNaO$ [M + Na]$^+$, 322.1202; found, 322.1199.

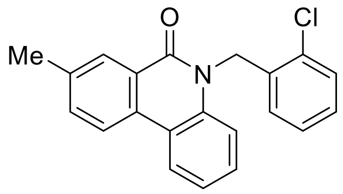

5-(2-Chlorobenzyl)-8-methylphenanthridin-6(5*H*)-one (3v)

To a 10 mL Schlenk tube equipped with a magnetic stir bar was added 2-bromo-*N*-(2-chlorobenzyl)-5-methylbenzamide **1v** (169 mg, 0.500 mmol, 1.00 equiv), *o*-bromobenzoic acid **2a** (151 mg, 0.750 mmol, 1.50 equiv), DMF (4.0 mL), Cs_2CO_3 (163 mg, 0.500 mmol, 1.00 equiv), PPh_3 (26 mg, 0.100 mmol, 0.200 equiv), $Pd(OAc)_2$ (11 mg, 0.05 mmol, 0.100 equiv). The reaction mixture was stirred at 120 °C in an oil bath for about 10 h. The resulting mixture was concentrated and the residue was taken up in ethyl acetate. The organic layer was washed with brine, dried over Na_2SO_4 and concentrated. Purification of the crude product by column chromatography (silica gel; petroleum ether/ethyl acetate 30:1) afforded **3v** in 72% yield (120 mg).

White solid; mp 144–145 °C; ^{1}H NMR ($CDCl_3$, 400 MHz): δ_H 8.42 (s, 1H), 8.28 (d, *J* = 8.0 Hz, 1H), 8.23 (d, *J* = 8.4 Hz, 1H), 7.64 (d, *J* = 8.0 Hz, 1H), 7.45 (d, *J* = 8.0 Hz, 1H), 7.38 (t, *J* = 7.2 Hz, 1H), 7.29 (d, *J* = 7.6 Hz, 1H), 7.18 (t, *J* = 7.6 Hz, 1H), 7.10–7.03 (m, 2H), 6.82 (d, *J* = 7.6 Hz, 1H), 5.73 (s, 2H), 2.55 (s, 3H); ^{13}C{^{1}H} NMR ($CDCl_3$, 101 MHz): δ_C 162.0, 138.3, 136.6, 134.2, 133.6, 132.6, 131.4, 129.6, 129.3, 128.9, 128.3, 127.2, 126.9, 125.1, 123.1, 122.7, 121.8, 119.7, 115.8, 44.3, 21.4; HRMS (ESI-TOF, *m/z*): calcd for $C_{21}H_{16}ClNNaO$ $[M + Na]^+$, 356.0813; found, 356.0822.

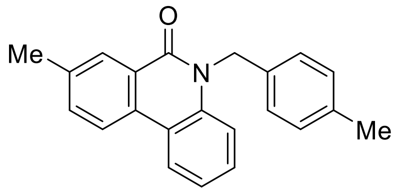

8-Methyl-5-(4-methylbenzyl)phenanthridin-6(5*H*)-one (3w)

To a 10 mL Schlenk tube equipped with a magnetic stir bar was added 2-bromo-5-methyl-*N*-(4-methylbenzyl)benzamide **1w** (159 mg, 0.500 mmol, 1.00 equiv), *o*-bromobenzoic acid **2a** (151 mg, 0.750 mmol, 1.50 equiv), DMF (4.0 mL), Cs_2CO_3 (163 mg, 0.500 mmol, 1.00 equiv), PPh_3 (26 mg, 0.100 mmol, 0.200 equiv), $Pd(OAc)_2$ (11 mg, 0.05 mmol, 0.100 equiv). The reaction mixture was stirred at 120 °C in an oil bath for about 10 h. The resulting mixture was concentrated and the residue was taken up in ethyl acetate. The organic layer was washed with brine, dried over Na_2SO_4 and concentrated.

Purification of the crude product by column chromatography (silica gel; petroleum ether/ethyl acetate 30:1) afforded **3w** in 62% yield (97 mg).

White solid; mp 140–141 °C; ^{1}H NMR (CDCl$_3$, 400 MHz): δ_H 8.42 (s, 1H), 8.26 (d, *J* = 8.0 Hz, 1H), 8.20 (d, *J* = 8.4 Hz, 1H), 7.61 (d, *J* = 8.4 Hz, 1H), 7.39–7.31 (m, 1H), 7.27–7.24 (m, 2H), 7.16 (d, *J* = 8.0 Hz, 2H), 7.09 (d, *J* = 8.4 Hz, 2H), 5.63 (s, 2H), 2.55 (s, 3H), 2.29 (s, 3H); ^{13}C{^{1}H} NMR (CDCl$_3$, 101 MHz): δ_C 162.0, 138.2, 137.0, 136.7, 134.0, 133.6, 131.4, 129.4, 129.0, 128.9, 126.5, 125.3, 123.0, 122.4, 121.7, 119.7, 116.0, 46.2, 21.4, 21.0; HRMS (ESI-TOF, *m/z*): calcd for $C_{22}H_{20}NO$ [M + H]$^+$, 314.1539; found, 314.1547.

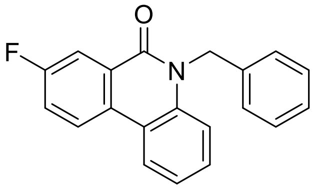

5-Benzyl-8-fluorophenanthridin-6(5*H*)-one (3x)[2]

To a 10 mL Schlenk tube equipped with a magnetic stir bar was added *N*-benzyl-2-bromo-5-fluorobenzamide **1x** (154 mg, 0.500 mmol, 1.00 equiv), *o*-bromobenzoic acid **2a** (151 mg, 0.750 mmol, 1.50 equiv), DMF (4.0 mL), Cs_2CO_3 (163 mg, 0.500 mmol, 1.00 equiv), PPh_3 (26 mg, 0.100 mmol, 0.200 equiv), $Pd(OAc)_2$ (11 mg, 0.05 mmol, 0.100 equiv). The reaction mixture was stirred at 120 °C in an oil bath for about 10 h. The resulting mixture was concentrated and the residue was taken up in ethyl acetate. The organic layer was washed with brine, dried over Na_2SO_4 and concentrated. Purification of the crude product by column chromatography (silica gel; petroleum ether/ethyl acetate 30:1) afforded **3x** in 70% yield (106 mg).

White solid; mp 164–166 °C; ^{1}H NMR (CDCl$_3$, 400 MHz): δ_H 8.31–8.25 (m, 2H), 8.21 (d, *J* = 8.0 Hz, 1H), 7.53–7.49 (m, 1H), 7.39 (t, *J* = 8.0 Hz, 1H), 7.33–7.22 (m, 7H), 5.66 (s, 2H); ^{13}C{^{1}H} NMR (CDCl$_3$, 101 MHz): δ_C 162.3 (d, $J_{C\text{–}F}$ = 247.0 Hz), 161.0 (d, $J_{C\text{–}F}$ = 2.0 Hz), 161.0, 136.8, 136.3, 130.3(d, $J_{C\text{–}F}$ = 2.0 Hz), 129.4, 128.8, 127.3(d, $J_{C\text{–}F}$ = 7.1 Hz), 127.27, 126.5, 124.3 (d, $J_{C\text{–}F}$ = 8.0 Hz), 123.1, 122.8, 121.1 (d, $J_{C\text{–}F}$ = 24.0 Hz), 119.0, 116.1, 114.5 (d, $J_{C\text{–}F}$ = 23.0 Hz), 46.6; HRMS (ESI-TOF, *m/z*): calcd for $C_{20}H_{15}FNO$ [M + H]$^+$, 304.1132; found, 304.1138.

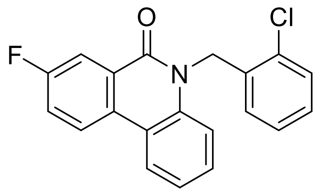

5-(2-Chlorobenzyl)-8-fluorophenanthridin-6(5*H*)-one (3y)

To a 10 mL Schlenk tube equipped with a magnetic stir bar was added 2-bromo-*N*-(2-chlorobenzyl)-5-fluorobenzamide **1y** (171 mg, 0.500 mmol, 1.00 equiv), *o*-bromobenzoic acid **2a** (151 mg, 0.750 mmol, 1.50 equiv), DMF (4.0 mL), Cs_2CO_3 (163 mg, 0.500 mmol, 1.00 equiv), PPh_3 (26 mg, 0.100 mmol, 0.200 equiv), $Pd(OAc)_2$ (11 mg, 0.05 mmol, 0.100 equiv). The reaction mixture was stirred at 120 °C in an oil bath for about 10 h. The resulting mixture was concentrated and the residue was taken up in ethyl acetate. The organic layer was washed with brine, dried over Na_2SO_4 and concentrated. Purification of the crude product by column chromatography (silica gel; petroleum ether/ethyl acetate 30:1) afforded **3y** in 79% yield (134 mg).

White solid; mp 143–144 °C; ^{1}H NMR ($CDCl_3$, 400 MHz): δ_H 8.32 (dd, *J* = 8.8, 4.8 Hz, 1H), 8.27–8.23 (m, 2H), 7.56–7.51 (m, 1H), 7.46 (d, *J* = 8.0 Hz, 1H), 7.41 (t, *J* = 7.6 Hz, 1H), 7.30 (t, *J* = 7.6 Hz, 1H), 7.19 (t, *J* = 7.6 Hz, 1H), 7.12–7.04 (m, 2H), 6.80 (d, *J* = 7.6 Hz, 1H), 5.71 (s, 2H); ^{13}C{^{1}H} NMR ($CDCl_3$, 101 MHz): δ_C 162.4 (d, J_{C-F} = 248.0 Hz), 161.0 (d, J_{C-F} = 3.0 Hz), 136.5, 133.2, 132.6, 130.3 (d, J_{C-F} = 1.0 Hz), 129.6, 128.5, 127.2, 127.16(d, J_{C-F} = 9.6 Hz), 126.8, 124.4 (d, J_{C-F} = 8.0 Hz), 123.2, 123.0, 121.2 (d, J_{C-F} = 23.0 Hz), 119.0, 115.9, 114.5 (d, J_{C-F} = 23.0 Hz), 44.5; HRMS (ESI-TOF, *m/z*): calcd for $C_{20}H_{14}ClFNO$ [M + H]$^+$, 338.0742; found, 338.0719.

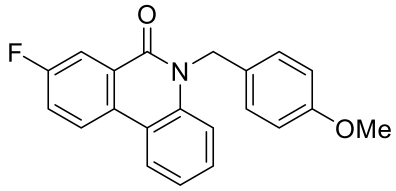

8-Fluoro-5-(4-methoxybenzyl)phenanthridin-6(5*H*)-one (3z)

To a 10 mL Schlenk tube equipped with a magnetic stir bar was added 2-bromo-5-fluoro-*N*-(4-methoxybenzyl)benzamide **1z** (169 mg, 0.500 mmol, 1.00 equiv), *o*-bromobenzoic acid **2a** (151 mg, 0.750 mmol, 1.50 equiv), DMF (4.0 mL), Cs_2CO_3 (163 mg, 0.500 mmol, 1.00 equiv), PPh_3 (26 mg, 0.100 mmol, 0.200 equiv), $Pd(OAc)_2$ (11 mg, 0.05 mmol, 0.100 equiv). The reaction mixture was stirred at 120 °C in an oil bath for about 10 h. The resulting mixture was concentrated and the residue was taken

up in ethyl acetate. The organic layer was washed with brine, dried over Na_2SO_4 and concentrated. Purification of the crude product by column chromatography (silica gel; petroleum ether/ethyl acetate 30:1) afforded **3z** in 72% yield (120 mg).

White solid; mp 130–131 °C; ^{1}H NMR ($CDCl_3$, 400 MHz): δ_H 8.31–8.20 (m, 3H), 7.51 (t, *J* = 8.8 Hz, 1H), 7.43–7.36 (m, 2H), 7.29 (d, *J* = 7.2 Hz, 1H), 7.20 (d, *J* = 8.0 Hz, 2H), 6.83 (d, *J* = 8.0 Hz, 2H), 5.59 (s, 2H), 3.75 (s, 3H); ^{13}C{^{1}H} NMR ($CDCl_3$, 101 MHz): δ_C 162.3 (d, $J_{C–F}$ = 247.0 Hz), 161.0 (d, $J_{C–F}$ = 3.0 Hz), 158.8, 136.8, 130.3 (d, $J_{C–F}$ = 2.0 Hz), 129.4, 128.3, 127.9, 127.3(d, $J_{C–F}$ = 7.8 Hz), 124.3 (d, $J_{C–F}$ = 8.0 Hz), 123.1, 122.8, 121.0 (d, $J_{C–F}$ = 23.0 Hz), 119.0, 116.1, 114.5 (d, $J_{C–F}$ = 23.0 Hz), 114.2, 55.2, 46.1; HRMS (ESI-TOF, *m/z*): calcd for $C_{21}H_{16}FNNaO_2$ [M + Na]$^+$, 356.1057; found, 356.1065.

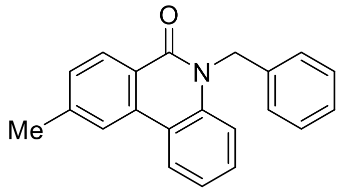

5-Benzyl-9-methylphenanthridin-6(5*H*)-one (3aa)[2]

To a 10 mL Schlenk tube equipped with a magnetic stir bar was added *N*-benzyl-2-bromo-4-methylbenzamide **1aa** (152 mg, 0.500 mmol, 1.00 equiv), *o*-bromobenzoic acid **2a** (151 mg, 0.750 mmol, 1.50 equiv), DMF (4.0 mL), Cs_2CO_3 (163 mg, 0.500 mmol, 1.00 equiv), PPh_3 (26 mg, 0.100 mmol, 0.200 equiv), $Pd(OAc)_2$ (11 mg, 0.05 mmol, 0.100 equiv). The reaction mixture was stirred at 120 °C in an oil bath for about 10 h. The resulting mixture was concentrated and the residue was taken up in ethyl acetate. The organic layer was washed with brine, dried over Na_2SO_4 and concentrated. Purification of the crude product by column chromatography (silica gel; petroleum ether/ethyl acetate 30:1) afforded **3aa** in 72% yield (107 mg).

White solid; mp 173–175 °C; ^{1}H NMR ($CDCl_3$, 400 MHz): δ_H 8.50 (d, *J* = 8.0 Hz, 1H), 8.28 (d, *J* = 8.0 Hz, 1H), 8.09 (s, 1H), 7.44 (d, *J* = 8.0 Hz, 1H), 7.38 (t, *J* = 8.0 Hz, 1H), 7.30–7.20 (m, 7H), 5.66 (s, 2H), 2.58 (s, 3H); ^{13}C{^{1}H} NMR ($CDCl_3$, 101 MHz): δ_C 161.9, 143.2, 137.5, 136.7, 133.8, 129.5, 129.4, 129.2, 128.8, 127.1, 126.5, 123.2, 123.1, 122.4, 121.7, 119.5, 116.0, 46.4, 22.2; HRMS (ESI-TOF, *m/z*): calcd for $C_{21}H_{17}NNaO$ [M + Na]$^+$, 322.1202; found, 322.1199.

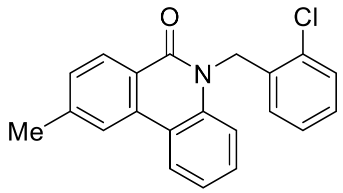

5-(2-Chlorobenzyl)-9-methylphenanthridin-6(5*H*)-one (3ab)

To a 10 mL Schlenk tube equipped with a magnetic stir bar was added 2-bromo-*N*-(2-chlorobenzyl)-4-methylbenzamide **1ab** (169 mg, 0.500 mmol, 1.00 equiv), *o*-bromobenzoic acid **2a** (151 mg, 0.750 mmol, 1.50 equiv), DMF (4.0 mL), Cs_2CO_3 (163 mg, 0.500 mmol, 1.00 equiv), PPh_3 (26 mg, 0.100 mmol, 0.200 equiv), $Pd(OAc)_2$ (11 mg, 0.05 mmol, 0.100 equiv). The reaction mixture was stirred at 120 °C in an oil bath for about 10 h. The resulting mixture was concentrated and the residue was taken up in ethyl acetate. The organic layer was washed with brine, dried over Na_2SO_4 and concentrated. Purification of the crude product by column chromatography (silica gel; petroleum ether/ethyl acetate 30:1) afforded **3ab** in 76% yield (127 mg).

White solid; mp 141–142 °C; ^{1}H NMR ($CDCl_3$, 400 MHz): δ_H 8.49 (d, *J* = 8.0 Hz, 1H), 8.31 (d, *J* = 8.0 Hz, 1H), 8.12 (s, 1H), 7.45 (d, *J* = 8.4 Hz, 2H), 7.39 (t, *J* = 7.6 Hz, 1H), 7.29 (d, *J* = 7.6 Hz, 1H), 7.18 (t, *J* = 7.6 Hz, 1H), 7.10–7.03 (m, 2H), 6.82 (d, *J* = 7.6 Hz, 1H), 5.71 (s, 2H), 2.60 (s, 3H); ^{13}C{^{1}H} NMR ($CDCl_3$, 101 MHz): δ_C 161.9, 143.4, 137.2, 133.8, 133.6, 132.6, 129.6, 129.6, 129.2, 128.3, 127.2, 127.0, 123.3, 123.0, 122.6, 121.8, 119.5, 115.8, 44.2, 22.2; HRMS (ESI-TOF, *m/z*): calcd for $C_{21}H_{16}ClNNaO$ $[M + Na]^+$, 356.0813; found, 356.0822.

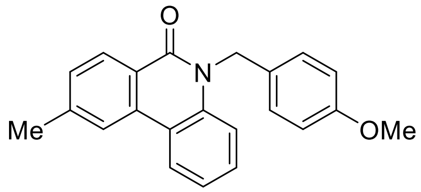

5-(4-Methoxybenzyl)-9-methylphenanthridin-6(5*H*)-one (3ac)

To a 10 mL Schlenk tube equipped with a magnetic stir bar was added 2-bromo-*N*-(4-methoxybenzyl)-4-methylbenzamide **1ac** (167 mg, 0.500 mmol, 1.00 equiv), *o*-bromobenzoic acid **2a** (151 mg, 0.750 mmol, 1.50 equiv), DMF (4.0 mL), Cs_2CO_3 (163 mg, 0.500 mmol, 1.00 equiv), PPh_3 (26 mg, 0.100 mmol, 0.200 equiv), $Pd(OAc)_2$ (11 mg, 0.05 mmol, 0.100 equiv). The reaction mixture was stirred at 120 °C in an oil bath for about 10 h. The resulting mixture was concentrated and the residue was taken up in ethyl acetate. The organic layer was washed with brine, dried over Na_2SO_4 and concentrated.

Purification of the crude product by column chromatography (silica gel; petroleum ether/ethyl acetate 30:1) afforded **3ac** in 69% yield (113 mg).

White solid; mp 145–146 °C; ^{1}H NMR ($CDCl_3$, 400 MHz): δ_H 8.50 (d, *J* = 8.0 Hz, 1H), 8.28 (d, *J* = 8.0 Hz, 1H), 8.09 (s, 1H), 7.43 (d, *J* = 8.0 Hz, 1H), 7.38 (d, *J* = 7.2 Hz, 1H), 7.33 (d, *J* = 8.4 Hz, 1H), 7.27–7.23 (m, 1H), 7.20 (d, *J* = 8.0 Hz, 2H), 6.82 (d, *J* = 7.6 Hz, 2H), 5.59 (s, 2H), 3.75 (s, 3H), 2.58 (s, 3H); ^{13}C{^{1}H} NMR ($CDCl_3$, 101 MHz): δ_C 161.9, 158.7, 143.2, 137.5, 133.8, 129.4, 129.4, 129.1, 128.8, 127.8, 123.2, 122.4, 121.7, 119.5, 116.0, 114.2, 55.2, 45.8, 22.2; HRMS (ESI-TOF, *m/z*): calcd for $C_{22}H_{19}NNaO_2$ [M + Na]$^+$, 352.1308; found, 352.1312.

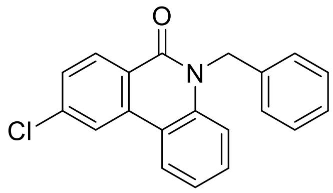

5-Benzyl-9-chlorophenanthridin-6(5*H*)-one (3ad)

To a 10 mL Schlenk tube equipped with a magnetic stir bar was added *N*-benzyl-2-bromo-4-chlorobenzamide **1ad** (162 mg, 0.500 mmol, 1.00 equiv), *o*-bromobenzoic acid **2a** (151 mg, 0.750 mmol, 1.50 equiv), DMF (4.0 mL), Cs_2CO_3 (163 mg, 0.500 mmol, 1.00 equiv), PPh_3 (26 mg, 0.100 mmol, 0.200 equiv), $Pd(OAc)_2$ (11 mg, 0.05 mmol, 0.100 equiv). The reaction mixture was stirred at 120 °C in an oil bath for about 10 h. The resulting mixture was concentrated and the residue was taken up in ethyl acetate. The organic layer was washed with brine, dried over Na_2SO_4 and concentrated. Purification of the crude product by column chromatography (silica gel; petroleum ether/ethyl acetate 30:1) afforded **3ad** in 70% yield (112 mg).

White solid; mp 139–140 °C; ^{1}H NMR ($CDCl_3$, 400 MHz): δ_H 8.52 (d, *J* = 8.4 Hz, 1H), 8.24 (s, 1H), 8.19 (d, *J* = 8.0 Hz, 1H), 7.56 (d, *J* = 8.8 Hz, 1H), 7.42 (t, *J* = 7.69 Hz, 1H), 7.31–7.21 (m, 7H), 5.64 (s, 2H); ^{13}C{^{1}H} NMR ($CDCl_3$, 101 MHz): δ_C 161.2, 139.5, 137.7, 136.3, 135.3, 131.0, 130.3, 128.8, 128.4, 127.3, 126.5, 123.7, 123.4, 122.7, 121.6, 118.4, 116.1, 46.3; HRMS (ESI-TOF, *m/z*): calcd for $C_{20}H_{15}ClNO$ [M + H]$^+$, 320.0837; found, 320.0822.

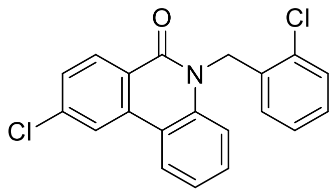

9-Chloro-5-(2-chlorobenzyl)phenanthridin-6(5*H*)-one (3ae)

To a 10 mL Schlenk tube equipped with a magnetic stir bar was added 2-bromo-4-chloro-*N*-(2-chlorobenzyl)benzamide **1ae** (180 mg, 0.500 mmol, 1.00 equiv), *o*-bromobenzoic acid **2a** (151 mg, 0.750 mmol, 1.50 equiv), DMF (4.0 mL), Cs_2CO_3 (163 mg, 0.500 mmol, 1.00 equiv), PPh_3 (26 mg, 0.100 mmol, 0.200 equiv), $Pd(OAc)_2$ (11 mg, 0.05 mmol, 0.100 equiv). The reaction mixture was stirred at 120 °C in an oil bath for about 10 h. The resulting mixture was concentrated and the residue was taken up in ethyl acetate. The organic layer was washed with brine, dried over Na_2SO_4 and concentrated. Purification of the crude product by column chromatography (silica gel; petroleum ether/ethyl acetate 30:1) afforded **3ae** in 78% yield (138 mg).

White solid; mp 145–146 °C; ^{1}H NMR ($CDCl_3$, 400 MHz): δ_H 8.52 (d, *J* = 8.4 Hz, 1H), 8.25 (s, 1H), 8.19 (d, *J* = 8.0 Hz, 1H), 7.56 (d, *J* = 8.4 Hz, 1H), 7.46–7.40 (m, 2H), 7.31–7.26 (m, 1H), 7.18 (t, *J* = 7.6 Hz, 1H), 7.10–7.04 (m, 2H), 6.80 (d, *J* = 8.0 Hz, 1H), 5.67 (s, 2H); ^{13}C{^{1}H} NMR ($CDCl_3$, 101 MHz): δ_C 161.2, 139.6, 137.4, 135.3, 133.2, 132.5, 130.9, 130.5, 129.6, 128.5, 128.5, 127.2, 126.8, 123.6, 123.4, 123.0, 121.7, 118.4, 115.9, 44.3; HRMS (ESI-TOF, *m/z*): calcd for $C_{20}H_{14}Cl_2NO$ [M + H]$^+$, 354.0447; found, 354.0424.

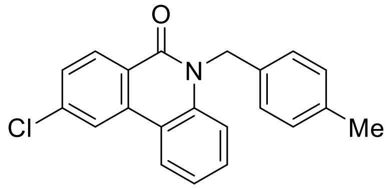

9-Chloro-5-(4-methylbenzyl)phenanthridin-6(5*H*)-one (3af)

To a 10 mL Schlenk tube equipped with a magnetic stir bar was added 2-bromo-4-chloro-*N*-(4-methylbenzyl)benzamide **1af** (169 mg, 0.500 mmol, 1.00 equiv), *o*-bromobenzoic acid **2a** (151 mg, 0.750 mmol, 1.50 equiv), DMF (4.0 mL), Cs_2CO_3 (163 mg, 0.500 mmol, 1.00 equiv), PPh_3 (26 mg, 0.100 mmol, 0.200 equiv), $Pd(OAc)_2$ (11 mg, 0.05 mmol, 0.100 equiv). The reaction mixture was stirred at 120 °C in an oil bath for about 10 h. The resulting mixture was concentrated and the residue was taken up in ethyl acetate. The organic layer was washed with brine, dried over Na_2SO_4 and concentrated.

Purification of the crude product by column chromatography (silica gel; petroleum ether/ethyl acetate 30:1) afforded **3af** in 67% yield (112 mg).

White solid; mp 149–150 °C; ^{1}H NMR ($CDCl_3$, 400 MHz): δ_H 8.54 (d, *J* = 8.4 Hz, 1H), 8.24 (s, 1H), 8.18 (d, *J* = 7.6 Hz, 1H), 7.55 (d, *J* = 8.4 Hz, 1H), 7.42 (t, *J* = 8.0 Hz, 1H), 7.33 (d, *J* = 8.8 Hz, 1H), 7.28–7.25 (m, 1H), 7.15 (d, *J* = 7.6 Hz, 2H), 7.10 (d, *J* = 8.0 Hz, 2H), 5.59 (s, 2H), 2.29 (s, 3H); ^{13}C{^{1}H} NMR ($CDCl_3$, 101 MHz): δ_C 161.2, 139.4, 137.8, 136.9, 135.3, 133.3, 131.0, 130.2, 129.5, 128.4, 126.5, 123.8, 123.4, 122.7, 121.6, 118.4, 116.2, 46.3, 21.0; HRMS (ESI-TOF, *m/z*): calcd for $C_{21}H_{16}ClNNaO$ [M + Na]$^+$, 356.0813; found, 356.0822.

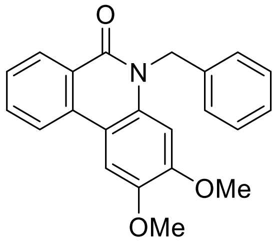

5-Benzyl-2,3-dimethoxyphenanthridin-6(5*H*)-one (3ag)[2]

To a 10 mL Schlenk tube equipped with a magnetic stir bar was added *N*-benzyl-2-bromobenzamide **1j** (145 mg, 0.500 mmol, 1.00 equiv), 2-bromo-4,5-dimethoxybenzoic acid **2b** (196 mg, 0.750 mmol, 1.50 equiv), DMF (4.0 mL), Cs_2CO_3 (163 mg, 0.500 mmol, 1.00 equiv), PPh_3 (26 mg, 0.100 mmol, 0.200 equiv), $Pd(OAc)_2$ (11 mg, 0.05 mmol, 0.100 equiv). The reaction mixture was stirred at 120 °C in an oil bath for about 10 h. The resulting mixture was concentrated and the residue was taken up in ethyl acetate. The organic layer was washed with brine, dried over Na_2SO_4 and concentrated. Purification of the crude product by column chromatography (silica gel; petroleum ether/ethyl acetate 30:1) afforded **3ag** in 84% yield (145 mg).

White solid; mp 153–155 °C; ^{1}H NMR ($CDCl_3$, 400 MHz): δ_H 8.61 (d, *J* = 8.0 Hz, 1H), 8.14 (d, *J* = 8.4 Hz, 1H), 7.77 (t, *J* = 8.0 Hz, 1H), 7.65 (s, 1H), 7.56 (t, *J* = 7.6 Hz, 1H), 7.32–7.23 (m, 5H), 6.80 (s, 1H), 5.67 (s, 2H), 3.99 (s, 3H), 3.75 (s, 3H); ^{13}C{^{1}H} NMR ($CDCl_3$, 101 MHz): δ_C 161.9, 150.6, 145.1, 136.9, 133.8, 132.5, 132.2, 129.3, 128.9, 127.3, 126.9, 126.6, 124.5, 121.1, 112.2, 105.2, 99.9, 56.3, 55.8, 46.8; HRMS (ESI-TOF, *m/z*): calcd for $C_{22}H_{20}NO_3$ [M + H]$^+$, 346.1438; found, 346.1442.

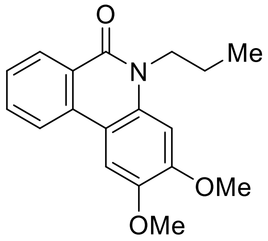

2,3-Dimethoxy-5-propylphenanthridin-6(5*H*)-one (3ah)

To a 10 mL Schlenk tube equipped with a magnetic stir bar was added 2-bromo-*N*-propylbenzamide **1c** (121 mg, 0.500 mmol, 1.00 equiv), 2-bromo-4,5-dimethoxybenzoic acid **2b** (196 mg, 0.750 mmol, 1.50 equiv), DMF (4.0 mL), Cs_2CO_3 (163 mg, 0.500 mmol, 1.00 equiv), PPh_3 (26 mg, 0.100 mmol, 0.200 equiv), $Pd(OAc)_2$ (11 mg, 0.05 mmol, 0.100 equiv). The reaction mixture was stirred at 120 °C in an oil bath for about 10 h. The resulting mixture was concentrated and the residue was taken up in ethyl acetate. The organic layer was washed with brine, dried over Na_2SO_4 and concentrated. Purification of the crude product by column chromatography (silica gel; petroleum ether/ethyl acetate 30:1) afforded **3ah** in 80% yield (119 mg).

White solid; mp 118–119 °C; ^{1}H NMR ($CDCl_3$, 400 MHz): δ_H 8.53 (d, *J* = 8.0 Hz, 1H), 8.10 (d, *J* = 8.4 Hz, 1H), 7.72 (t, *J* = 7.2 Hz, 1H), 7.67–7.66 (m, 1H), 7.52 (t, *J* = 7.2 Hz, 1H), 6.86 (s, 1H), 4.35 (t, *J* = 6.4 Hz, 2H), 4.02 (s, 3H), 4.01 (s, 3H), 1.91–1.82 (m, 2H), 1.09 (t, *J* = 7.2 Hz, 3H); ^{13}C{^{1}H} NMR ($CDCl_3$, 101 MHz): δ_C 161.3, 150.8, 145.0, 133.5, 132.2, 132.1, 129.0, 126.8, 124.7, 121.0, 112.2, 105.5, 98.8, 56.3, 56.0, 44.4, 20.8, 11.5; HRMS (ESI-TOF, *m/z*): calcd for $C_{18}H_{20}NO_3$ $[M + H]^+$, 298.1438; found, 298.1443.

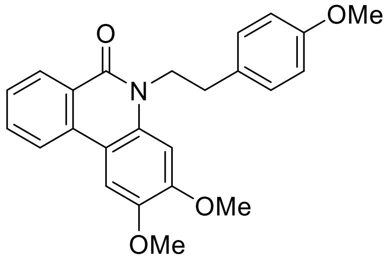

2,3-Dimethoxy-5-(4-methoxyphenethyl)phenanthridin-6(5*H*)-one (3ai)

To a 10 mL Schlenk tube equipped with a magnetic stir bar was added 2-bromo-*N*-(4-methoxyphenethyl)benzamide **1q** (167 mg, 0.500 mmol, 1.00 equiv), 2-bromo-4,5-dimethoxybenzoic acid **2b** (196 mg, 0.750 mmol, 1.50 equiv), DMF (4.0 mL), Cs_2CO_3 (163 mg, 0.500 mmol, 1.00 equiv), PPh_3 (26 mg, 0.100 mmol, 0.200 equiv), $Pd(OAc)_2$ (11 mg, 0.05 mmol, 0.100 equiv). The reaction mixture was stirred at 120 °C in an oil bath for about 10 h. The resulting mixture was concentrated

and the residue was taken up in ethyl acetate. The organic layer was washed with brine, dried over Na_2SO_4 and concentrated. Purification of the crude product by column chromatography (silica gel; petroleum ether/ethyl acetate 30:1) afforded **3ai** in 88% yield (171 mg).

White solid; mp 185–186 °C; ^{1}H NMR (CDCl$_3$, 400 MHz): δ_H 8.55 (d, *J* = 8.0 Hz, 1H), 8.14 (d, *J* = 8.0 Hz, 1H), 7.75 (t, *J* = 7.6 Hz, 1H), 7.69 (s, 1H), 7.55 (t, *J* = 7.2 Hz, 1H), 7.27 (d, *J* = 6.4 Hz, 2H), 6.88 (d, *J* = 6.4 Hz, 3H), 4.55 (t, *J* = 8.0 Hz, 2H), 4.04 (s, 3H), 3.98 (s, 3H), 3.80 (s, 3H), 3.07 (t, *J* = 8.0 Hz, 2H); ^{13}C{^{1}H} NMR (CDCl$_3$, 101 MHz): δ_C 161.3, 158.4, 150.8, 145.1, 133.5, 132.3, 132.0, 130.7, 129.7, 128.9, 126.9, 124.7, 121.1, 114.2, 112.2, 105.4, 98.6, 56.3, 56.0, 55.3, 45.0, 33.0; HRMS (ESI-TOF, *m/z*): calcd for $C_{24}H_{24}NO_4$ [M + H]$^+$, 390.1700; found, 390.1713.

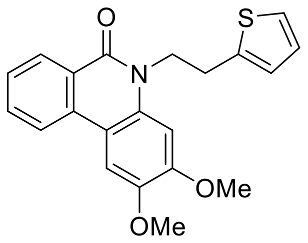

2,3-Dimethoxy-5-(4-methoxyphenethyl)phenanthridin-6(5*H*)-one (3aj)

To a 10 mL Schlenk tube equipped with a magnetic stir bar was added 2-bromo-*N*-(2-(thiophen-2-yl)ethyl)benzamide **1s** (155 mg, 0.500 mmol, 1.00 equiv), 2-bromo-4,5-dimethoxybenzoic acid **2b** (196 mg, 0.750 mmol, 1.50 equiv), DMF (4.0 mL), Cs_2CO_3 (163 mg, 0.500 mmol, 1.00 equiv), PPh_3 (26 mg, 0.100 mmol, 0.200 equiv), $Pd(OAc)_2$ (11 mg, 0.05 mmol, 0.100 equiv). The reaction mixture was stirred at 120 °C in an oil bath for about 10 h. The resulting mixture was concentrated and the residue was taken up in ethyl acetate. The organic layer was washed with brine, dried over Na_2SO_4 and concentrated. Purification of the crude product by column chromatography (silica gel; petroleum ether/ethyl acetate 30:1) afforded **3aj** in 83% yield (151 mg).

White solid; mp 143–144 °C; ^{1}H NMR (CDCl$_3$, 400 MHz): δ_H 8.55 (d, *J* = 8.0 Hz, 1H), 8.14 (d, *J* = 8.0 Hz, 1H), 7.74 (t, *J* = 7.6 Hz, 1H), 7.66 (s, 1H), 7.54 (t, *J* = 7.2 Hz, 1H), 7.18 (d, *J* = 5.2 Hz, 1H), 6.97–6.94 (m, 2H), 6.82 (s, 1H), 4.60 (t, *J* = 7.6 Hz, 2H), 4.02 (s, 3H), 3.98 (s, 3H), 3.34 (t, *J* = 7.6 Hz, 2H); ^{13}C{^{1}H} NMR (CDCl$_3$, 101 MHz): δ_C 161.3, 150.9, 145.1, 140.6, 133.6, 132.4, 131.9, 128.9, 127.3, 126.9, 125.6, 124.5, 124.0, 121.1, 112.1, 105.5, 98.4, 56.3, 56.1, 44.9, 27.8; HRMS (ESI-TOF, *m/z*): calcd for $C_{21}H_{19}NNaO_3S$ [M + Na]$^+$, 388.0978; found, 388.0987.

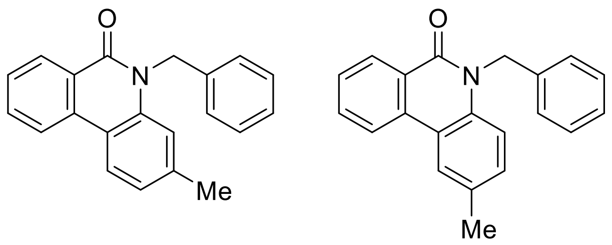

5-Benzyl-3-methylphenanthridin-6(5*H*)-one & 5-Benzyl-2-methylphenanthridin-6(5*H*)-one (**3ak, 3ak'**)

From 2-bromo-4-methylbenzoic acid: To a 10 mL Schlenk tube equipped with a magnetic stir bar was added *N*-benzyl-2-bromobenzamide **1k** (145 mg, 0.500 mmol, 1.00 equiv), 2-bromo-4-methylbenzoic acid **2c** (161 mg, 0.750 mmol, 1.50 equiv), DMF (4.0 mL), Cs_2CO_3 (163 mg, 0.500 mmol, 1.00 equiv), PPh_3 (26 mg, 0.100 mmol, 0.200 equiv), $Pd(OAc)_2$ (11 mg, 0.05 mmol, 0.100 equiv). The reaction mixture was stirred at 120 °C in an oil bath for about 10 h. The resulting mixture was concentrated and the residue was taken up in ethyl acetate. The organic layer was washed with brine, dried over Na_2SO_4 and concentrated. Purification of the crude product by column chromatography (silica gel; petroleum ether/ethyl acetate 30:1) afforded **3ak** and **3ak'** in 76% yield (113 mg).

White solid (mixture); ^{1}H NMR ($CDCl_3$, 400 MHz): δ_H 8.63–8.58 (m, 1.2H), 8.31–8.25 (m, 1.2H), 8.17-8.08 (m, 1.2H), 7.80–7.75 (m, 1.2H), 7.63–7.56 (m, 1.2H), 7.32–7.20 (m, 6H), 7.12–7.08 (m, 2.4H), 5.65 (s, 2.4H), 2.44 (m, 0.6H), 2.37 (m, 3H); $^{13}C\{^1H\}$ NMR ($CDCl_3$, 101 MHz): δ_C 162.0, 159.3, 139.8, 137.5, 137.4, 136.7, 134.0, 132.6, 132.55, 130.6, 129.2, 129.1, 128.8, 127.9, 127.5, 127.1, 126.5, 126.48, 125.0, 123.8, 123.4, 123.2, 121.6, 121.4, 117.1, 116.4, 116.2, 115.9, 46.4, 21.9, 20.9; HRMS (ESI-TOF, *m/z*): calcd for $C_{21}H_{17}NNaO$ $[M + Na]^+$, 322.1202; found, 322.1199.

From 2-bromo-5-methylbenzoic acid: To a 10 mL Schlenk tube equipped with a magnetic stir bar was added *N*-benzyl-2-bromobenzamide **1k** (145 mg, 0.500 mmol, 1.00 equiv), 2-bromo-4-methylbenzoic acid **2d** (161 mg, 0.750 mmol, 1.50 equiv), DMF (4.0 mL), Cs_2CO_3 (163 mg, 0.500 mmol, 1.00 equiv), PPh_3 (26 mg, 0.100 mmol, 0.200 equiv), $Pd(OAc)_2$ (11 mg, 0.05 mmol, 0.100 equiv). The reaction mixture was stirred at 120 °C in an oil bath for about 10 h. The resulting mixture was concentrated and the residue was taken up in ethyl acetate. The organic layer was washed with brine, dried over Na_2SO_4 and concentrated. Purification of the crude product by column chromatography (silica gel; petroleum ether/ethyl acetate 30:1) afforded **3ak** and **3ak'** in 59% yield (87 mg).

White solid (mixture); ^{1}H NMR ($CDCl_3$, 400 MHz): δ_H 8.62–8.58 (m, 2H), 8.30–8.24 (m, 2H), 8.16–8.07 (m, 2H), 7.77–7.74 (m, 2H), 7.62–7.57 (m, 2H), 7.30–7.27 (m, 6H), 7.23–7.19 (m, 6H), 7.11–7.07 (m, 2H), 5.64 (s, 4H), 2.43 (m, 3H), 2.36 (m, 3H); ^{13}C{^{1}H} NMR ($CDCl_3$, 101 MHz): δ_C 162.0, 161.7, 139.8, 137.4, 136.78, 136.7, 135.2, 133.9, 133.7, 132.6, 132.53, 130.5, 129.1, 128.7, 127.9, 127.5, 127.1, 126.5, 126.47, 125.5, 125.0, 123.8, 123.4, 123.1, 121.6, 121.4, 119.3, 117.0, 116.2, 115.9, 46.4, 21.9, 20.9; HRMS (ESI-TOF, *m/z*): calcd for $C_{21}H_{17}NNaO$ [M + Na]$^+$, 322.1202; found, 322.1199.

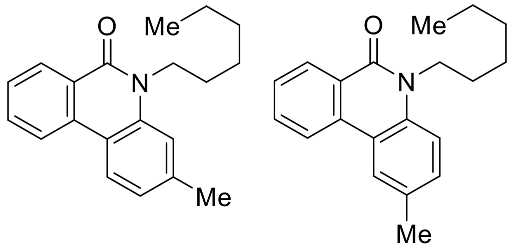

5-Hexyl-3-methylphenanthridin-6(5*H*)-one & 5-Hexyl-2-methylphenanthridin-6(5*H*)-one (3al, 3al')

From 2-bromo-4-methylbenzoic acid: To a 10 mL Schlenk tube equipped with a magnetic stir bar was added 2-bromo-*N*-cyclohexylbenzamide **1h** (141 mg, 0.500 mmol, 1.00 equiv), 2-bromo-4-methylbenzoic acid **2c** (161 mg, 0.750 mmol, 1.50 equiv), DMF (4.0 mL), Cs_2CO_3 (163 mg, 0.500 mmol, 1.00 equiv), PPh_3 (26 mg, 0.100 mmol, 0.200 equiv), $Pd(OAc)_2$ (11 mg, 0.05 mmol, 0.100 equiv). The reaction mixture was stirred at 120 °C in an oil bath for about 10 h. The resulting mixture was concentrated and the residue was taken up in ethyl acetate. The organic layer was washed with brine, dried over Na_2SO_4 and concentrated. Purification of the crude product by column chromatography (silica gel; petroleum ether/ethyl acetate 30:1) afforded **3al** and **3al'** in 65% yield (94 mg).

White oil; ^{1}H NMR ($CDCl_3$, 400 MHz): δ_H 8.56–8.52 (m, 1.2H), 8.28–8.21 (m, 1.2H), 8.17–8.08 (m, 1.2H), 7.76–7.70 (m, 1.2H), 7.59–7.52 (m, 1.2H), 7.36–7.26 (m, 1.2H), 7.18–7.11 (m, 1.2H), 4.38–4.34 (m, 2.4H), 2.52 (m, 0.6H), 2.48 (m, 3H), 1.80–1.78 (m, 2.4H), 1.53–1.48 (m, 2.4H), 1.37–1.34 (m, 2.4H), 0.93–0.89 (m, 3.6H); ^{13}C{^{1}H} NMR ($CDCl_3$, 101 MHz): δ_C 161.5, 161.2, 139.8, 137.2, 135.0, 133.7, 133.5, 132.3, 132.2, 131.6, 130.5, 128.8, 128.8, 127.8, 127.4, 125.7, 125.2, 123.6, 123.4, 123.3, 121.5, 121.3, 119.3, 117.1, 115.4, 115.0, 42.8, 42.7, 31.6, 31.6, 27.4, 27.4, 26.8, 22.6, 22.1, 20.9, 14.1; HRMS (ESI-TOF, *m/z*): calcd for $C_{20}H_{24}NO$ [M + H]$^+$, 294.1852; found, 294.1863.

From 2-bromo-5-methylbenzoic acid: To a 10 mL Schlenk tube equipped with a magnetic stir bar was added 2-bromo-*N*-cyclohexylbenzamide **1h** (141 mg, 0.500 mmol, 1.00 equiv), 2-bromo-5-methylbenzoic acid **2d** (161 mg, 0.750 mmol, 1.50 equiv), DMF (4.0 mL), Cs_2CO_3 (163 mg, 0.500 mmol, 1.00 equiv), PPh_3 (26 mg, 0.100 mmol, 0.200 equiv), $Pd(OAc)_2$ (11 mg, 0.05 mmol, 0.100 equiv). The reaction mixture was stirred at 120 °C in an oil bath for about 10 h. The resulting mixture was concentrated and the residue was taken up in ethyl acetate. The organic layer was washed with brine, dried over Na_2SO_4 and concentrated. Purification of the crude product by column chromatography (silica gel; petroleum ether/ethyl acetate 30:1) afforded **3al** and **3al'** in 60% yield (87 mg).

White oil; ^{1}H NMR ($CDCl_3$, 400 MHz): δ_H 8.56–8.52 (m, 1.4H), 8.28–8.21 (m, 1.4H), 8.17–8.08 (m, 1.4H), 7.76–7.70 (m, 1.4H), 7.59–7.52 (m, 1.4H), 7.36–7.26 (m, 1.4H), 7.29–7.12 (m, 1.4H), 4.38–4.34 (m, 2.8H), 2.52 (m, 1.2H), 2.48 (m, 3H), 1.80–1.78 (m, 2.8H), 1.52–1.48 (m, 2.8H), 1.38–1.36 (m, 5.6H), 0.92–0.89 (m, 4.2H); ^{13}C{^{1}H} NMR ($CDCl_3$, 101 MHz): δ_C 161.5, 161.2, 139.8, 137.2, 135.0, 133.7, 133.5, 132.3, 132.2, 131.6, 130.5, 128.8, 128.8, 127.8, 127.4, 125.7, 125.2, 123.6, 123.4, 123.3, 121.5, 121.3, 119.3, 117.1, 115.4, 115.0, 42.8, 42.7, 31.6, 31.6, 27.4, 27.4, 26.8, 22.6, 22.1, 20.9, 14.1; HRMS (ESI-TOF, *m/z*): calcd for $C_{20}H_{24}NO$ $[M + H]^+$, 294.1852; found, 294.1863.

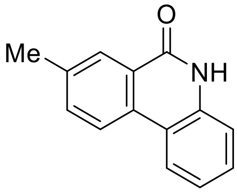

8-Methylphenanthridin-6(5*H*)-one (4)[4]

To a 10 mL Schlenk tube equipped with a magnetic stir bar was added 5-benzyl-8-methylphenanthridin-6(5*H*)-one **3u** (75 mg, 0.250 mmol), trifluoromethanesulfonic acid (4.0 mL). The resulting mixture was heated to reflux while being stirred and monitored by TLC until the starting materials were consumed (about 10 h). The mixture was neutralized by addition of a 10% sodium hydroxide solution, then extracted with ethyl acetate (20.0 mL × 2). The organic layer was washed with brine, dried over Na_2SO_4 and concentrated. Purification of the crude product by column chromatography (silica gel; petroleum ether/ethyl acetate 20:1) afforded **4** in 92% yield (48 mg).

White solid; mp 220–222 °C; ^{1}H NMR ($CDCl_3$, 400 MHz): δ_H 10.80 (s, 1H), 8.39 (s, 1H), 8.20 (d, *J* = 8.0 Hz, 2H), 7.63 (d, *J* = 8.0 Hz, 1H), 7.50–7.46 (m, 1H), 7.38 (d, *J* = 8.0 Hz, 1H), 7.31–7.26 (m, 1H), 2.56 (s,

3H); $^{13}C\{^1H\}$ NMR (DMSO-d_6, 101 MHz): δ_C 160.8, 137.6, 136.2, 133.9, 131.8, 129.1, 127.2, 125.6, 123.0, 122.6, 122.2, 117.7, 116.0, 20.9; HRMS (ESI-TOF, *m/z*): calcd for $C_{14}H_{12}NO$ $[M + H]^+$, 210.0913; found, 210.0916.

IV. NMR spectra

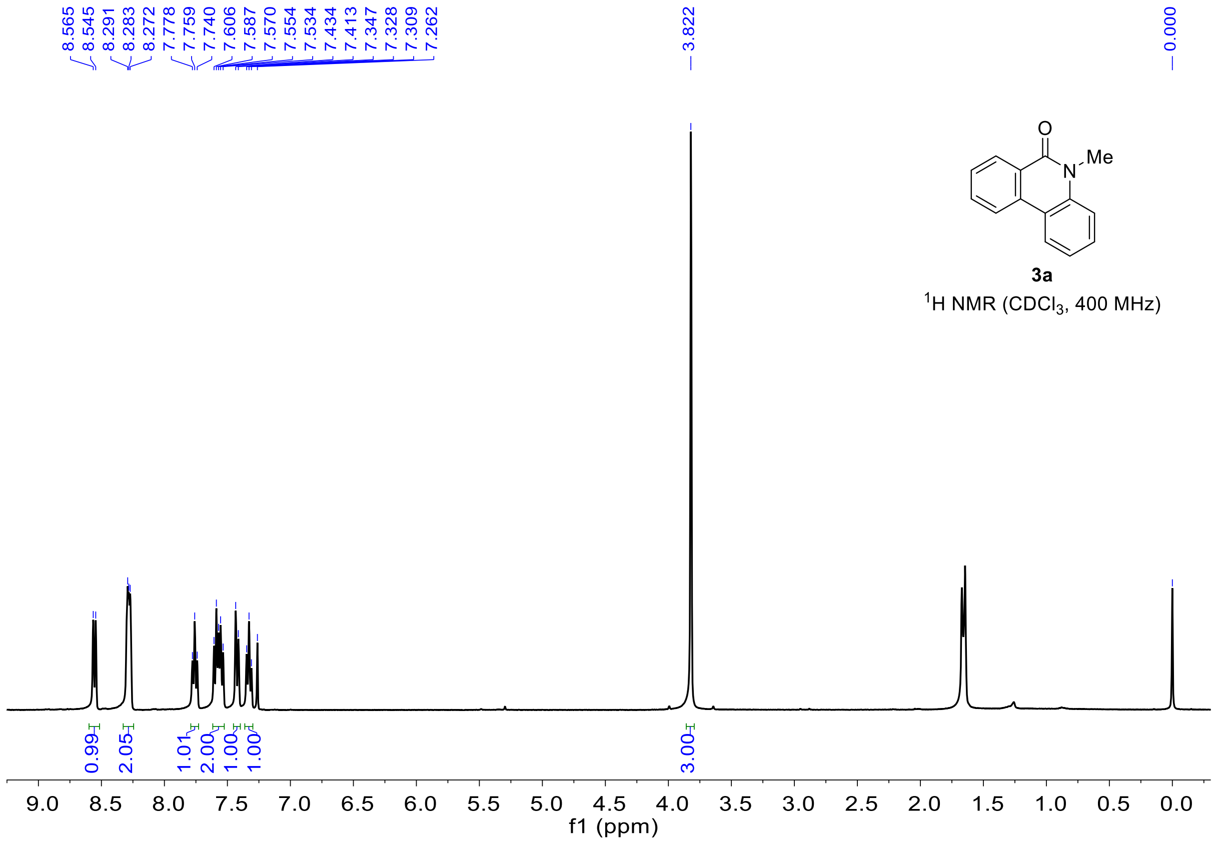

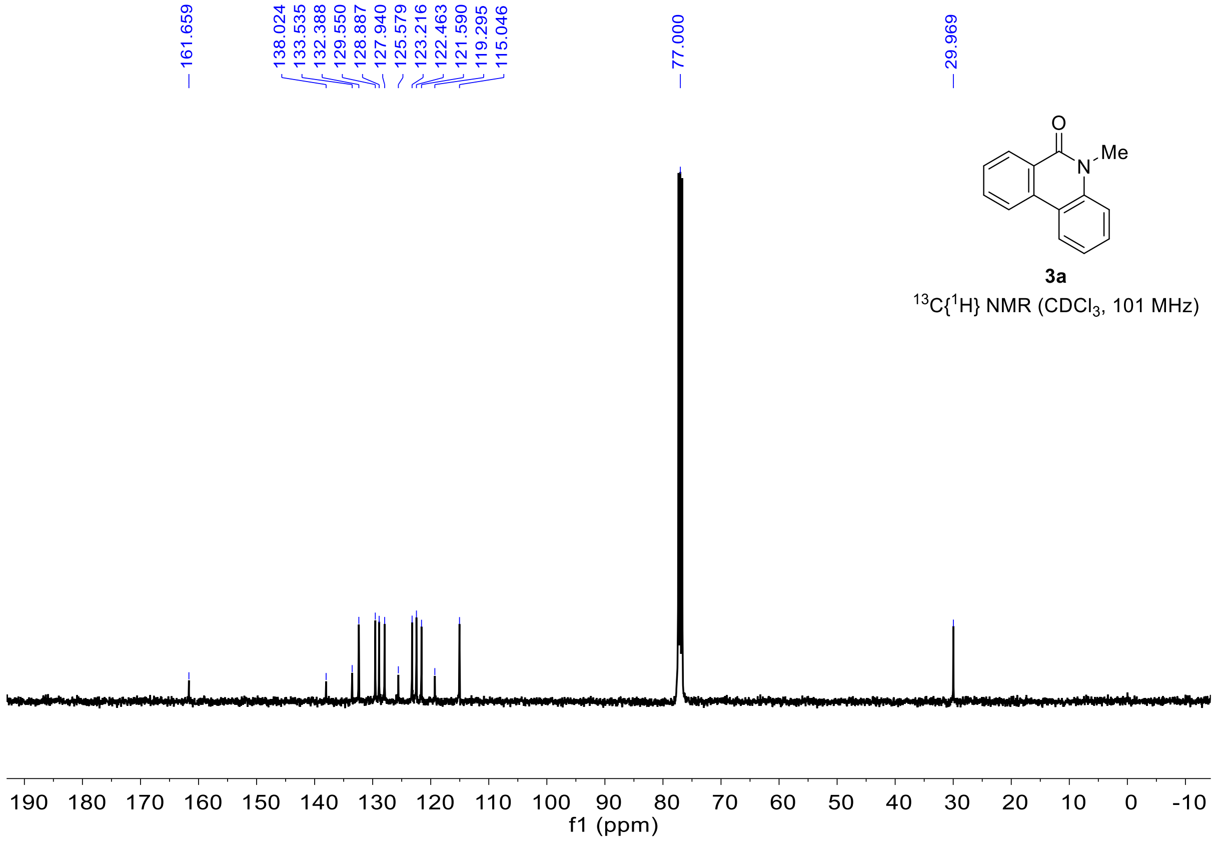

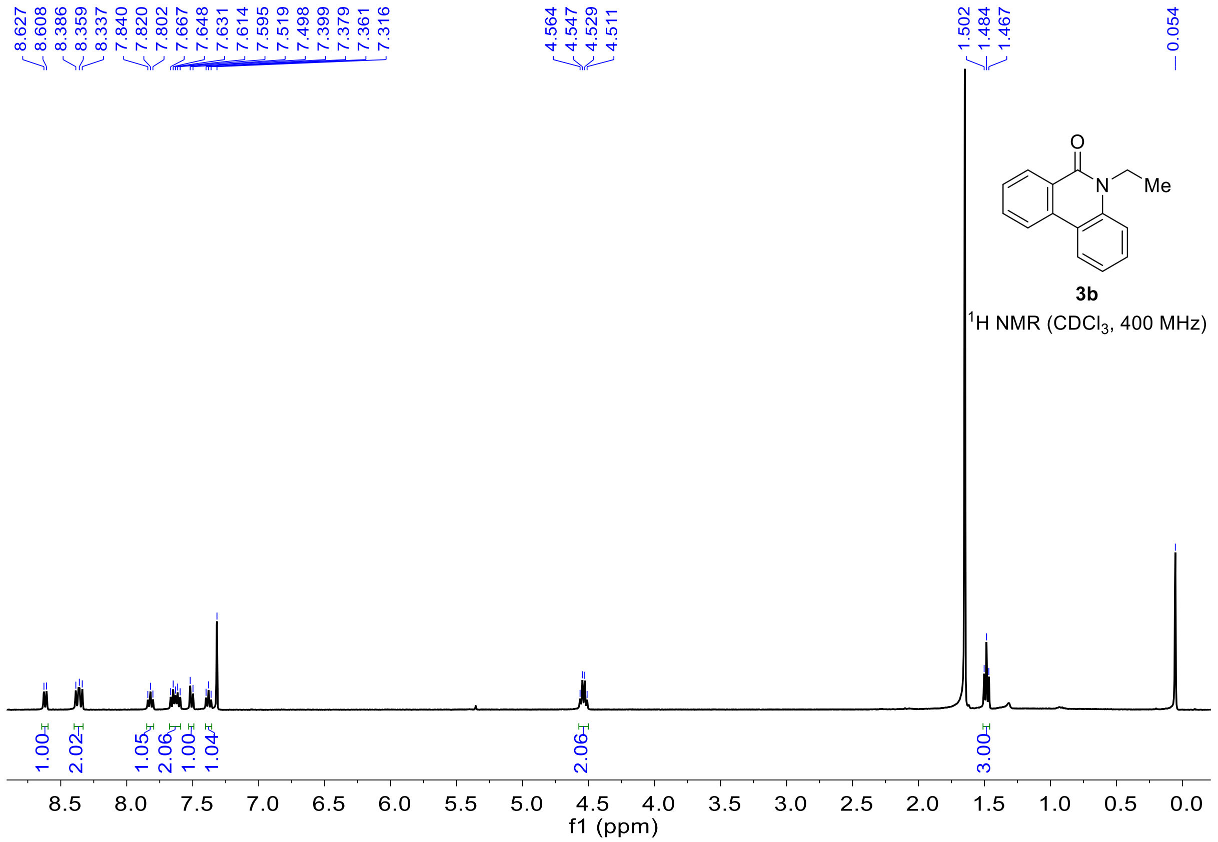
8.627
8.608
8.386
8.359
8.337
7.840
7.820
7.802
7.667
7.648
7.631
7.614
7.595
7.519
7.498
7.399
7.379
7.361
7.316
4.564
4.547
4.529
4.511
1.502
1.484
1.467
0.054
O
N
Me
3b
^{1}H NMR ($CDCl_3$, 400 MHz)
1.00
2.02
1.05
2.06
1.00
1.04
2.06
3.00
8.5
8.0
7.5
7.0
6.5
6.0
5.5
5.0
4.5
4.0
3.5
3.0
2.5
2.0
1.5
1.0
0.5
0.0
f1 (ppm)

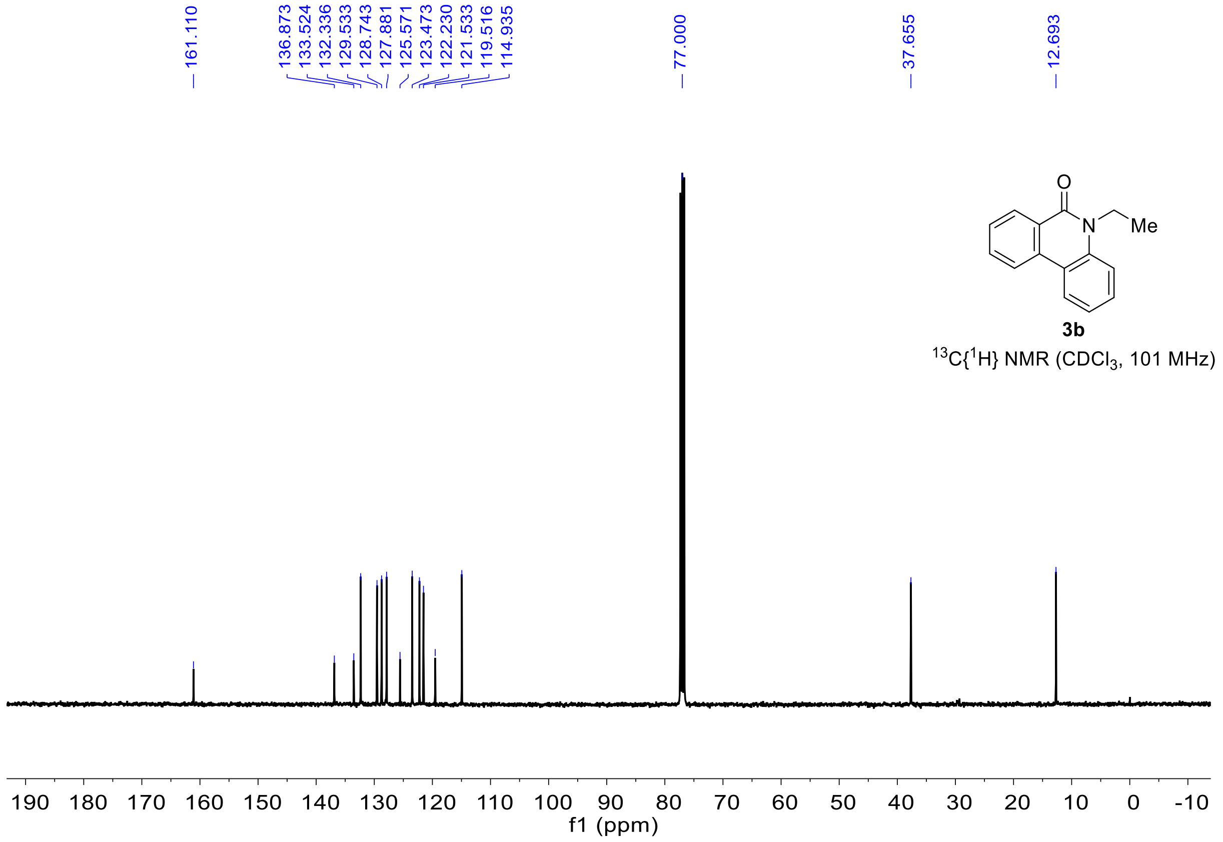
161.110
136.873
133.524
132.336
129.533
128.743
127.881
125.571
123.473
122.230
121.533
119.516
114.935
77.000
37.655
12.693
O
N
Me
3b
$^{13}C\{^{1}H\}$ NMR ($CDCl_3$, 101 MHz)
190
180
170
160
150
140
130
120
110
100
90
80
70
60
50
40
30
20
10
0
-10
f1 (ppm)

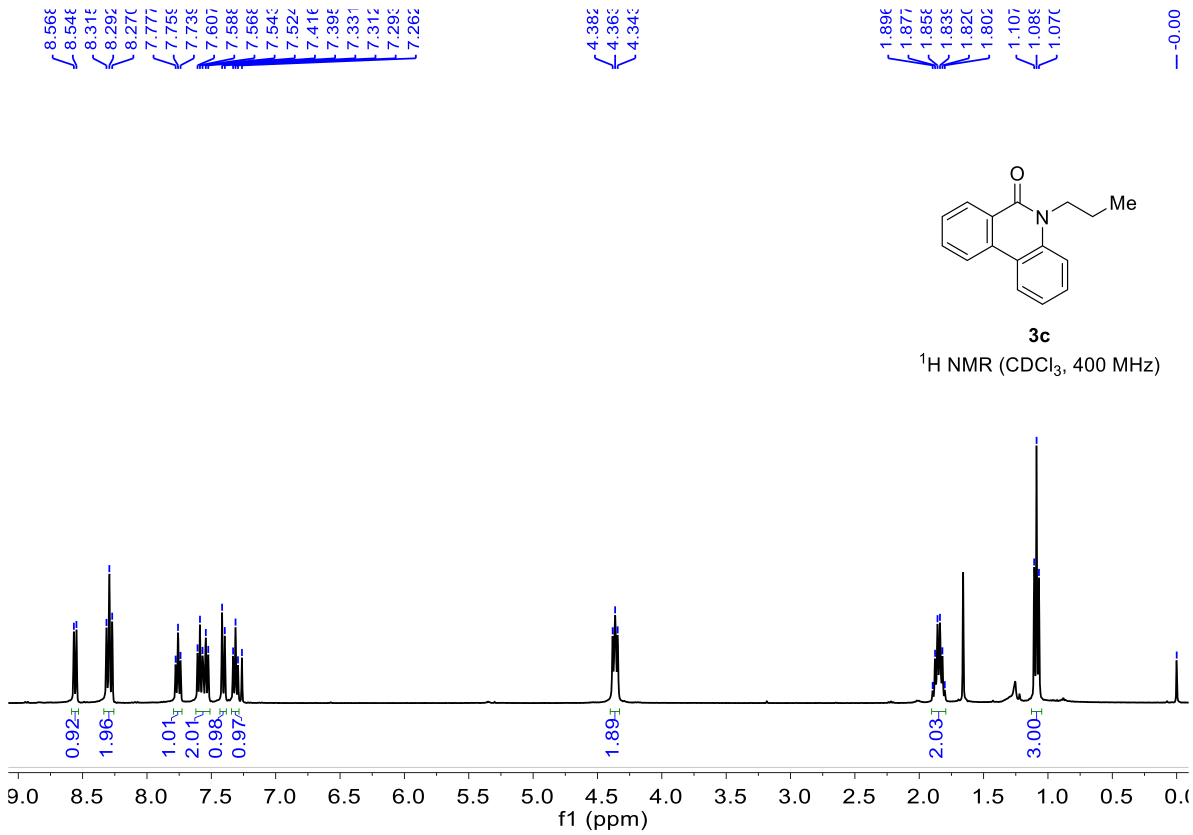

O
N
Me
3c
^{1}H NMR (CDCl$_3$, 400 MHz)
f1 (ppm)

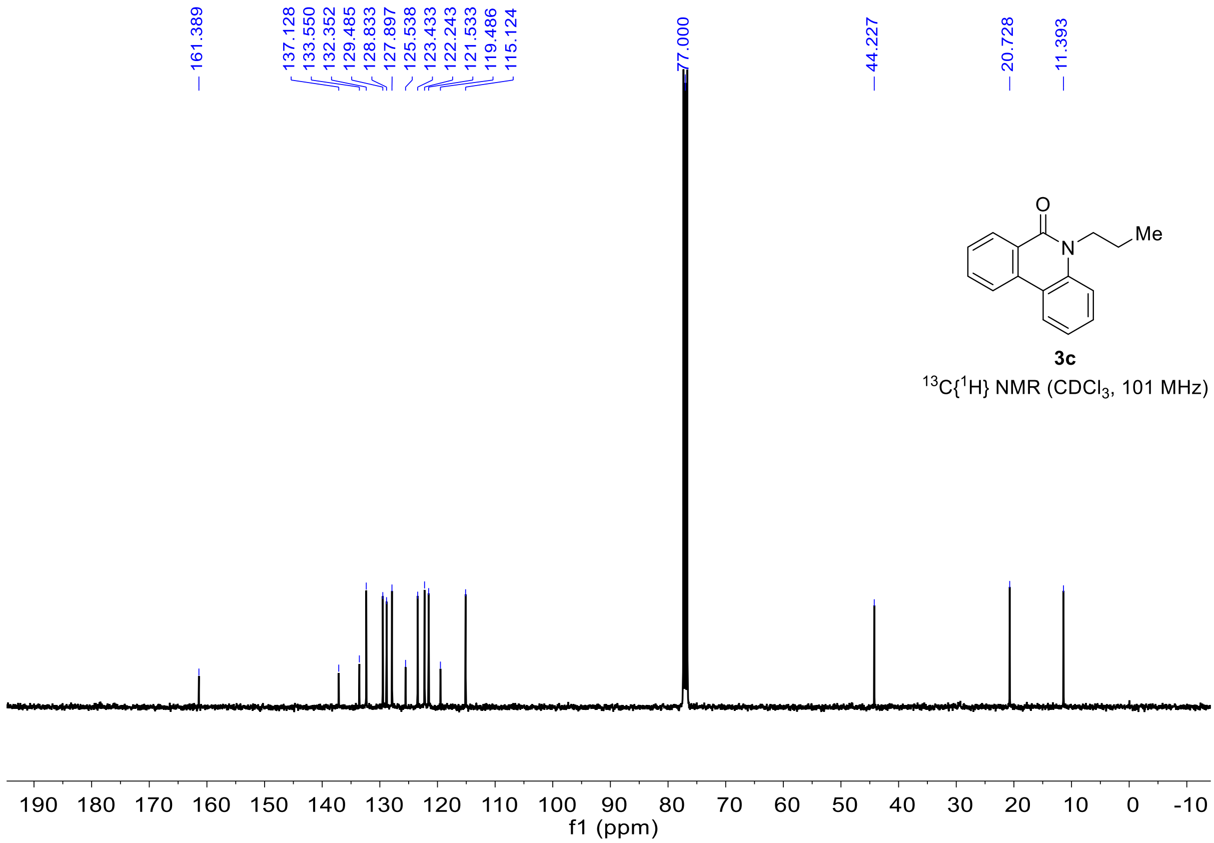

O
N
Me
3c
^{13}C{^{1}H} NMR (CDCl$_3$, 101 MHz)
f1 (ppm)

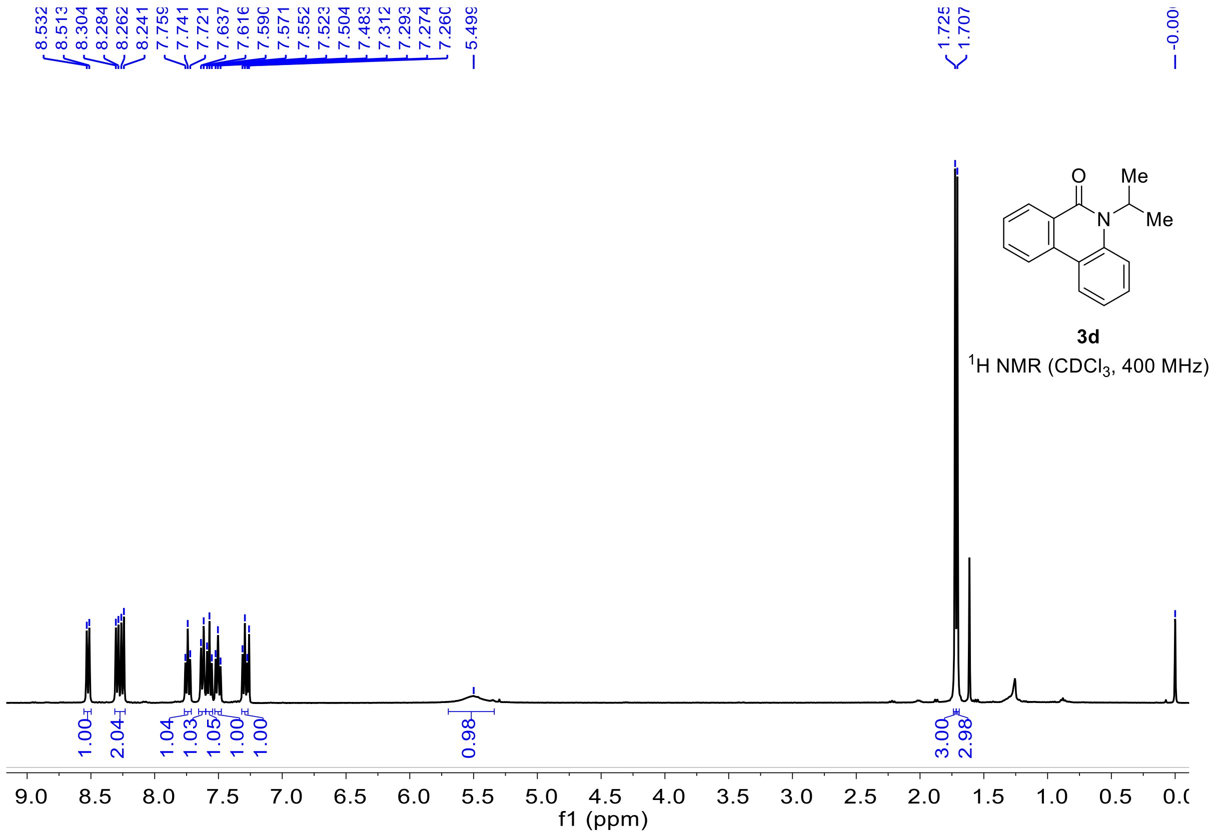
8.532
8.513
8.304
8.284
8.262
8.241
7.759
7.741
7.721
7.637
7.616
7.590
7.571
7.552
7.523
7.504
7.483
7.312
7.293
7.274
7.260
5.499
1.725
1.707
-0.000
1.00
2.04
1.04
1.03
1.05
1.00
1.00
0.98
3.00
2.98
O
Me
N
Me
3d
1H NMR (CDCl3, 400 MHz)
9.0 8.5 8.0 7.5 7.0 6.5 6.0 5.5 5.0 4.5 4.0 3.5 3.0 2.5 2.0 1.5 1.0 0.5 0.0
f1 (ppm)

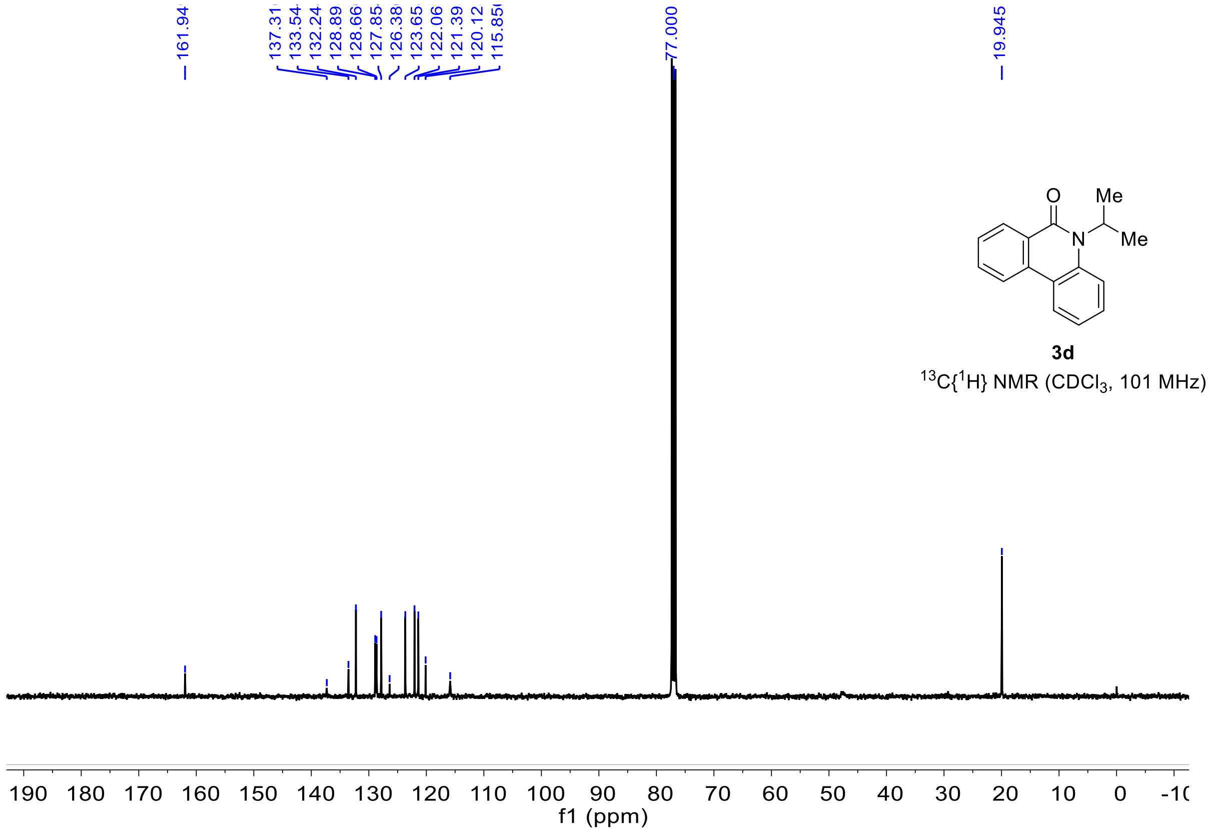
161.94
137.31
133.54
132.24
128.89
128.66
127.85
126.38
123.65
122.06
121.39
120.12
115.85
77.000
19.945
O
Me
N
Me
3d
13C{1H} NMR (CDCl3, 101 MHz)
190 180 170 160 150 140 130 120 110 100 90 80 70 60 50 40 30 20 10 0 -10
f1 (ppm)

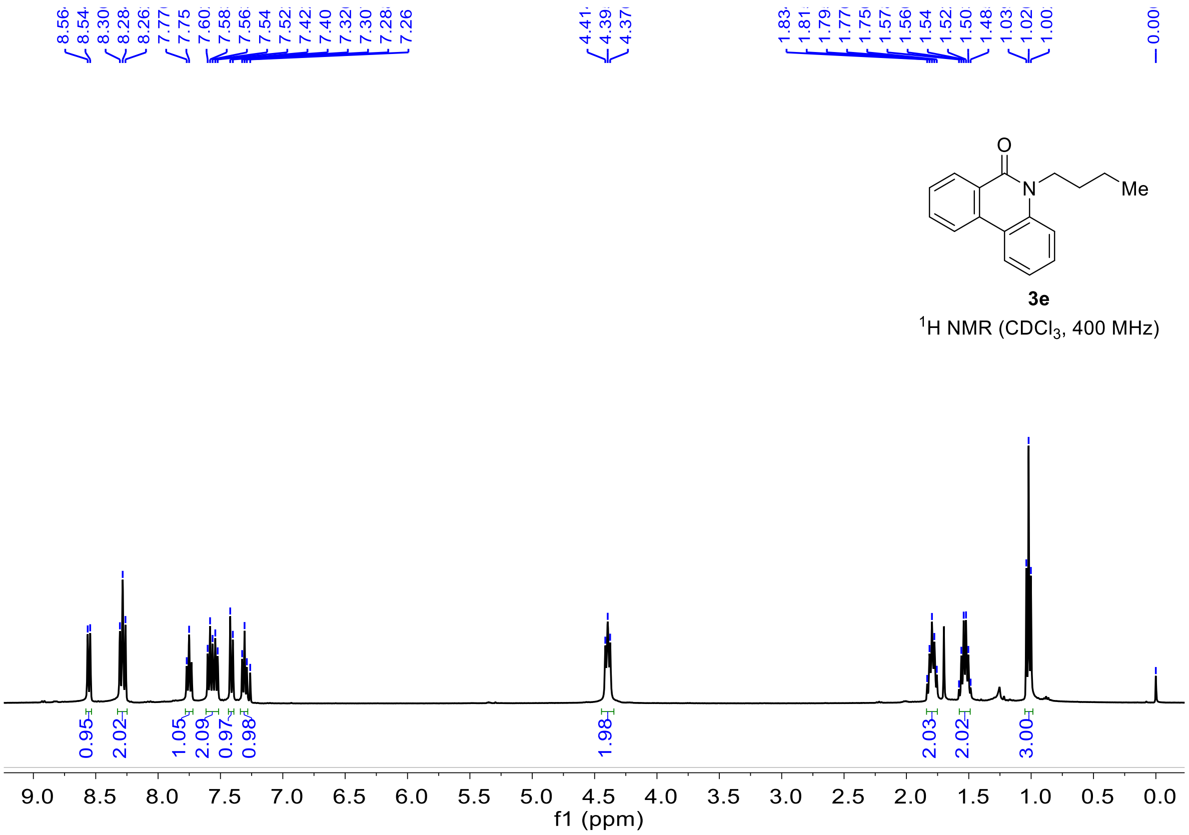

3e

^{1}H NMR ($CDCl_3$, 400 MHz)

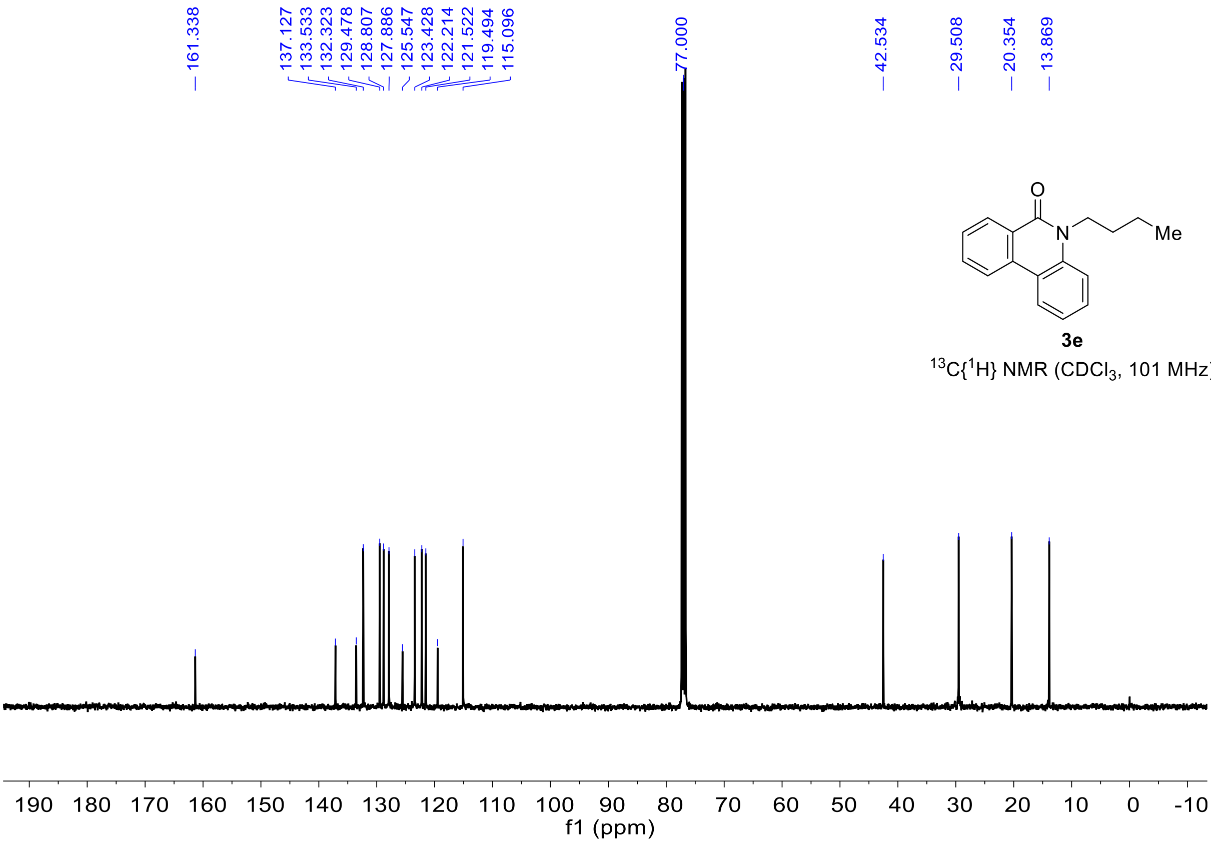

3e

$^{13}C\{^1H\}$ NMR ($CDCl_3$, 101 MHz)

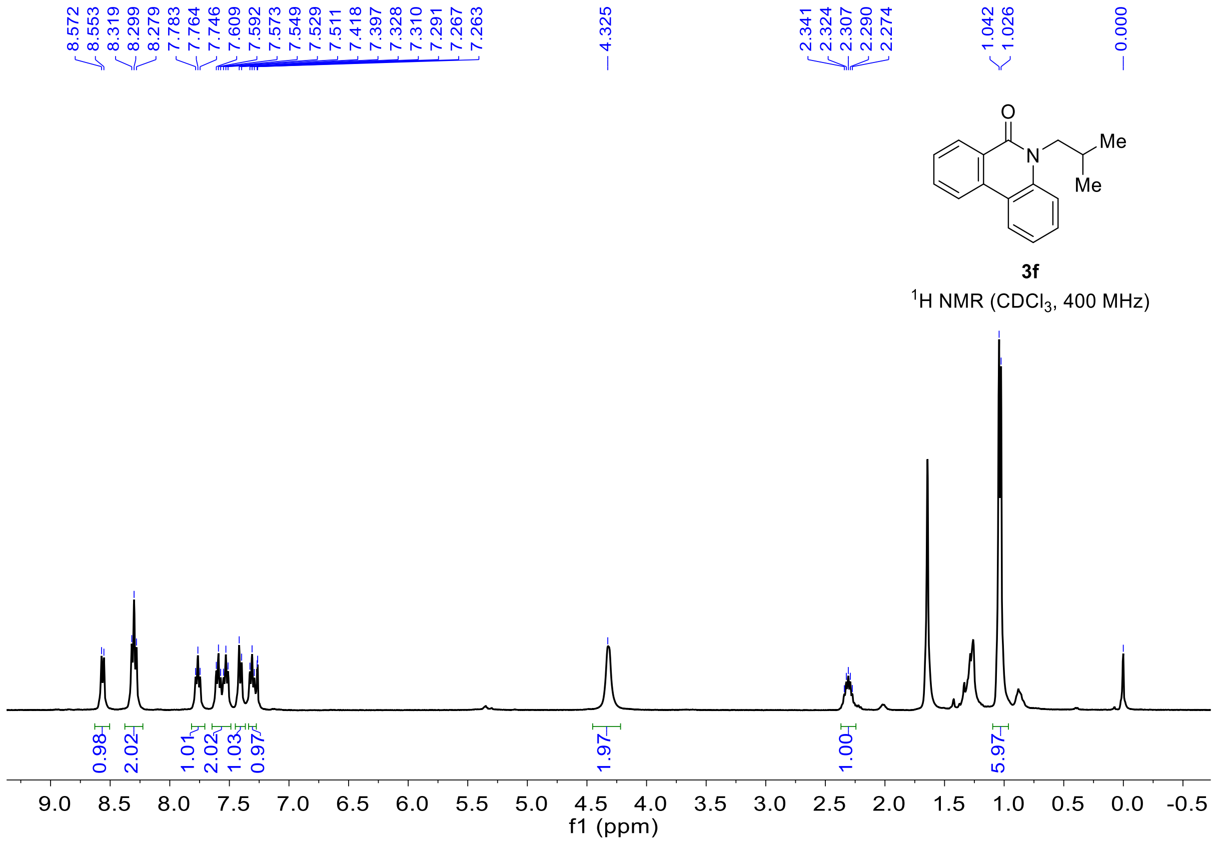
8.572
8.553
8.319
8.299
8.279
7.783
7.764
7.746
7.609
7.592
7.573
7.549
7.529
7.511
7.418
7.397
7.328
7.310
7.291
7.267
7.263
4.325
2.341
2.324
2.307
2.290
2.274
1.042
1.026
0.000
O
N
Me
Me
3f
1H NMR (CDCl3, 400 MHz)
0.98
2.02
1.01
2.02
1.03
0.97
1.97
1.00
5.97
9.0 8.5 8.0 7.5 7.0 6.5 6.0 5.5 5.0 4.5 4.0 3.5 3.0 2.5 2.0 1.5 1.0 0.5 0.0 -0.5
f1 (ppm)

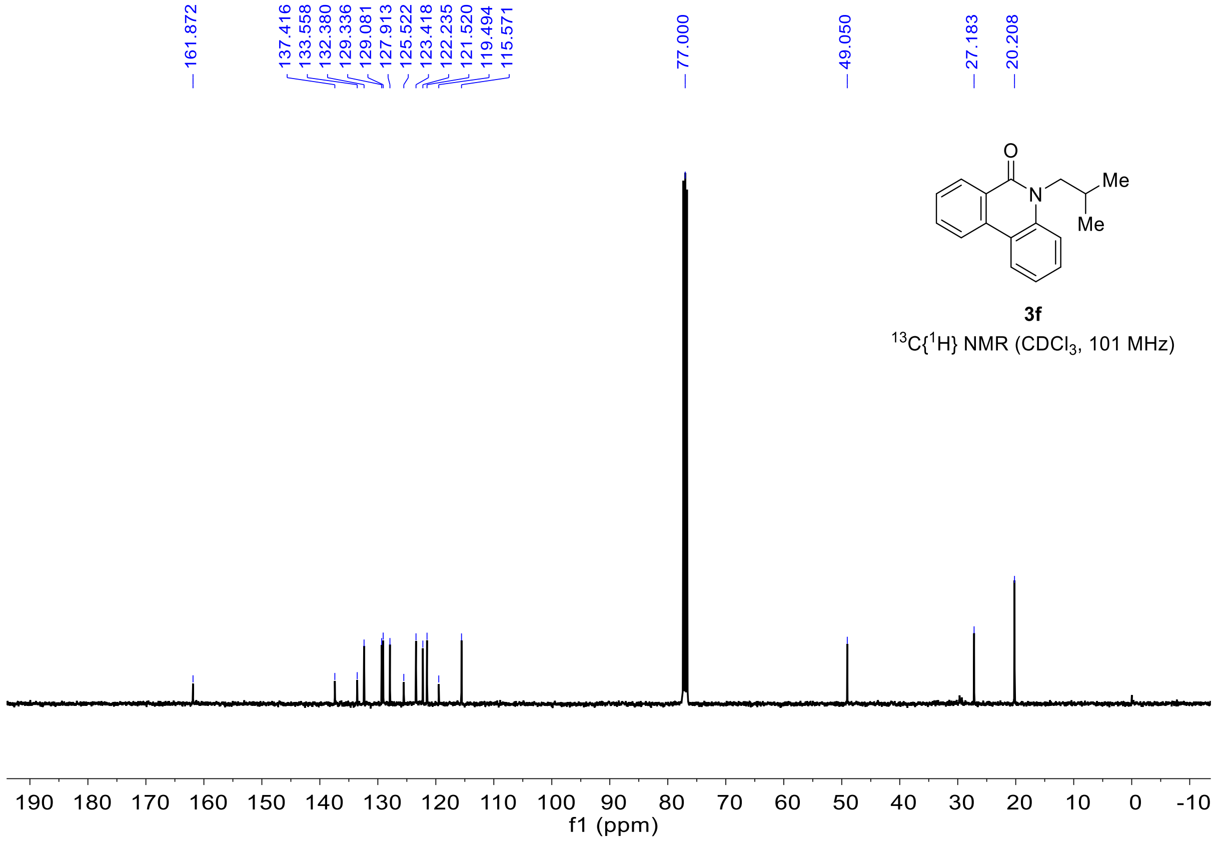
161.872
137.416
133.558
132.380
129.336
129.081
127.913
125.522
123.418
122.235
121.520
119.494
115.571
77.000
49.050
27.183
20.208
O
N
Me
Me
3f
13C{1H} NMR (CDCl3, 101 MHz)
190 180 170 160 150 140 130 120 110 100 90 80 70 60 50 40 30 20 10 0 -10
f1 (ppm)

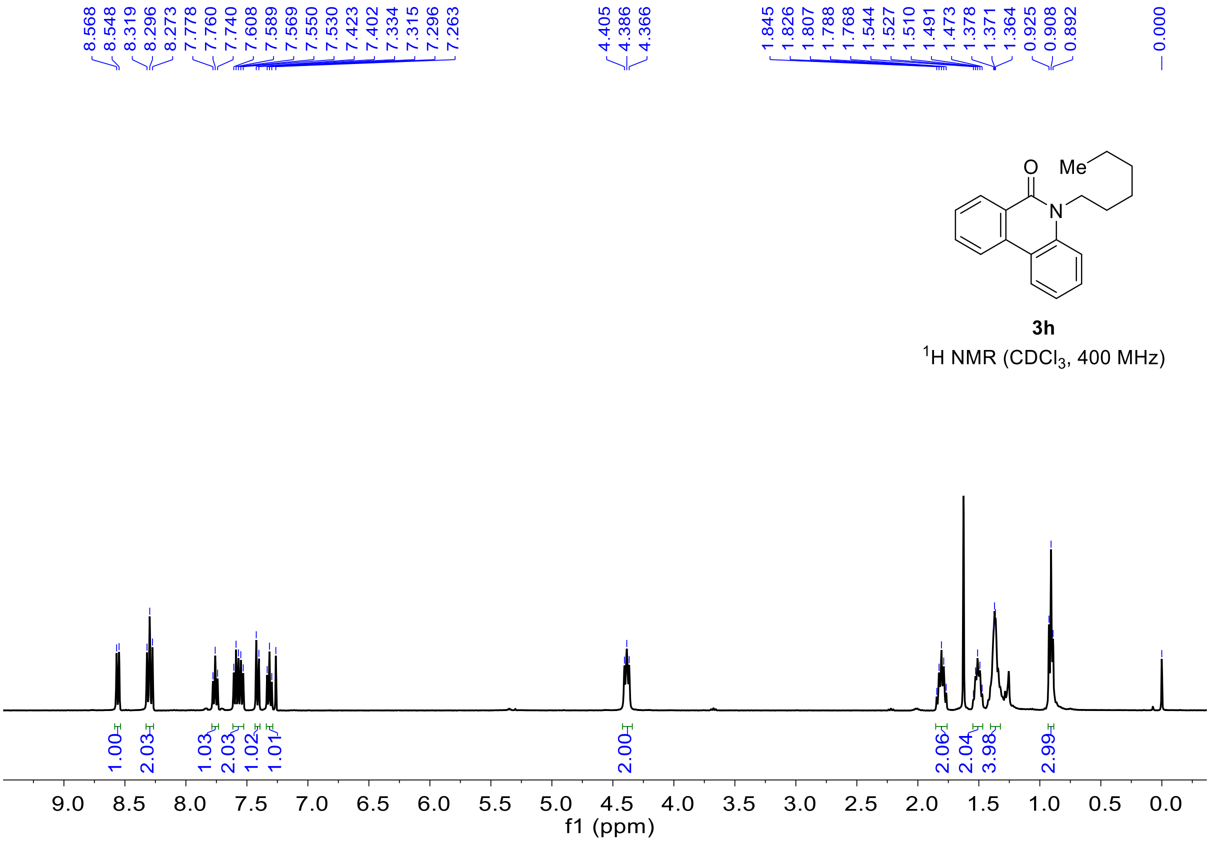
8.568 8.548 8.319 8.296 8.273 7.778 7.760 7.740 7.608 7.589 7.569 7.550 7.530 7.423 7.402 7.334 7.315 7.296 7.263
4.405 4.386 4.366
1.845 1.826 1.807 1.788 1.768 1.544 1.527 1.510 1.491 1.473 1.378 1.371 1.364 0.925 0.908 0.892
0.000
O
Me
N
3h
^{1}H NMR (CDCl$_3$, 400 MHz)
1.00 2.03 1.03 2.03 1.02 1.01 2.00 2.06 2.04 3.98 2.99
9.0 8.5 8.0 7.5 7.0 6.5 6.0 5.5 5.0 4.5 4.0 3.5 3.0 2.5 2.0 1.5 1.0 0.5 0.0
f1 (ppm)

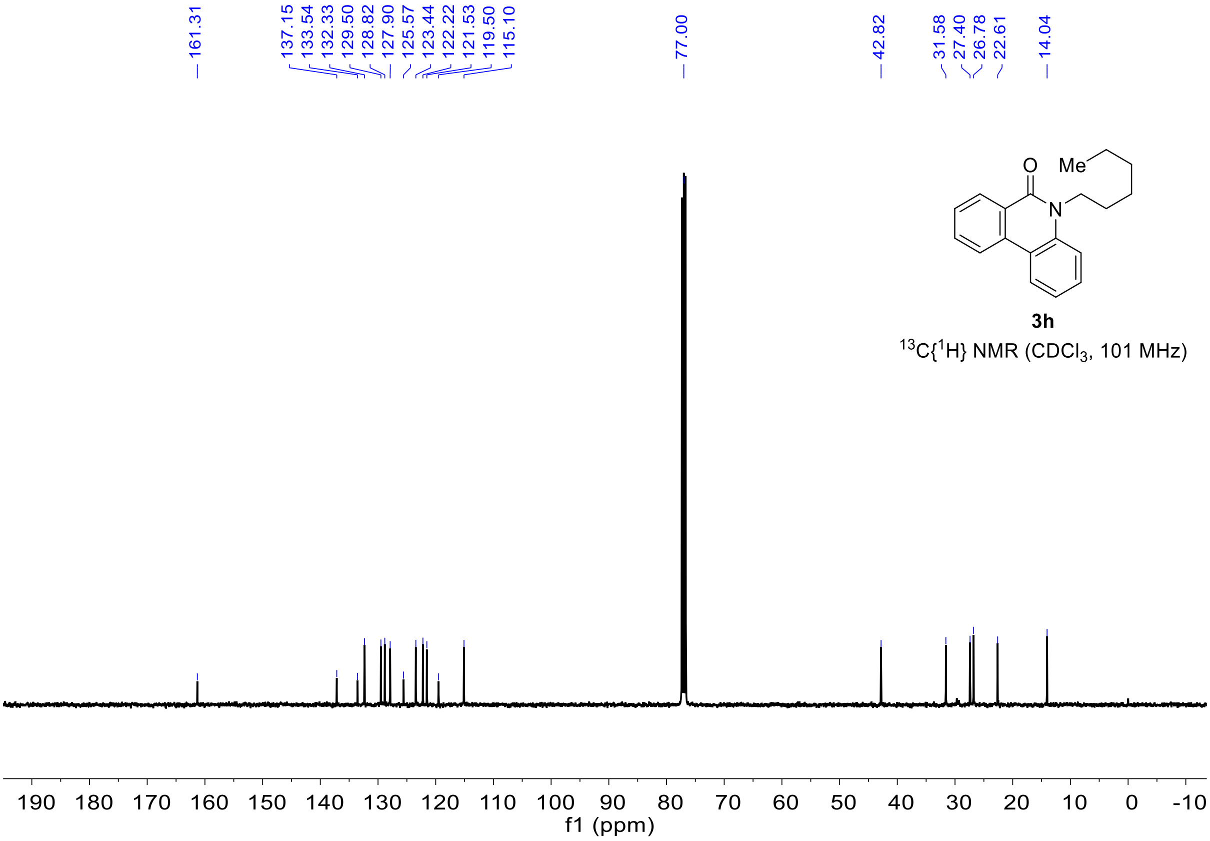
161.31
137.15 133.54 132.33 129.50 128.82 127.90 125.57 123.44 122.22 121.53 119.50 115.10
77.00
42.82
31.58 27.40 26.78 22.61
14.04
O
Me
N
3h
^{13}C{^{1}H} NMR (CDCl$_3$, 101 MHz)
190 180 170 160 150 140 130 120 110 100 90 80 70 60 50 40 30 20 10 0 -10
f1 (ppm)

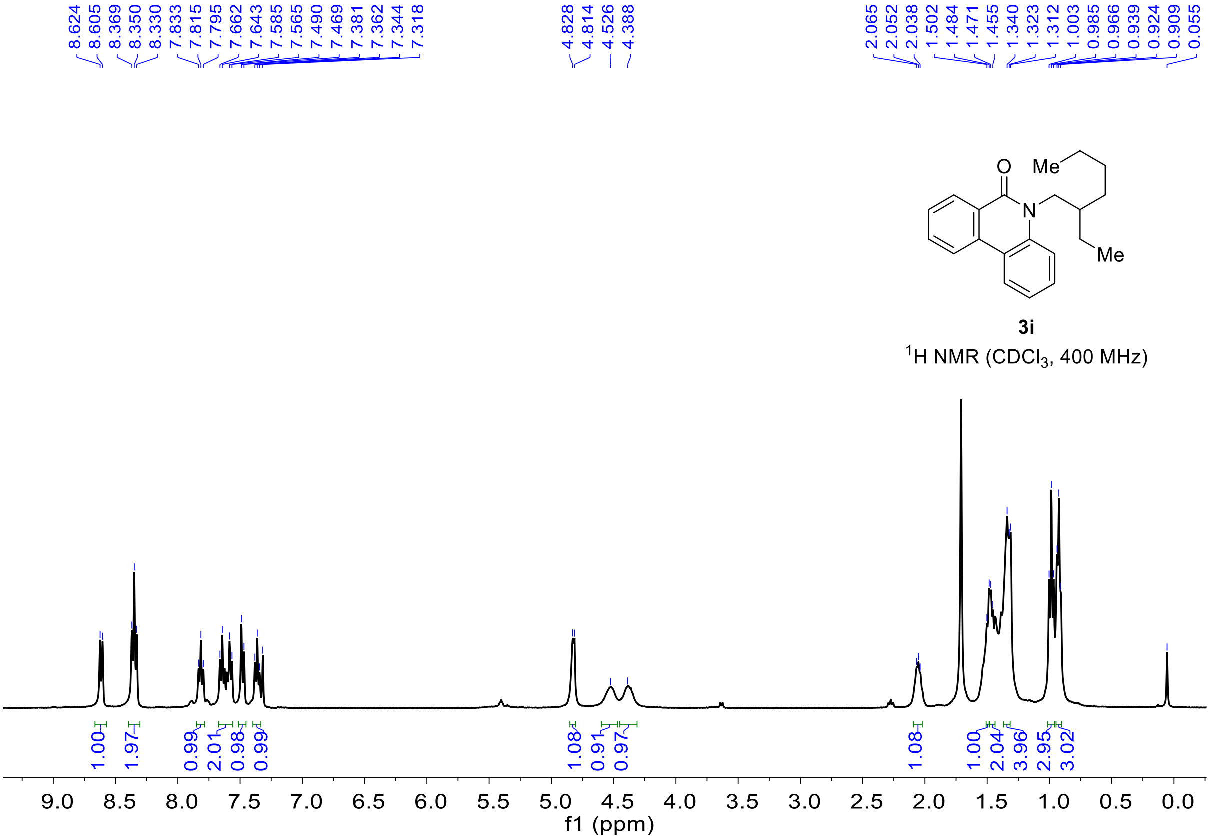
8.624
8.605
8.369
8.350
8.330
7.833
7.815
7.795
7.662
7.643
7.585
7.565
7.490
7.469
7.381
7.362
7.344
7.318
4.828
4.814
4.526
4.388
2.065
2.052
2.038
1.502
1.484
1.471
1.455
1.340
1.323
1.312
1.003
0.985
0.966
0.939
0.924
0.909
0.055
O
N
Me
Me
3i
1H NMR (CDCl3, 400 MHz)
1.00
1.97
0.99
2.01
0.98
0.99
1.08
0.91
0.97
1.08
1.00
2.04
3.96
2.95
3.02
f1 (ppm)

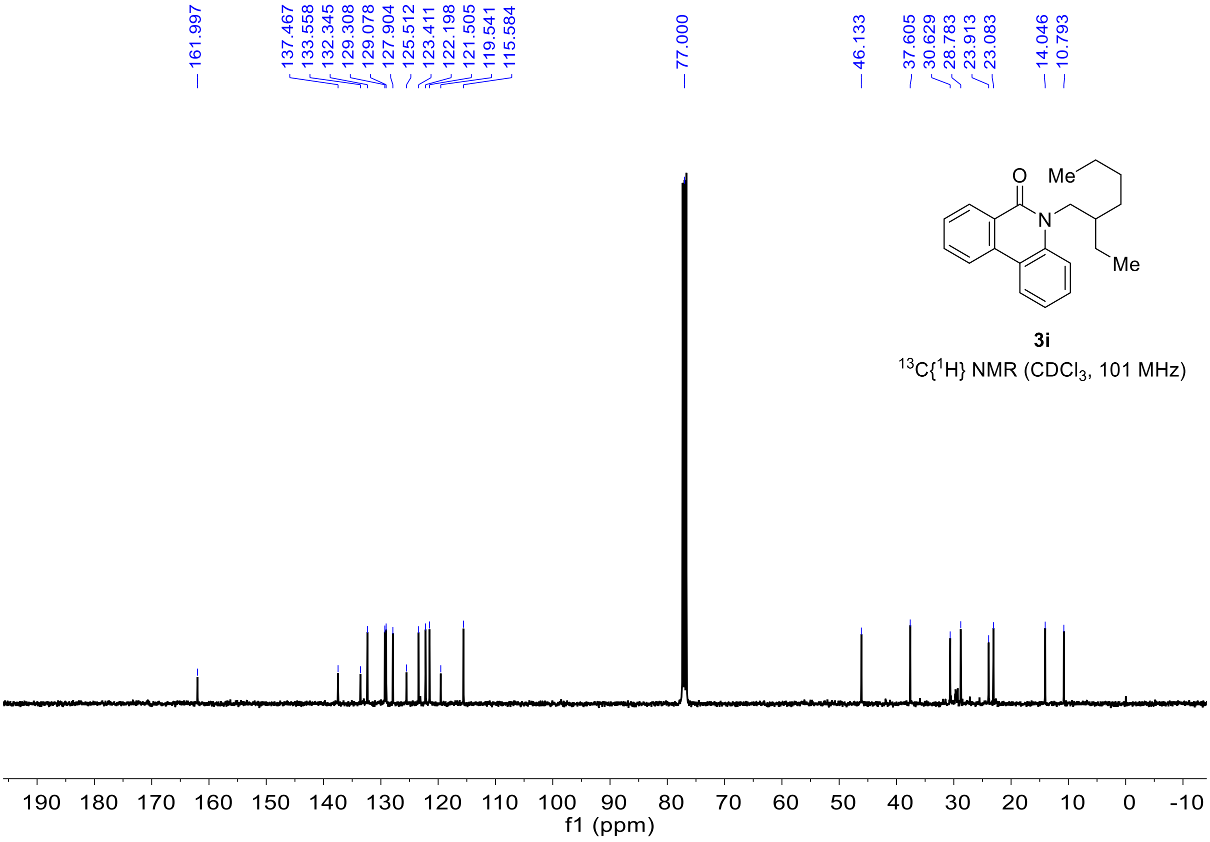
161.997
137.467
133.558
132.345
129.308
129.078
127.904
125.512
123.411
122.198
121.505
119.541
115.584
77.000
46.133
37.605
30.629
28.783
23.913
23.083
14.046
10.793
O
N
Me
Me
3i
13C{1H} NMR (CDCl3, 101 MHz)
f1 (ppm)

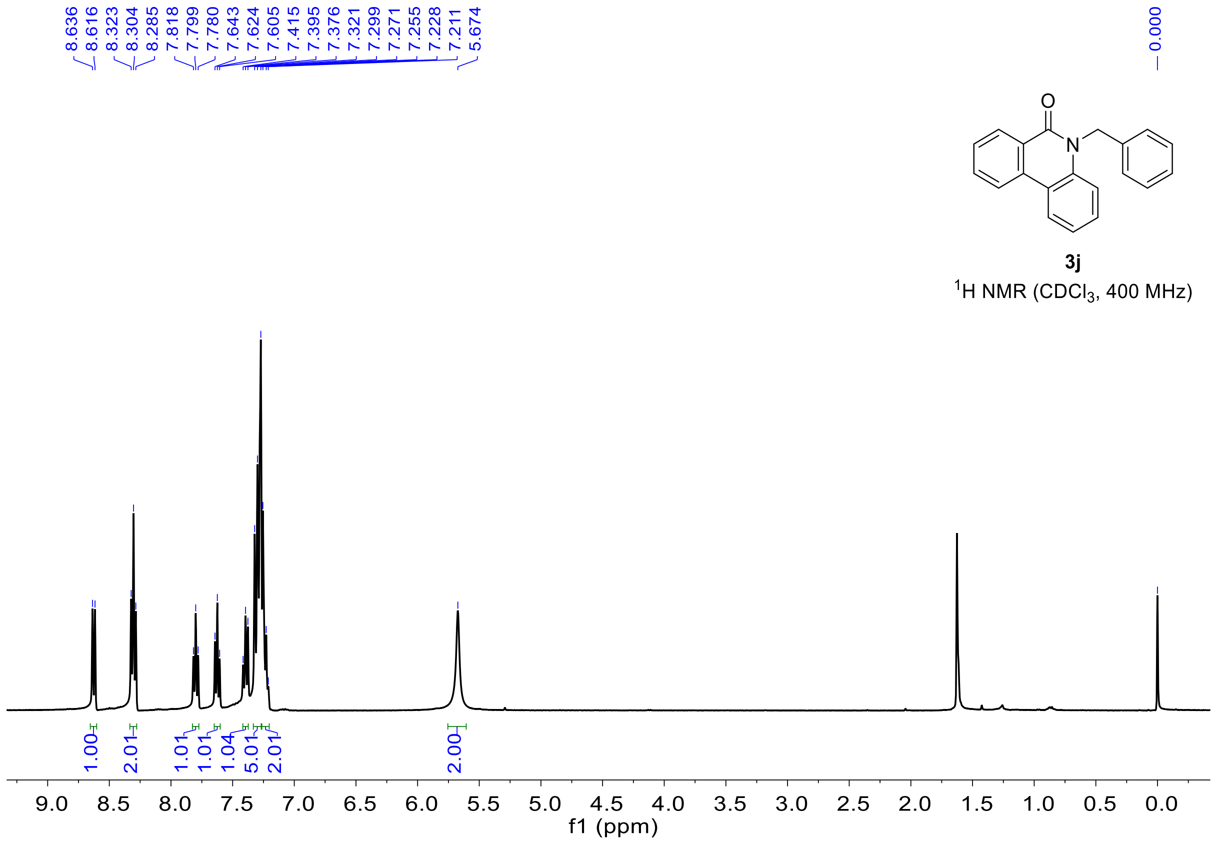
8.636
8.616
8.323
8.304
8.285
7.818
7.799
7.780
7.643
7.624
7.605
7.415
7.395
7.376
7.321
7.299
7.271
7.255
7.228
7.211
5.674
0.000
O
N
3j
^{1}H NMR (CDCl$_3$, 400 MHz)
1.00
2.01
1.01
1.01
1.04
5.01
2.01
2.00
9.0 8.5 8.0 7.5 7.0 6.5 6.0 5.5 5.0 4.5 4.0 3.5 3.0 2.5 2.0 1.5 1.0 0.5 0.0
f1 (ppm)

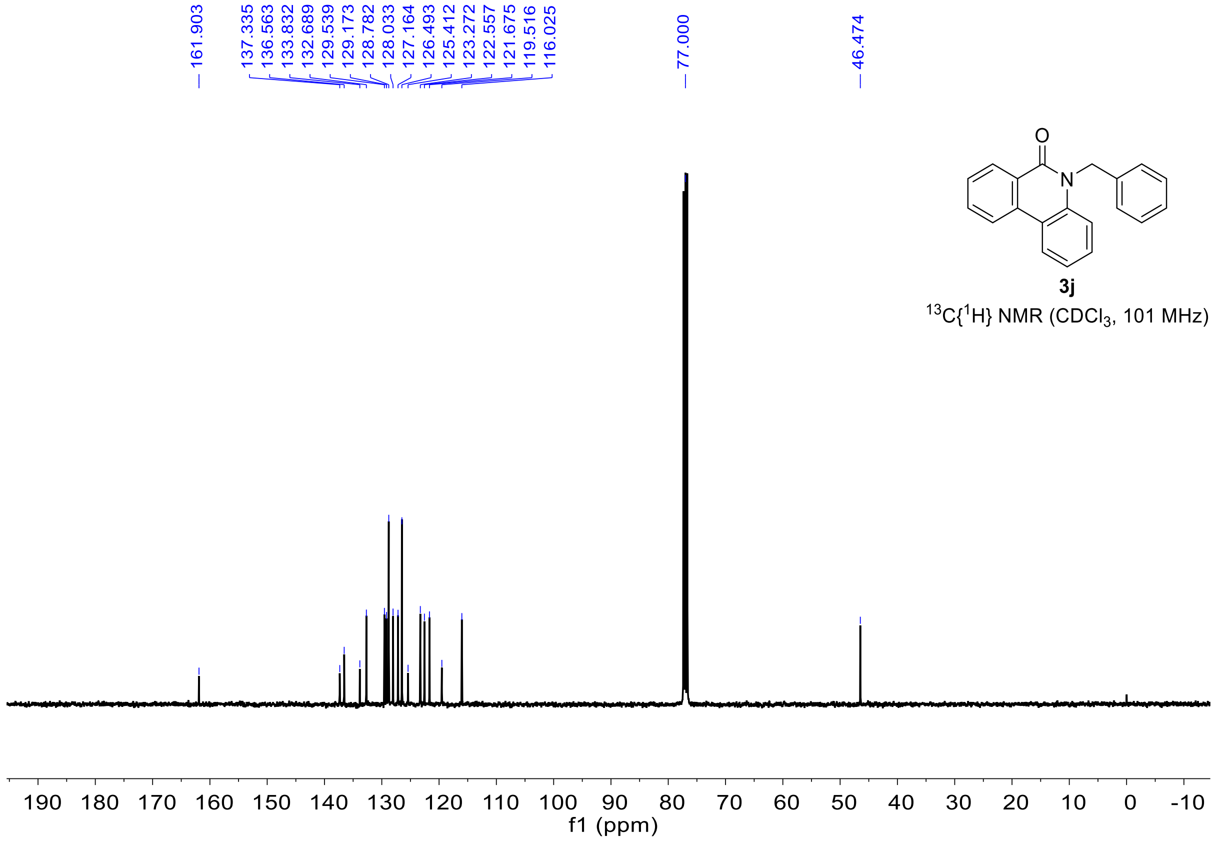
161.903
137.335
136.563
133.832
132.689
129.539
129.173
128.782
128.033
127.164
126.493
125.412
123.272
122.557
121.675
119.516
116.025
77.000
46.474
O
N
3j
^{13}C{^{1}H} NMR (CDCl$_3$, 101 MHz)
190 180 170 160 150 140 130 120 110 100 90 80 70 60 50 40 30 20 10 0 -10
f1 (ppm)

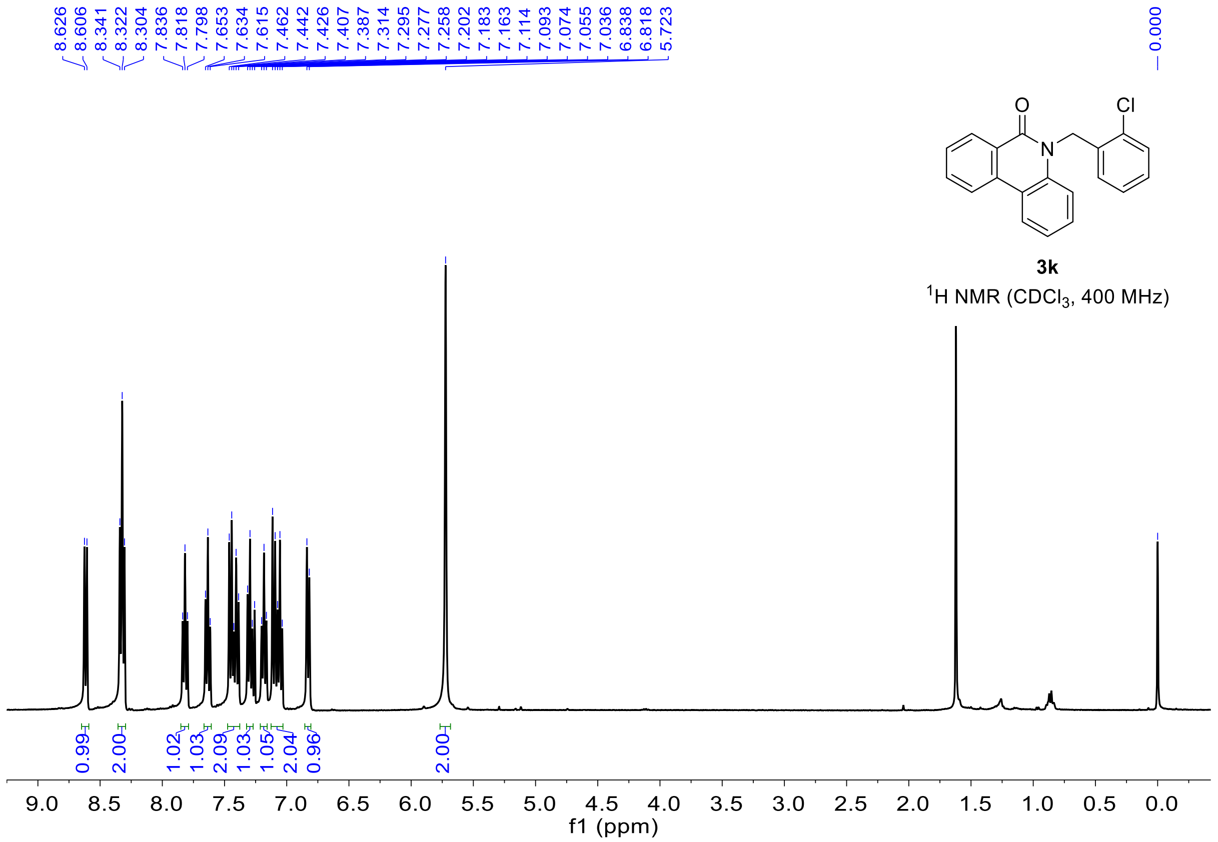

8.626
8.606
8.341
8.322
8.304
7.836
7.818
7.798
7.653
7.634
7.615
7.462
7.442
7.426
7.407
7.387
7.314
7.295
7.277
7.258
7.202
7.183
7.163
7.114
7.093
7.074
7.055
7.036
6.838
6.818
5.723
0.000
O
Cl
N
3k
1H NMR (CDCl3, 400 MHz)
0.99
2.00
1.02
1.03
2.09
1.03
1.05
2.04
0.96
2.00
9.0 8.5 8.0 7.5 7.0 6.5 6.0 5.5 5.0 4.5 4.0 3.5 3.0 2.5 2.0 1.5 1.0 0.5 0.0
f1 (ppm)

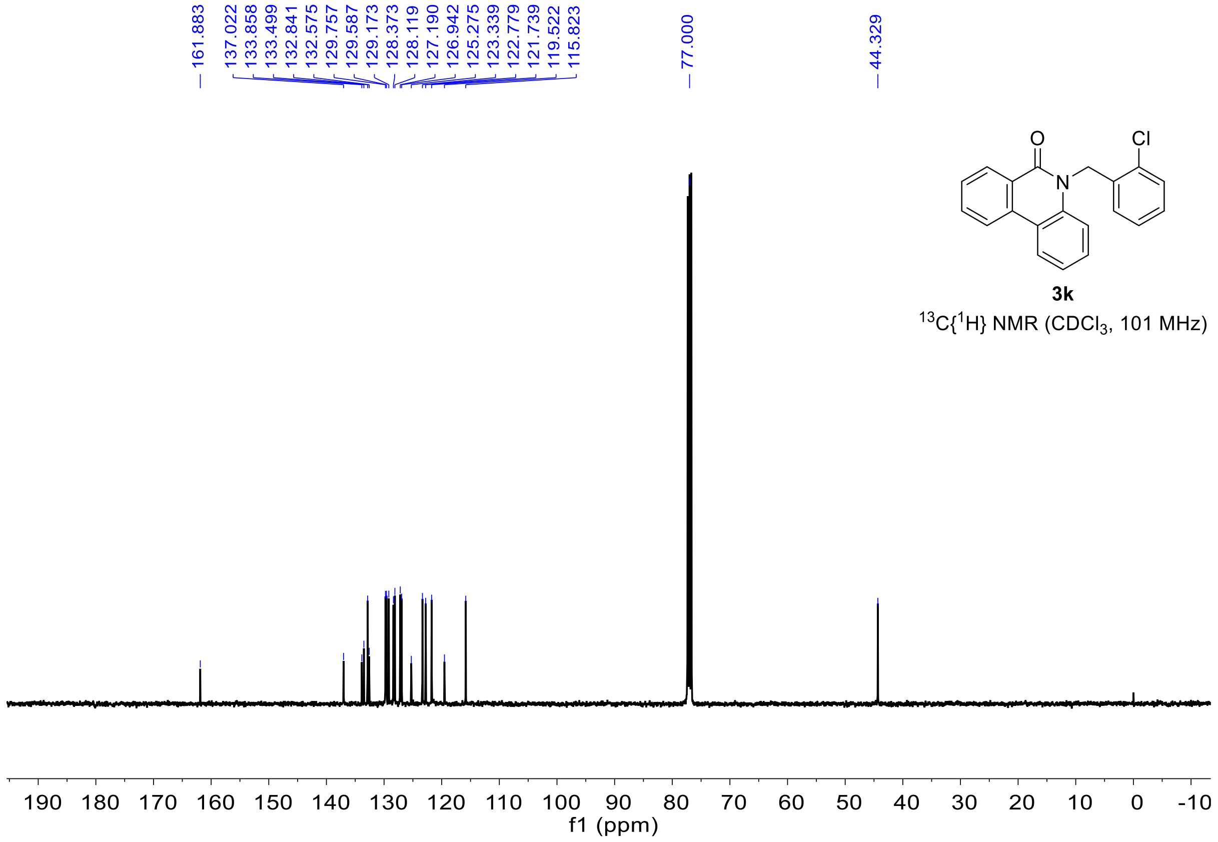

161.883
137.022
133.858
133.499
132.841
132.575
129.757
129.587
129.173
128.373
128.119
127.190
126.942
125.275
123.339
122.779
121.739
119.522
115.823
77.000
44.329
O
Cl
N
3k
13C{1H} NMR (CDCl3, 101 MHz)
190 180 170 160 150 140 130 120 110 100 90 80 70 60 50 40 30 20 10 0 -10
f1 (ppm)

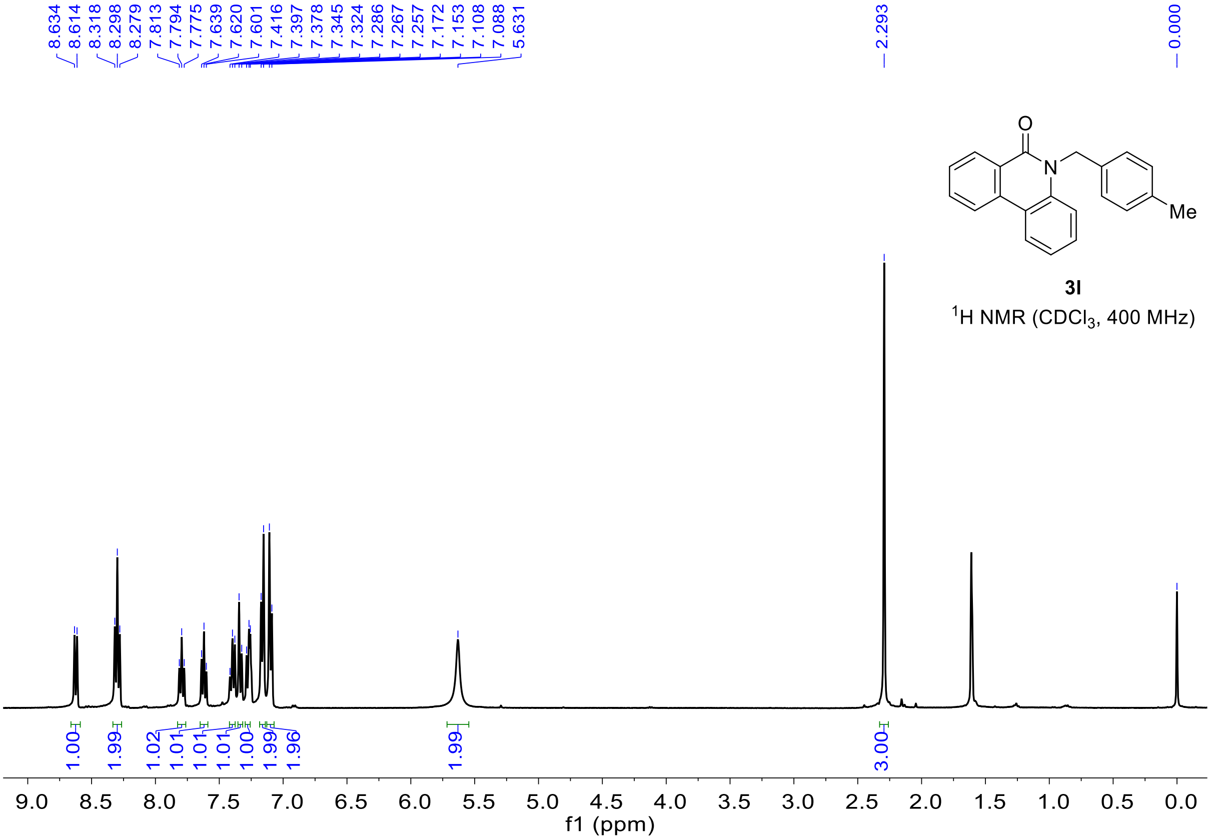
8.634
8.614
8.318
8.298
8.279
7.813
7.794
7.775
7.639
7.620
7.601
7.416
7.397
7.378
7.345
7.324
7.286
7.267
7.257
7.172
7.153
7.108
7.088
5.631
2.293
0.000
O
N
Me
3l
^{1}H NMR ($CDCl_3$, 400 MHz)
1.00
1.99
1.02
1.01
1.01
1.01
1.00
1.99
1.96
1.99
3.00
9.0 8.5 8.0 7.5 7.0 6.5 6.0 5.5 5.0 4.5 4.0 3.5 3.0 2.5 2.0 1.5 1.0 0.5 0.0
f1 (ppm)

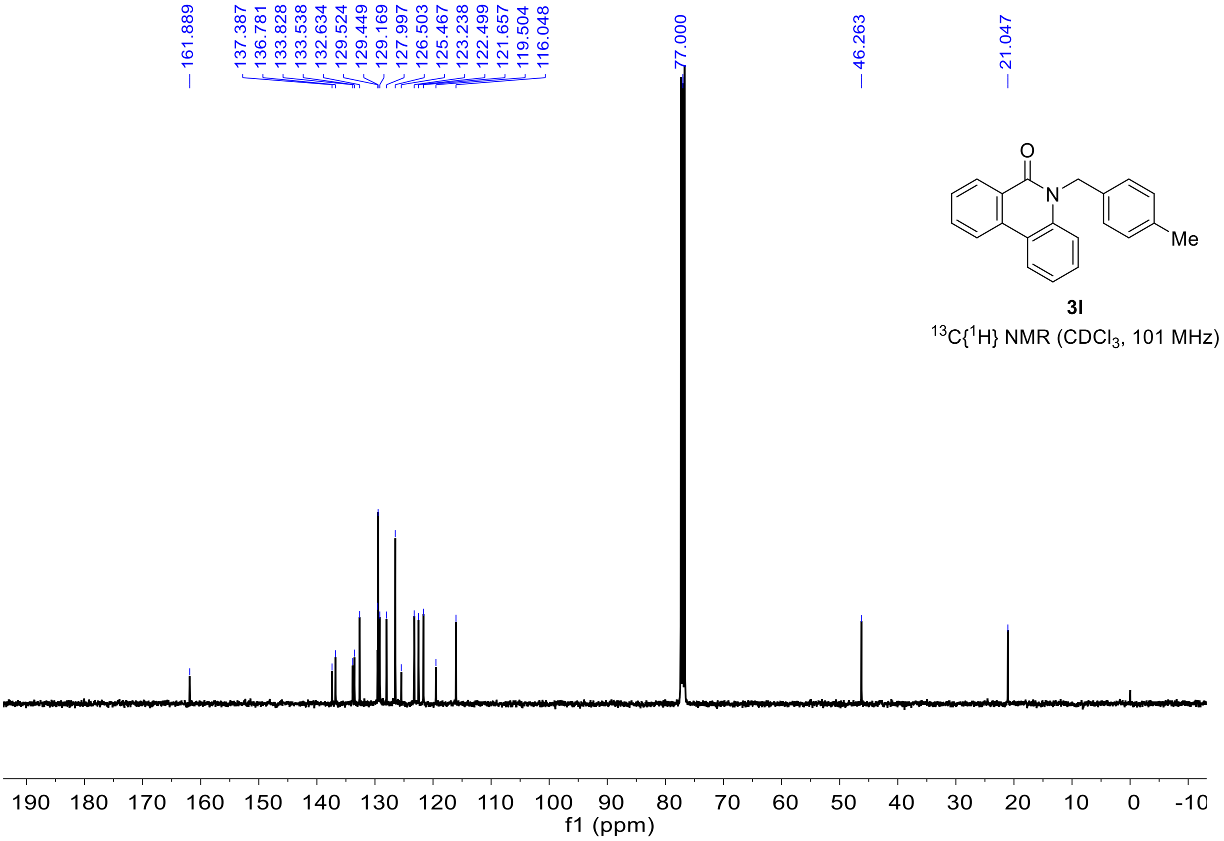
161.889
137.387
136.781
133.828
133.538
132.634
129.524
129.449
129.169
127.997
126.503
125.467
123.238
122.499
121.657
119.504
116.048
77.000
46.263
21.047
O
N
Me
3l
^{13}C{^{1}H} NMR ($CDCl_3$, 101 MHz)
190 180 170 160 150 140 130 120 110 100 90 80 70 60 50 40 30 20 10 0 -10
f1 (ppm)

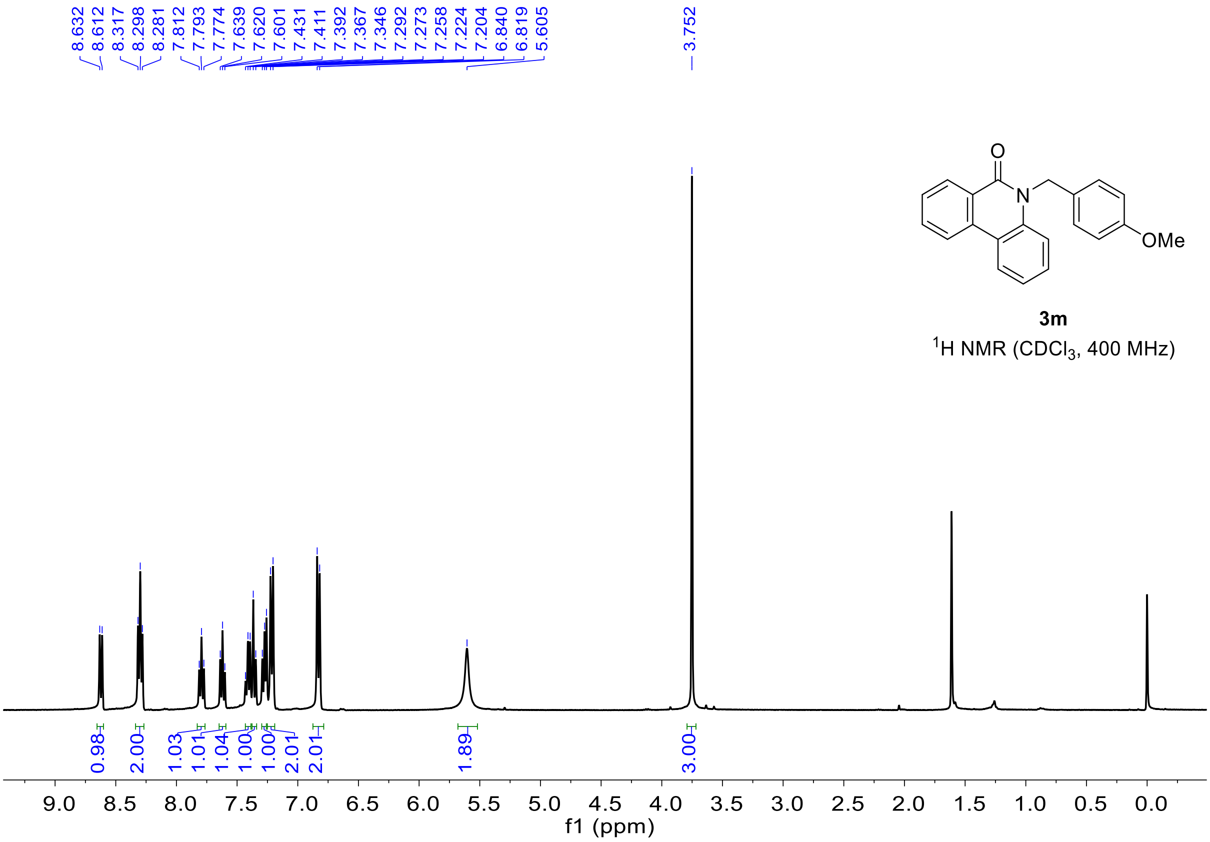

3m

^{1}H NMR ($CDCl_3$, 400 MHz)

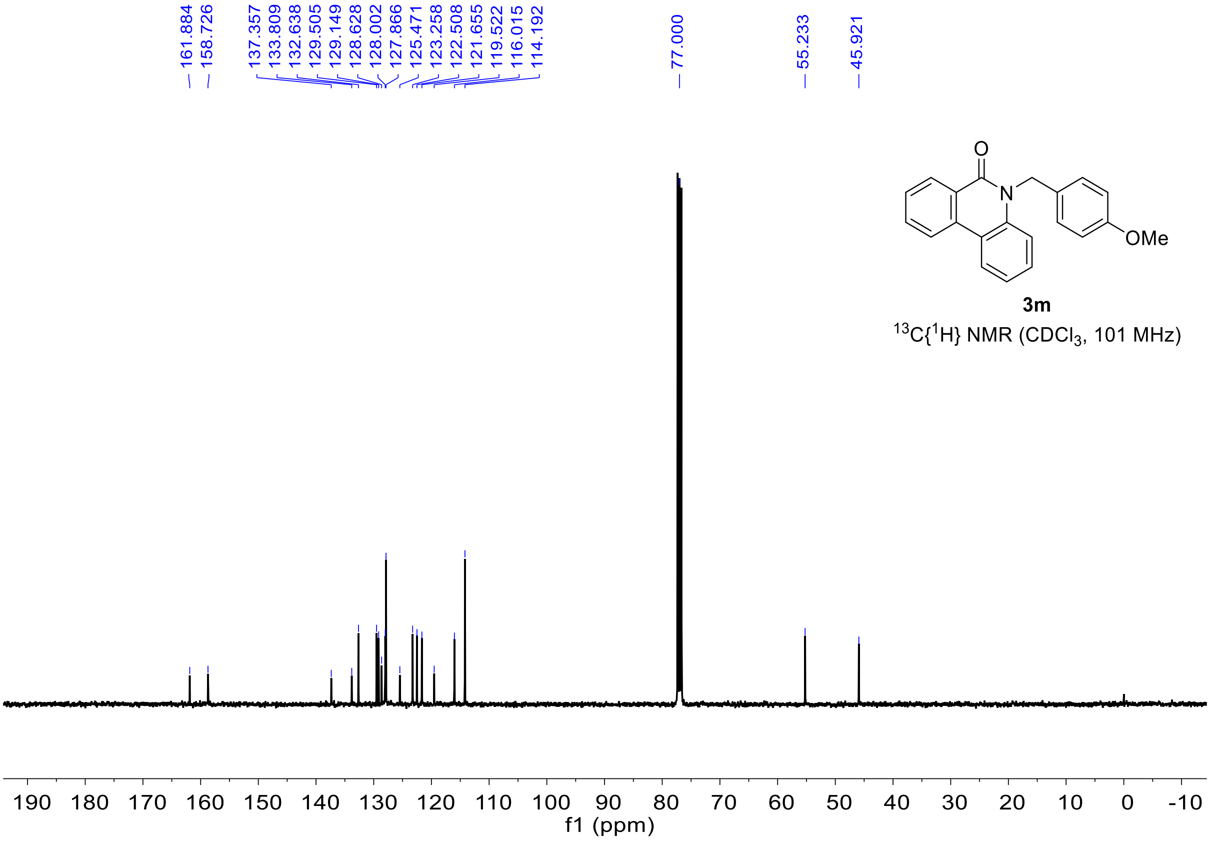

3m

$^{13}C\{^{1}H\}$ NMR ($CDCl_3$, 101 MHz)

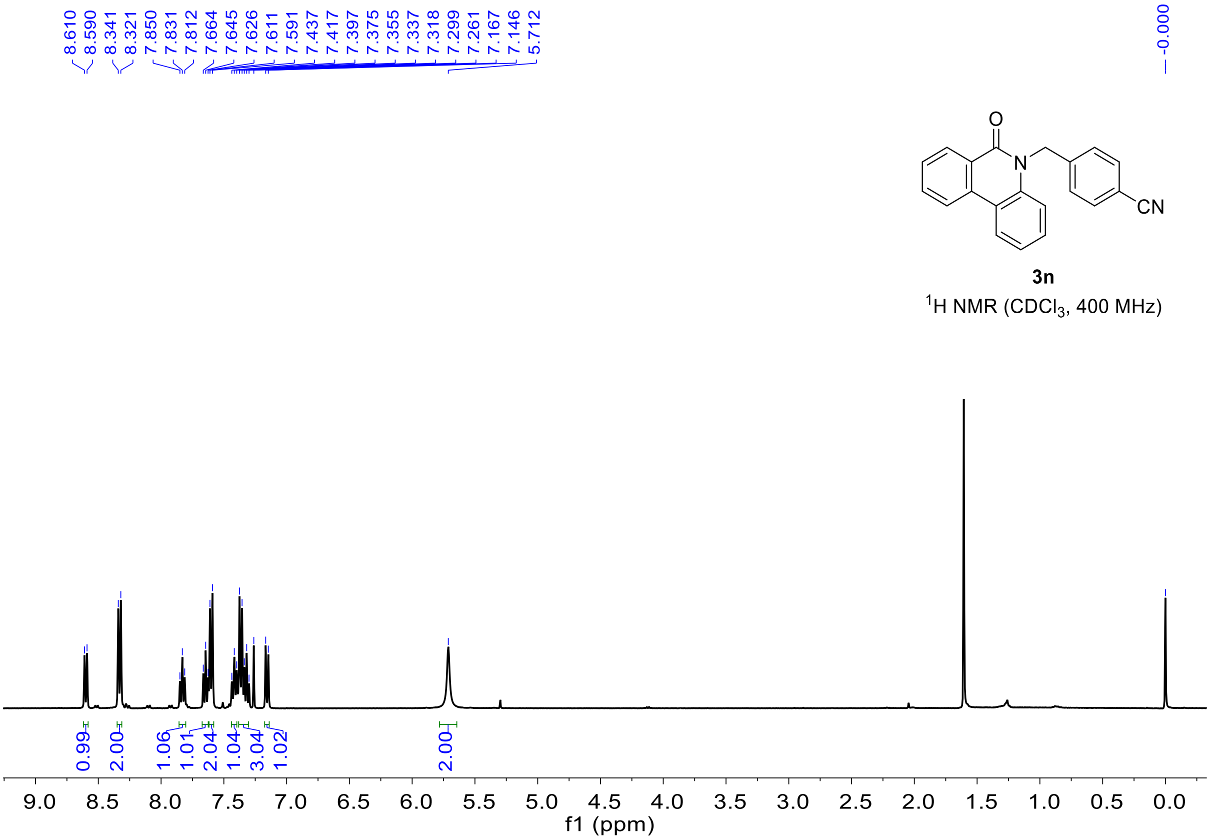

3n

^{1}H NMR ($CDCl_3$, 400 MHz)

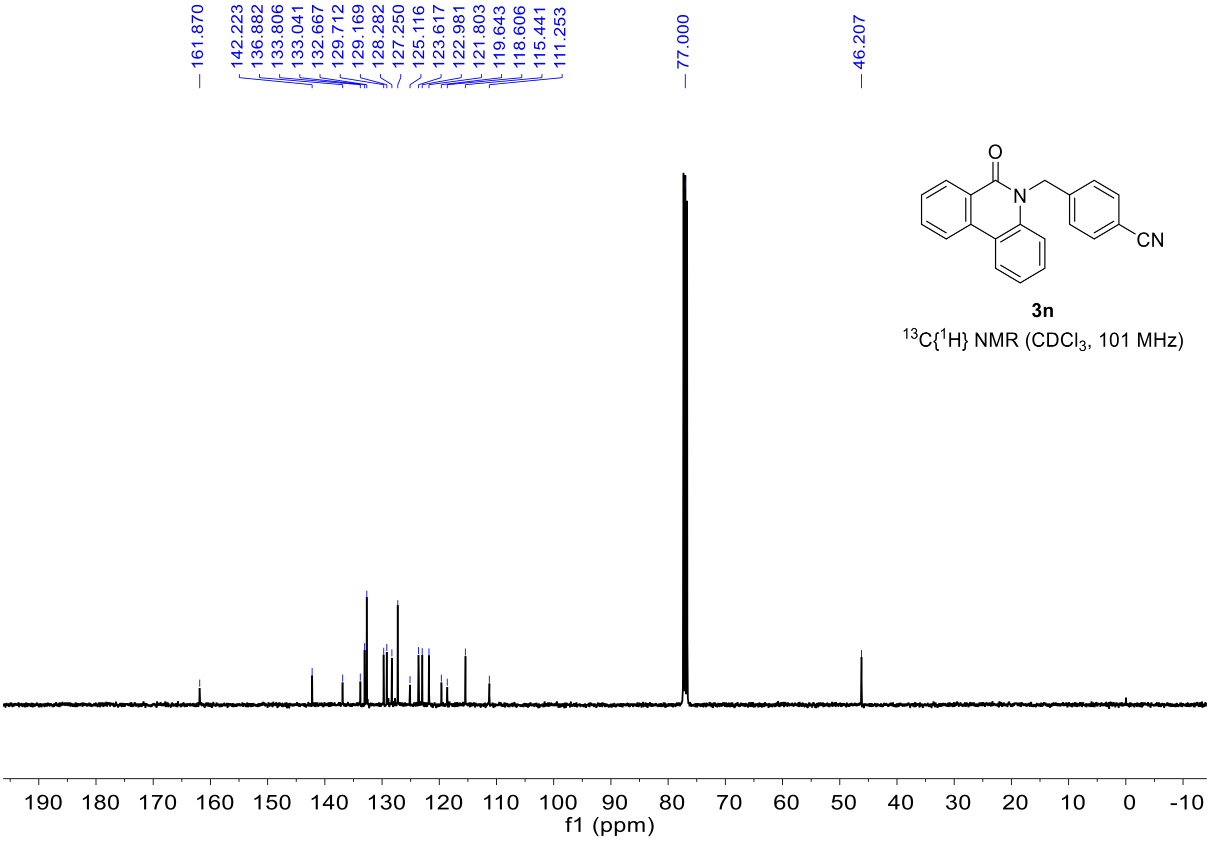

3n

$^{13}C\{^1H\}$ NMR ($CDCl_3$, 101 MHz)

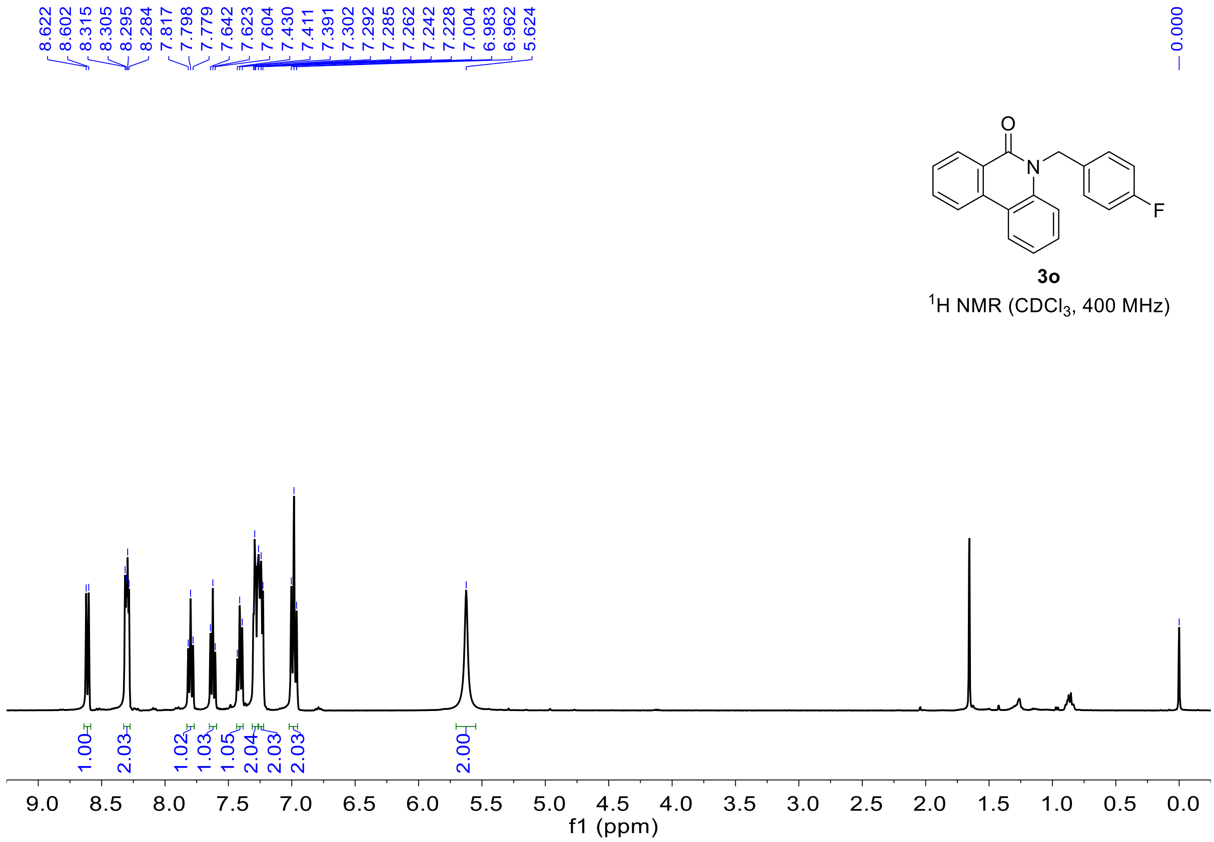
8.622 8.602 8.315 8.305 8.295 8.284 7.817 7.798 7.779 7.642 7.623 7.604 7.430 7.411 7.391 7.302 7.292 7.285 7.262 7.242 7.228 7.004 6.983 6.962 5.624
0.000
O
N
F
3o
^{1}H NMR ($CDCl_3$, 400 MHz)
1.00 2.03 1.02 1.03 1.05 2.04 2.03 2.03 2.00
9.0 8.5 8.0 7.5 7.0 6.5 6.0 5.5 5.0 4.5 4.0 3.5 3.0 2.5 2.0 1.5 1.0 0.5 0.0
f1 (ppm)

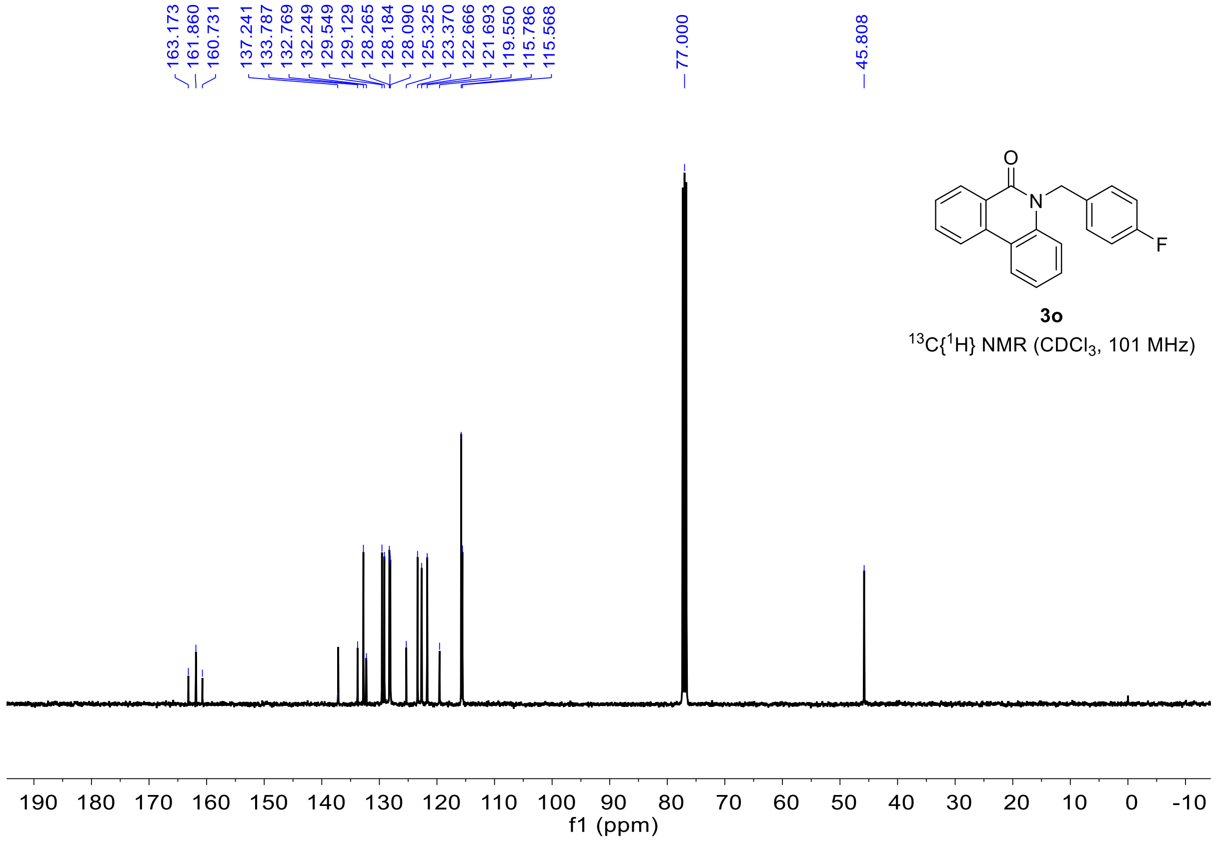
163.173 161.860 160.731 137.241 133.787 132.769 132.249 129.549 129.129 128.265 128.184 128.090 125.325 123.370 122.666 121.693 119.550 115.786 115.568
77.000
45.808
O
N
F
3o
$^{13}C\{^1H\}$ NMR ($CDCl_3$, 101 MHz)
190 180 170 160 150 140 130 120 110 100 90 80 70 60 50 40 30 20 10 0 -10
f1 (ppm)

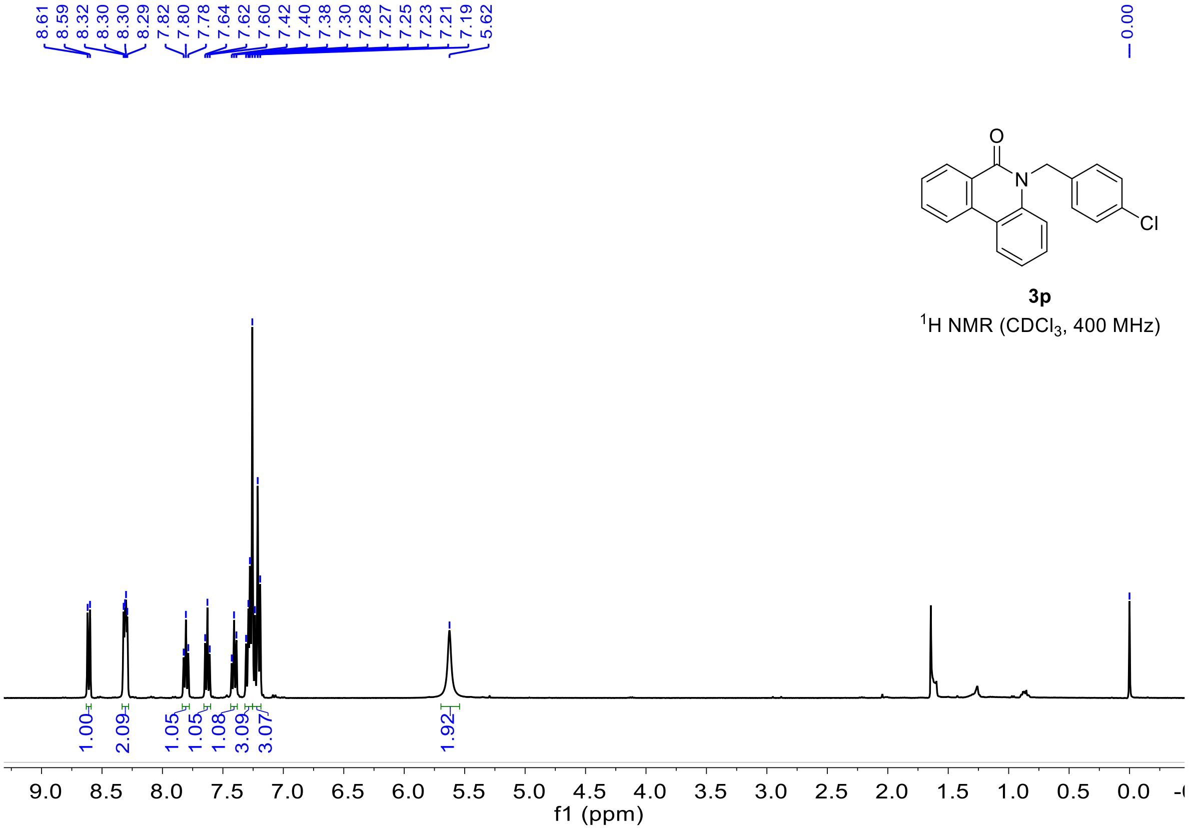

3p

^{1}H NMR ($CDCl_3$, 400 MHz)

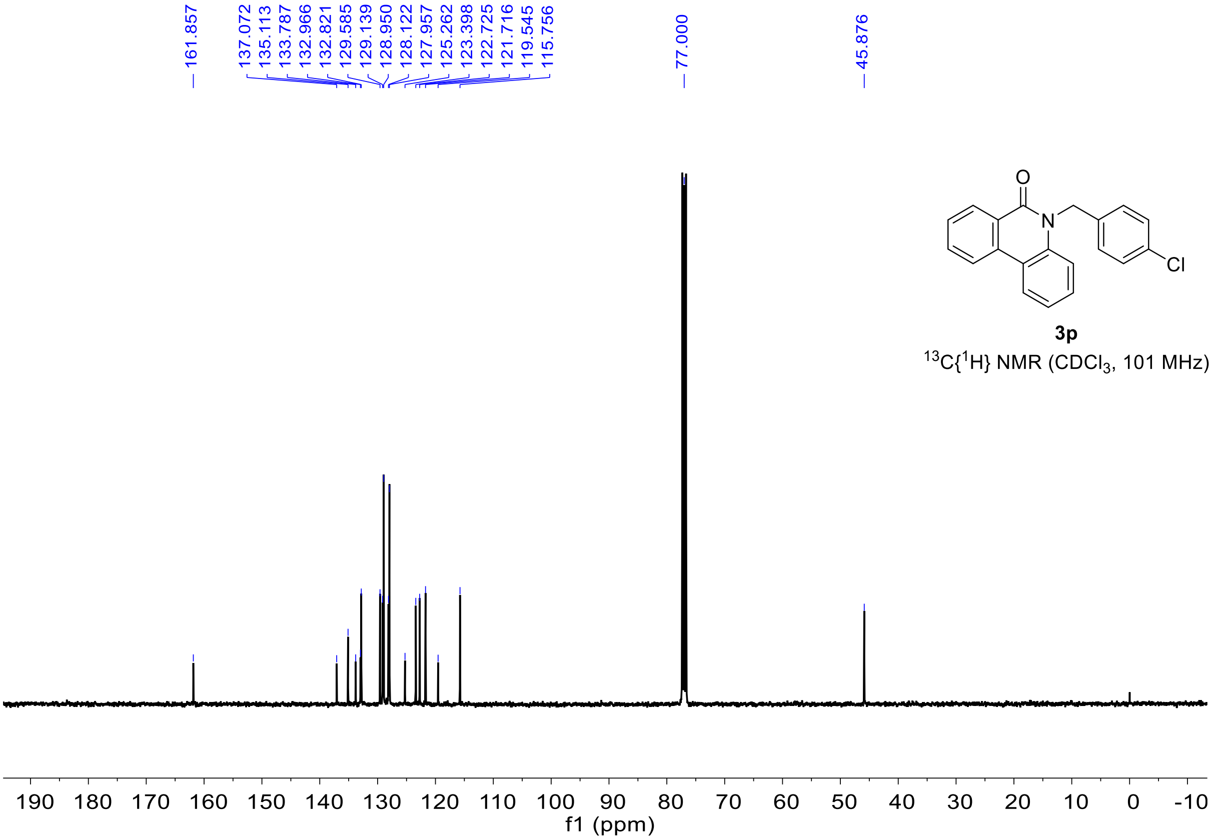

3p

^{13}C{^{1}H} NMR ($CDCl_3$, 101 MHz)

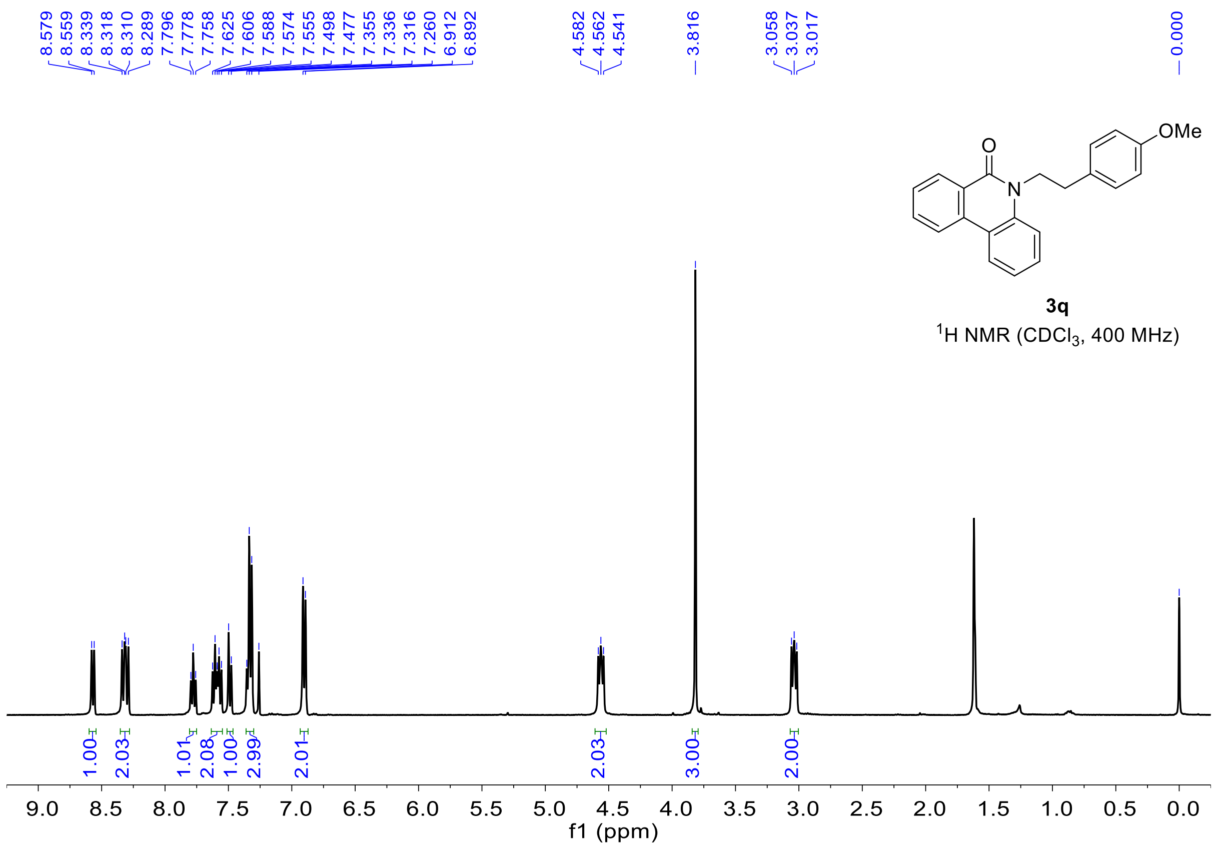
8.579
8.559
8.339
8.318
8.310
8.289
7.796
7.778
7.758
7.625
7.606
7.588
7.574
7.555
7.498
7.477
7.355
7.336
7.316
7.260
6.912
6.892
4.582
4.562
4.541
3.816
3.058
3.037
3.017
0.000
OMe
O
N
3q
^{1}H NMR ($CDCl_3$, 400 MHz)
1.00
2.03
1.01
2.08
1.00
2.99
2.01
2.03
3.00
2.00
9.0 8.5 8.0 7.5 7.0 6.5 6.0 5.5 5.0 4.5 4.0 3.5 3.0 2.5 2.0 1.5 1.0 0.5 0.0
f1 (ppm)

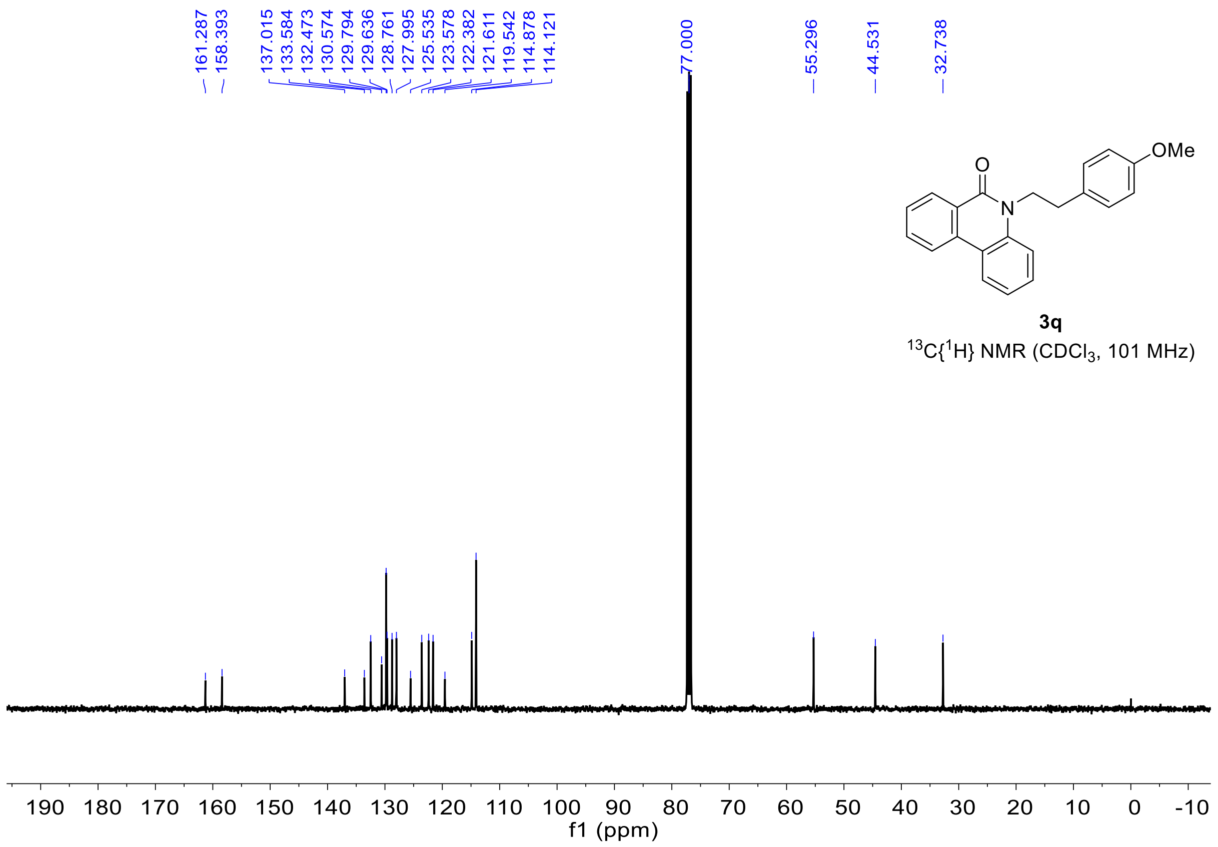
161.287
158.393
137.015
133.584
132.473
130.574
129.794
129.636
128.761
127.995
125.535
123.578
122.382
121.611
119.542
114.878
114.121
77.000
55.296
44.531
32.738
OMe
O
N
3q
$^{13}C\{^{1}H\}$ NMR ($CDCl_3$, 101 MHz)
190 180 170 160 150 140 130 120 110 100 90 80 70 60 50 40 30 20 10 0 -10
f1 (ppm)

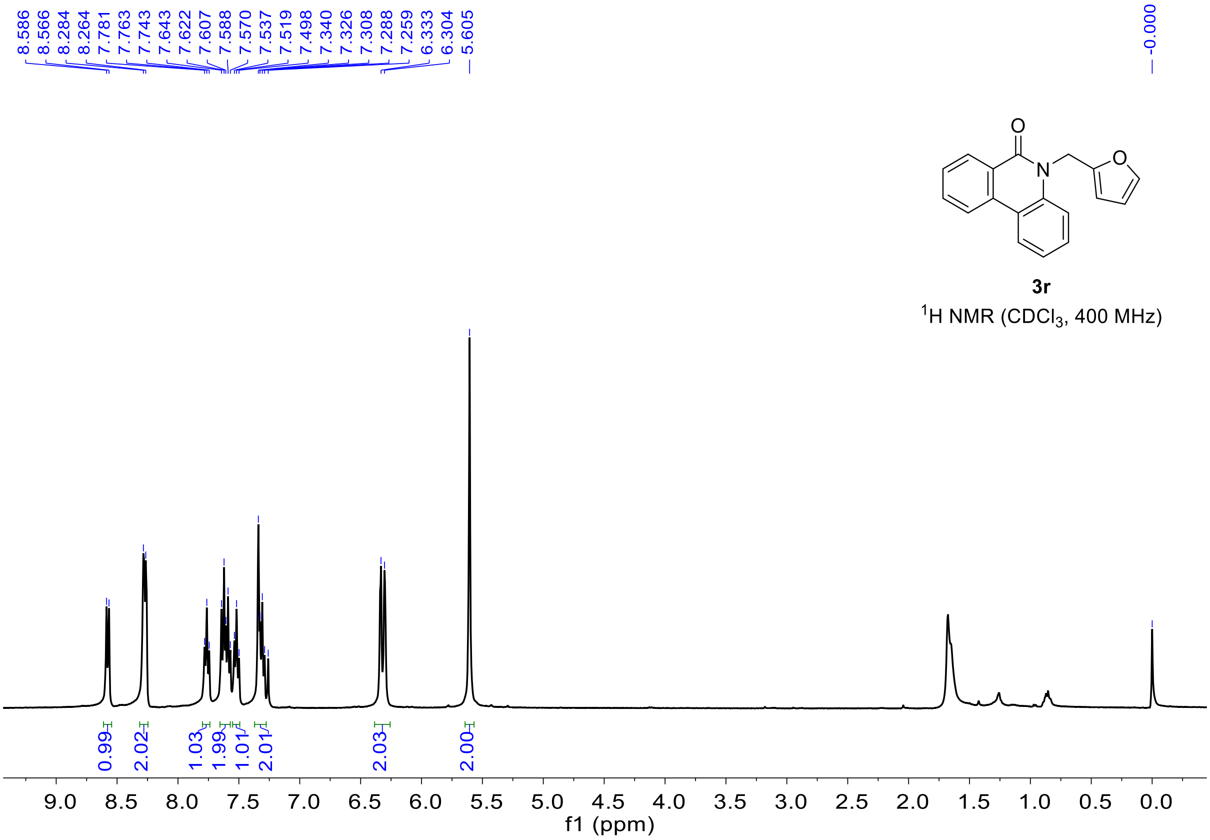

3r

^{1}H NMR ($CDCl_3$, 400 MHz)

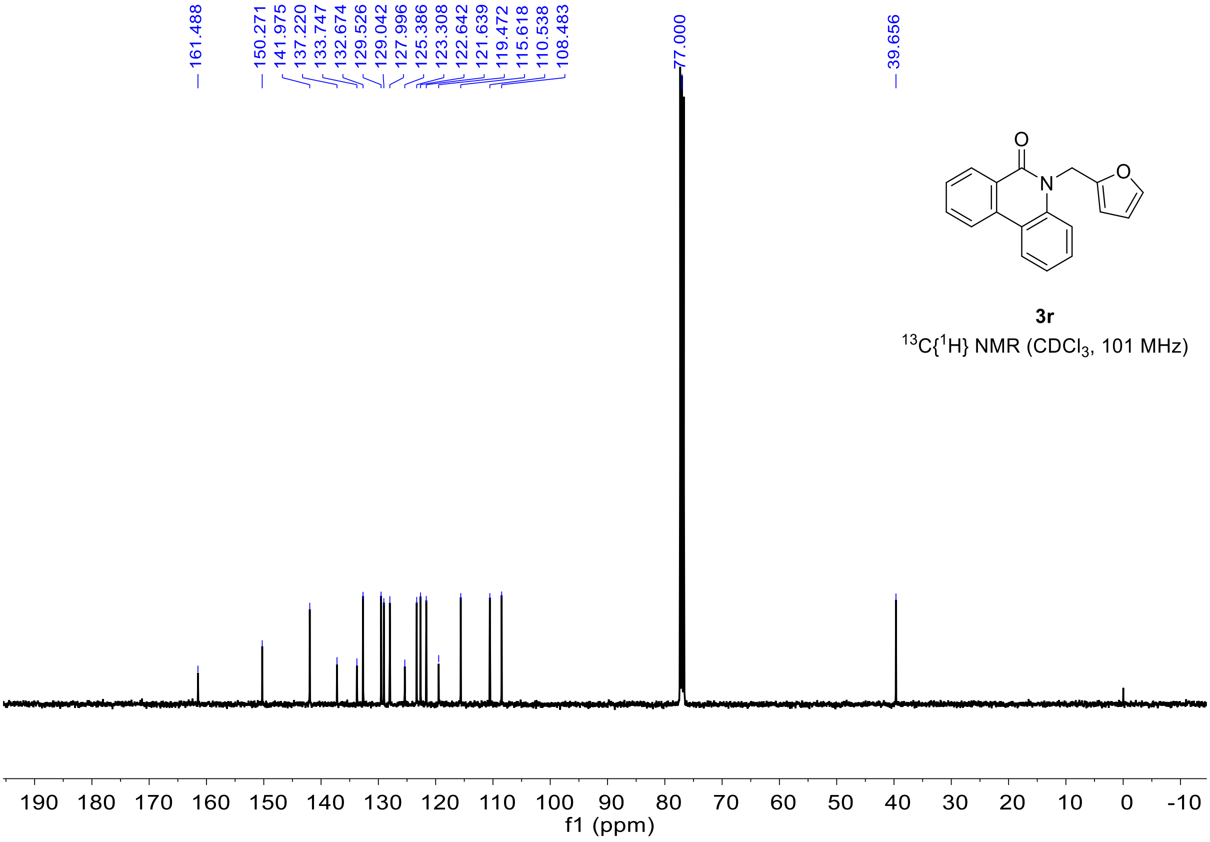

3r

$^{13}C\{^1H\}$ NMR ($CDCl_3$, 101 MHz)

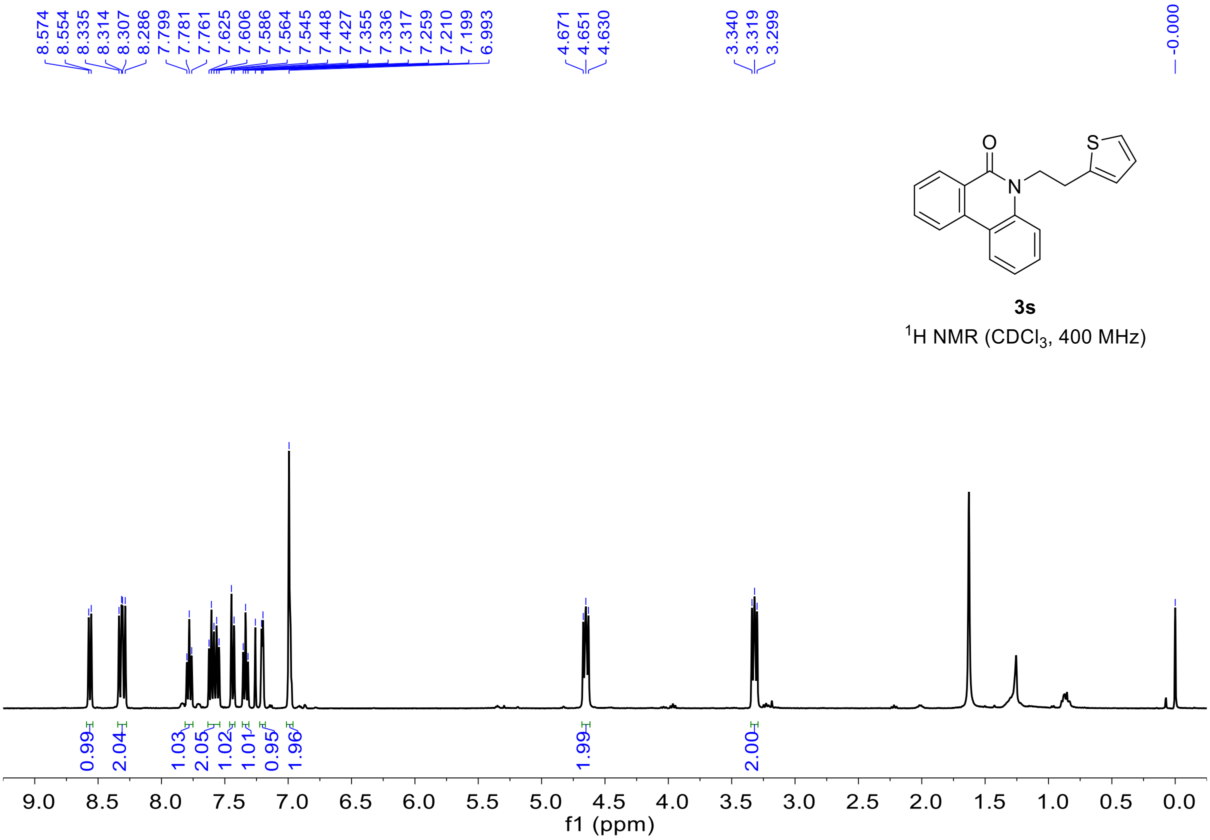
3s
^{1}H NMR (CDCl$_3$, 400 MHz)

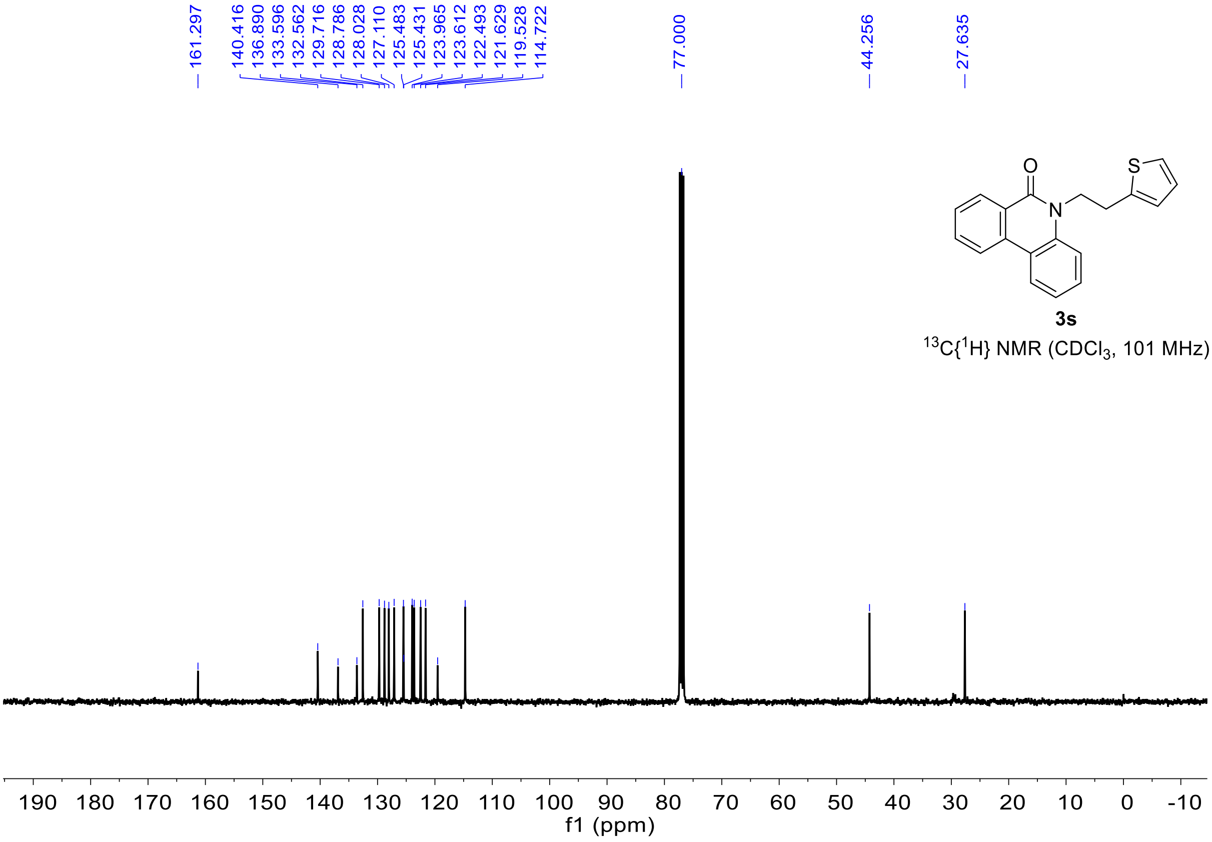
3s
^{13}C{^{1}H} NMR (CDCl$_3$, 101 MHz)

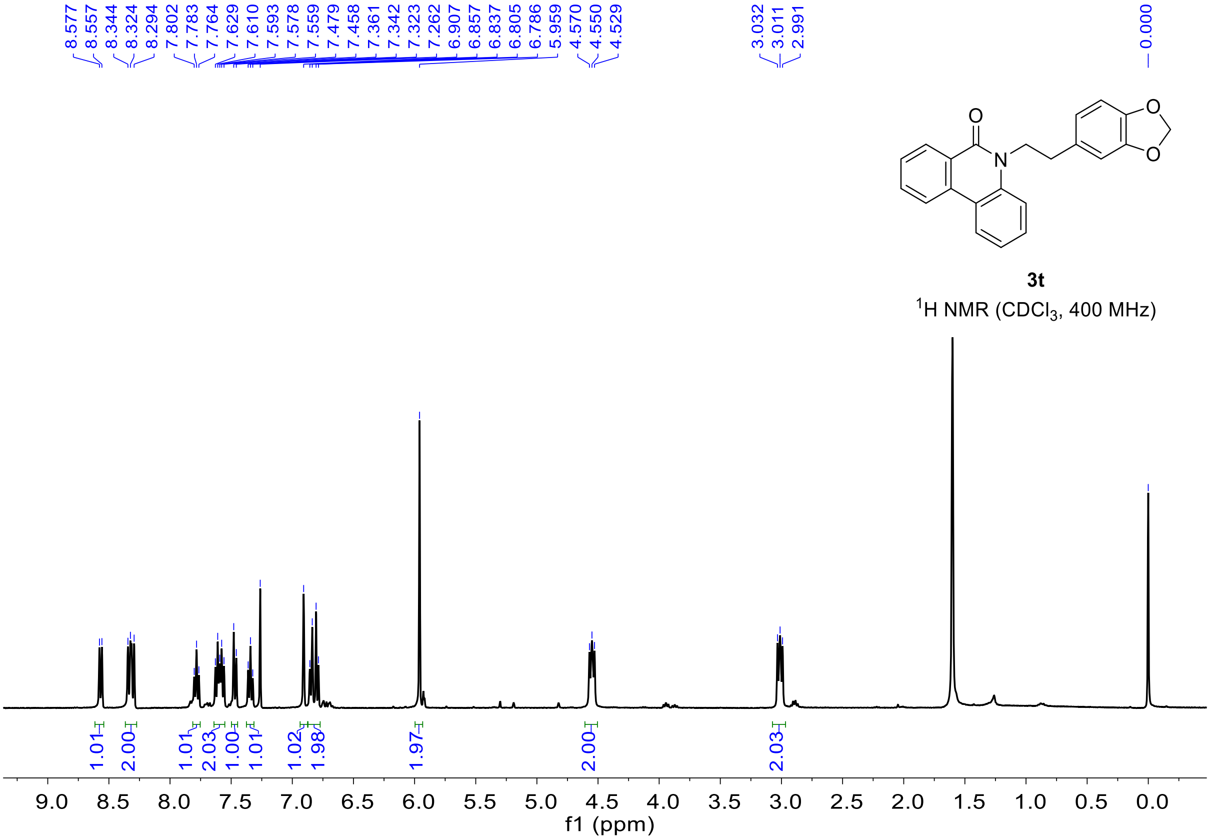
8.577
8.557
8.344
8.324
8.294
7.802
7.783
7.764
7.629
7.610
7.593
7.578
7.559
7.479
7.458
7.361
7.342
7.323
7.262
6.907
6.857
6.837
6.805
6.786
5.959
4.570
4.550
4.529
3.032
3.011
2.991
0.000
O
N
O
O
3t
1H NMR (CDCl3, 400 MHz)
1.01
2.00
1.01
2.03
1.00
1.01
1.02
1.98
1.97
2.00
2.03
9.0 8.5 8.0 7.5 7.0 6.5 6.0 5.5 5.0 4.5 4.0 3.5 3.0 2.5 2.0 1.5 1.0 0.5 0.0
f1 (ppm)

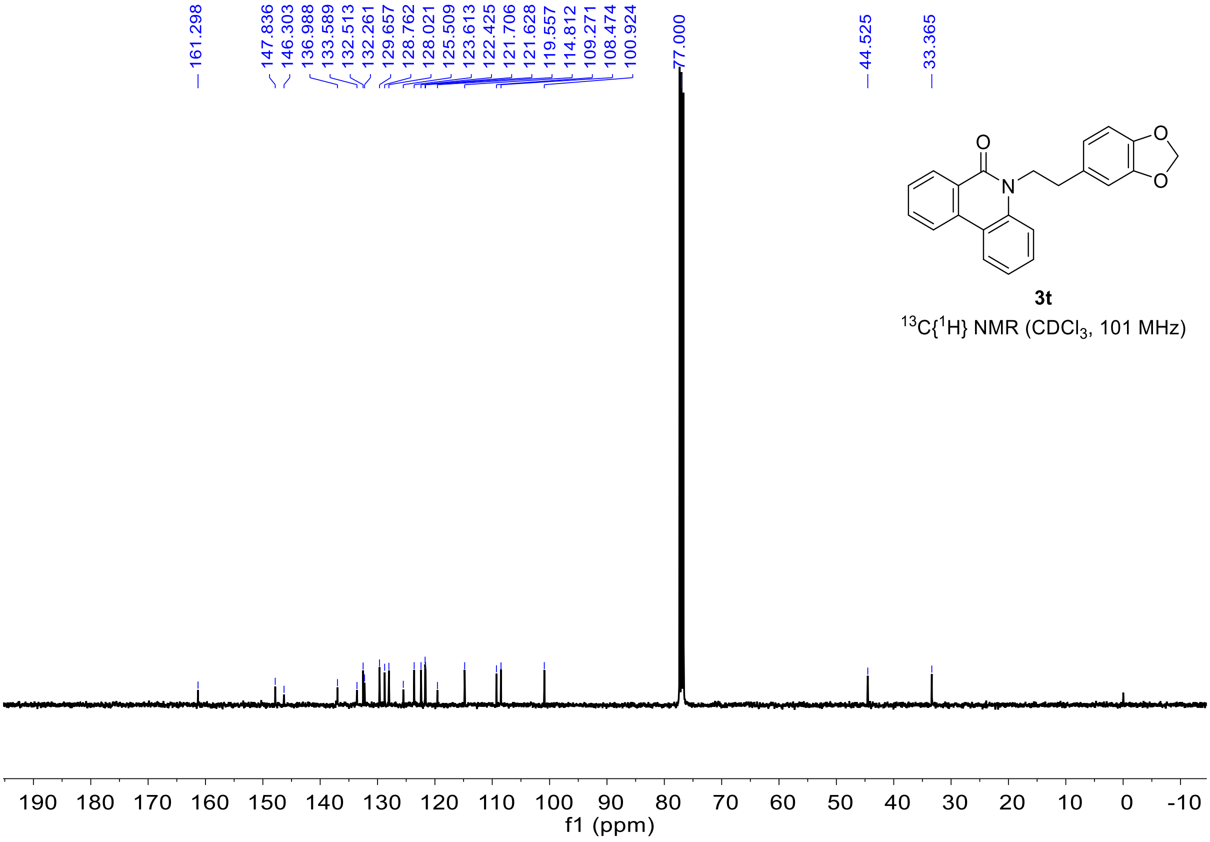
161.298
147.836
146.303
136.988
133.589
132.513
132.261
129.657
128.762
128.021
125.509
123.613
122.425
121.706
121.628
119.557
114.812
109.271
108.474
100.924
77.000
44.525
33.365
O
N
O
O
3t
13C{1H} NMR (CDCl3, 101 MHz)
190 180 170 160 150 140 130 120 110 100 90 80 70 60 50 40 30 20 10 0 -10
f1 (ppm)

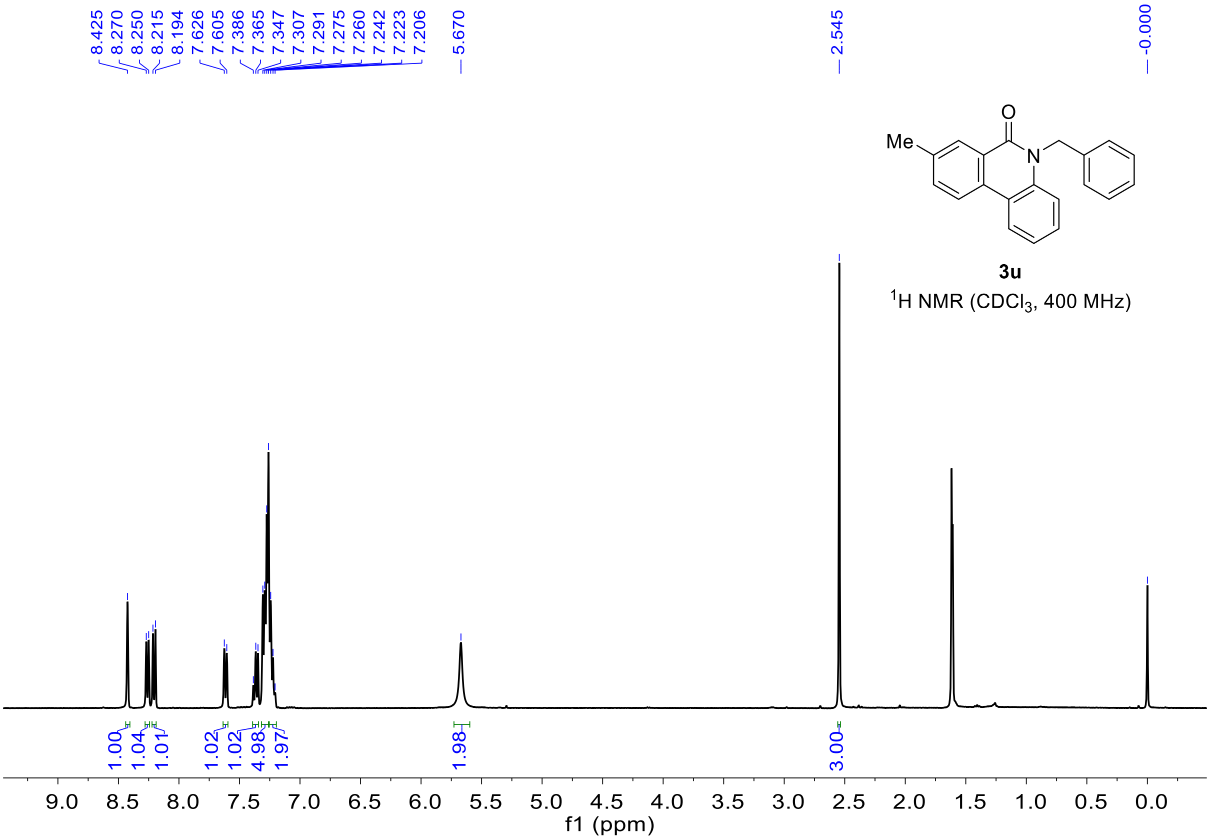
8.425
8.270
8.250
8.215
8.194
7.626
7.605
7.386
7.365
7.347
7.307
7.291
7.275
7.260
7.242
7.223
7.206
5.670
2.545
-0.000
Me
O
N
3u
1H NMR (CDCl3, 400 MHz)
1.00
1.04
1.01
1.02
1.02
4.98
1.97
1.98
3.00
9.0 8.5 8.0 7.5 7.0 6.5 6.0 5.5 5.0 4.5 4.0 3.5 3.0 2.5 2.0 1.5 1.0 0.5 0.0
f1 (ppm)

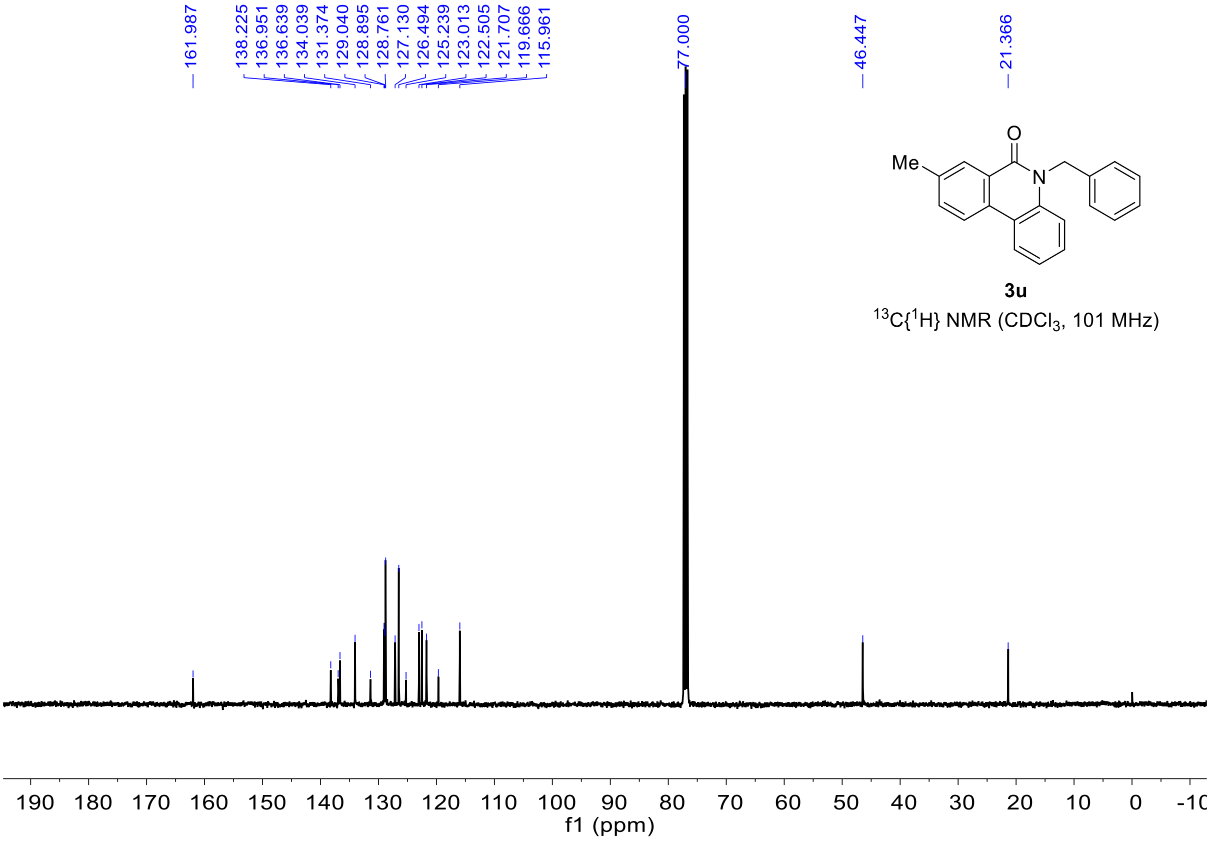
161.987
138.225
136.951
136.639
134.039
131.374
129.040
128.895
128.761
127.130
126.494
125.239
123.013
122.505
121.707
119.666
115.961
77.000
46.447
21.366
Me
O
N
3u
13C{1H} NMR (CDCl3, 101 MHz)
190 180 170 160 150 140 130 120 110 100 90 80 70 60 50 40 30 20 10 0 -10
f1 (ppm)

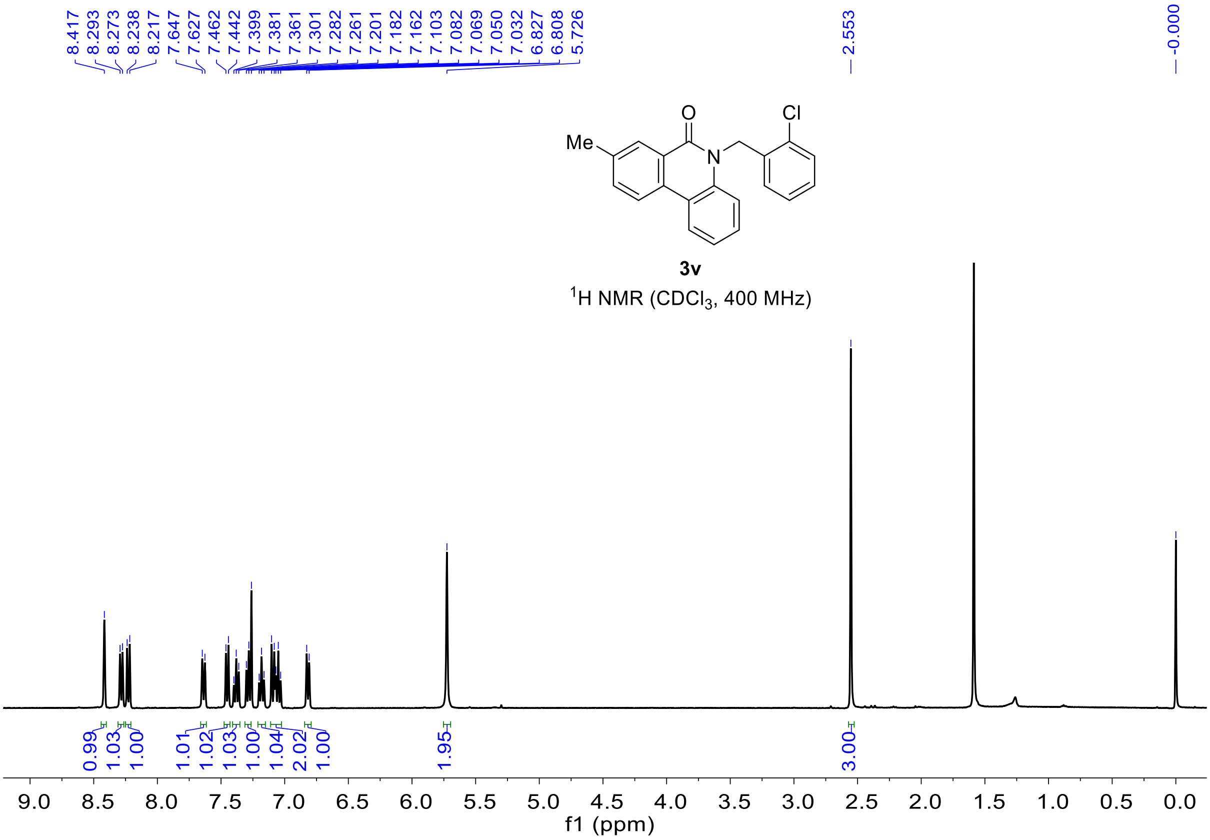
8.417
8.293
8.273
8.238
8.217
7.647
7.627
7.462
7.442
7.399
7.381
7.361
7.301
7.282
7.261
7.201
7.182
7.162
7.103
7.082
7.069
7.050
7.032
6.827
6.808
5.726
2.553
-0.000
O
Cl
Me
N
3v
^{1}H NMR (CDCl$_3$, 400 MHz)
0.99
1.03
1.00
1.01
1.02
1.03
1.00
1.04
2.02
1.00
1.95
3.00
9.0 8.5 8.0 7.5 7.0 6.5 6.0 5.5 5.0 4.5 4.0 3.5 3.0 2.5 2.0 1.5 1.0 0.5 0.0
f1 (ppm)

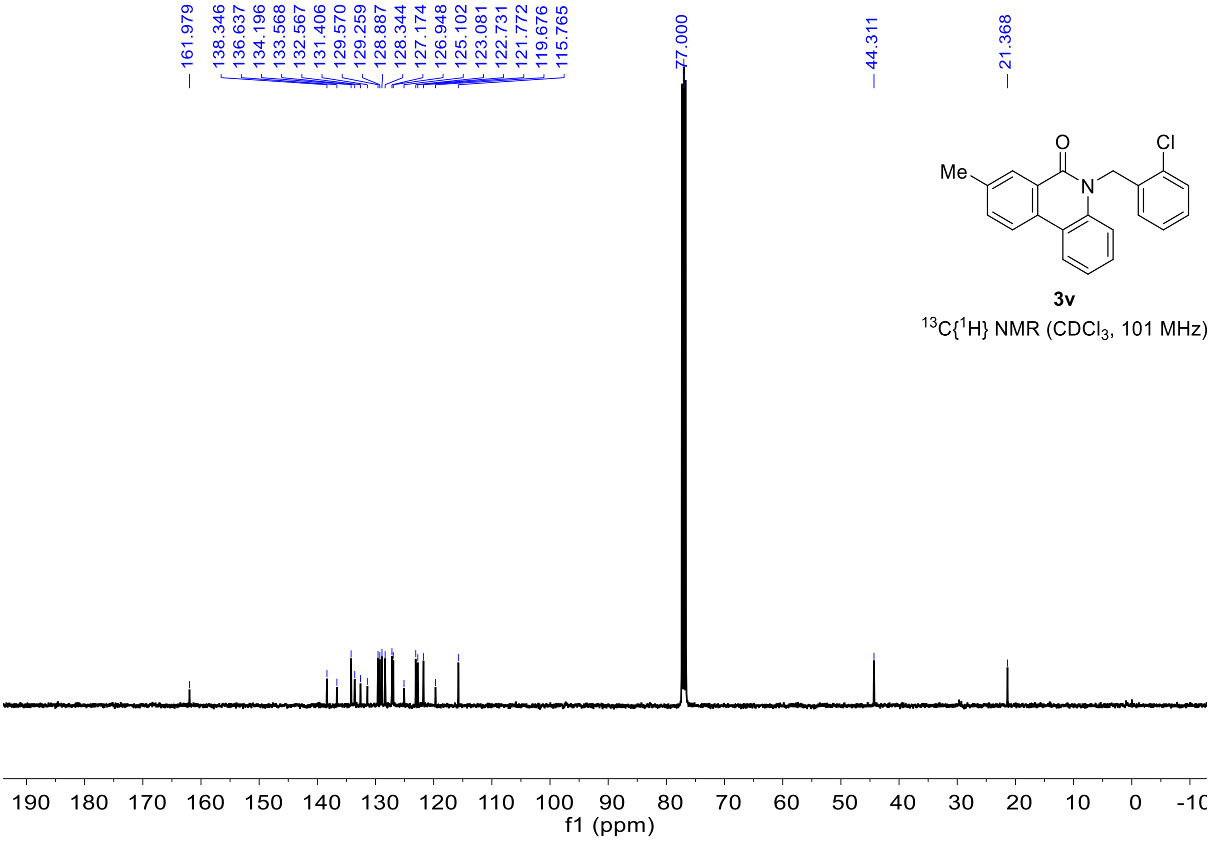
161.979
138.346
136.637
134.196
133.568
132.567
131.406
129.570
129.259
128.887
128.344
127.174
126.948
125.102
123.081
122.731
121.772
119.676
115.765
77.000
44.311
21.368
O
Cl
Me
N
3v
^{13}C{^{1}H} NMR (CDCl$_3$, 101 MHz)
190 180 170 160 150 140 130 120 110 100 90 80 70 60 50 40 30 20 10 0 -10
f1 (ppm)

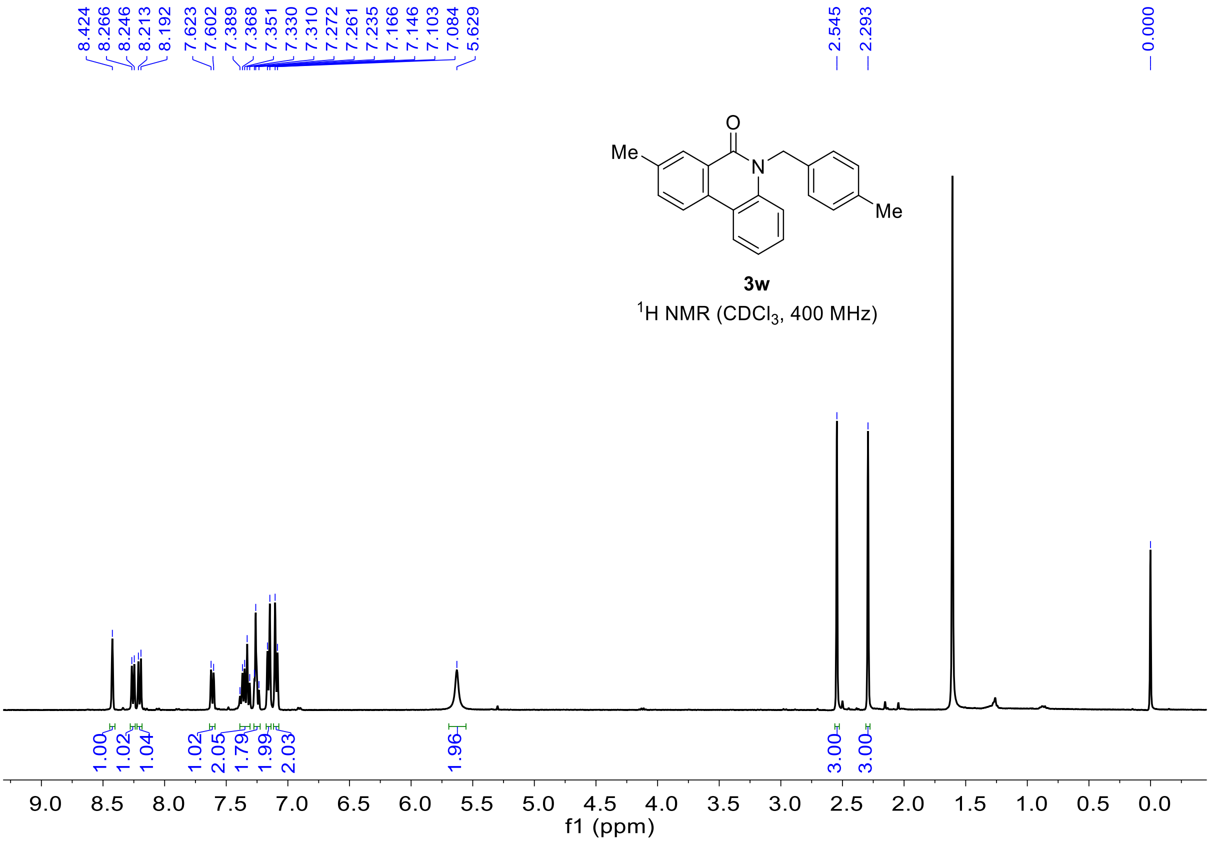

3w

^{1}H NMR ($CDCl_3$, 400 MHz)

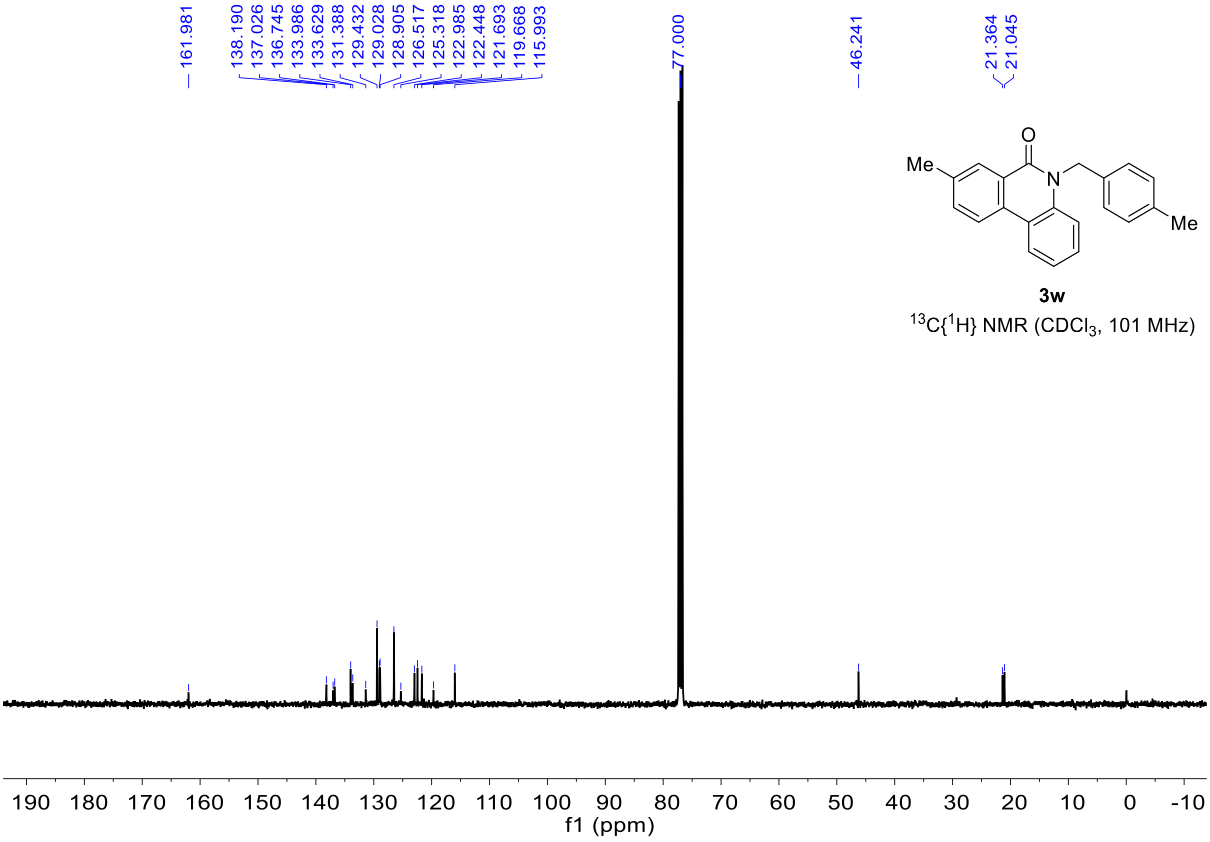

3w

$^{13}C\{^{1}H\}$ NMR ($CDCl_3$, 101 MHz)

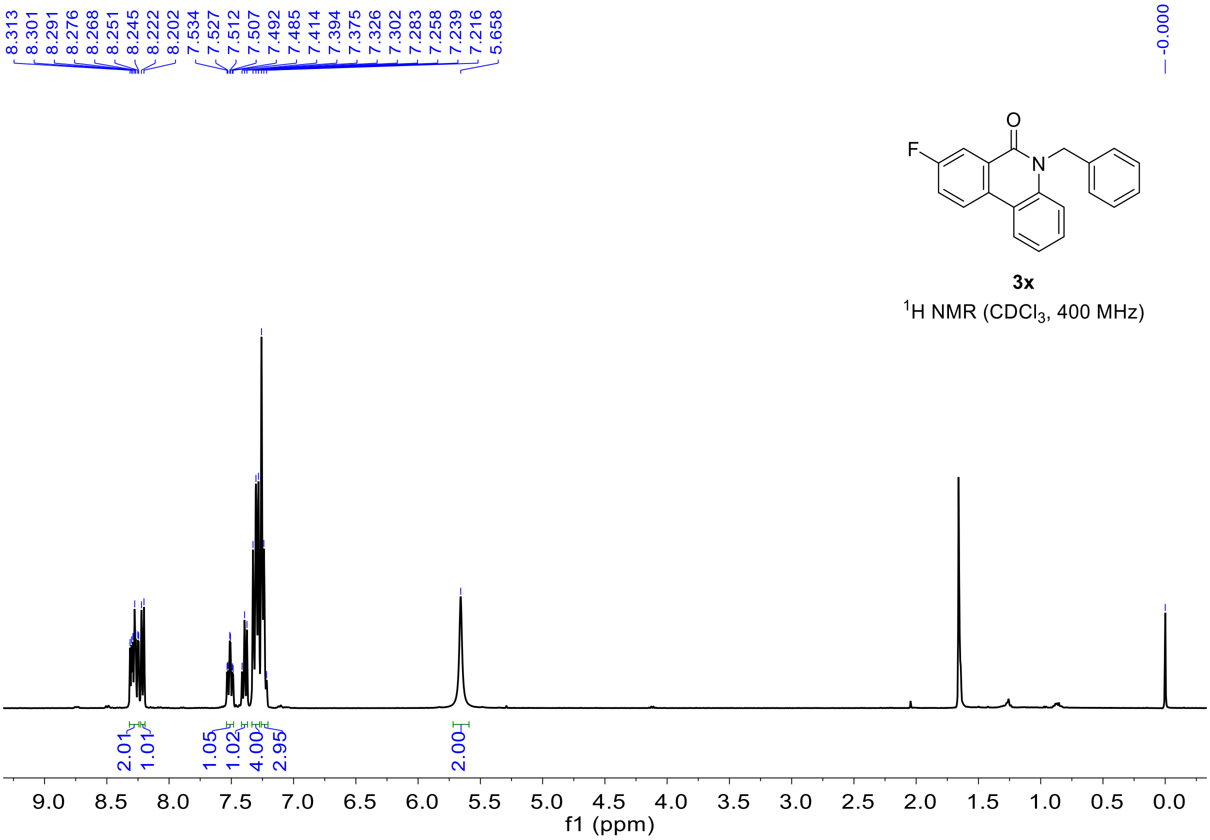
8.313
8.301
8.291
8.276
8.268
8.251
8.245
8.222
8.202
7.534
7.527
7.512
7.507
7.492
7.485
7.414
7.394
7.375
7.326
7.302
7.283
7.258
7.239
7.216
5.658
-0.000
F
O
N
3x
^{1}H NMR (CDCl$_3$, 400 MHz)
2.01
1.01
1.05
1.02
4.00
2.95
2.00
9.0 8.5 8.0 7.5 7.0 6.5 6.0 5.5 5.0 4.5 4.0 3.5 3.0 2.5 2.0 1.5 1.0 0.5 0.0
f1 (ppm)

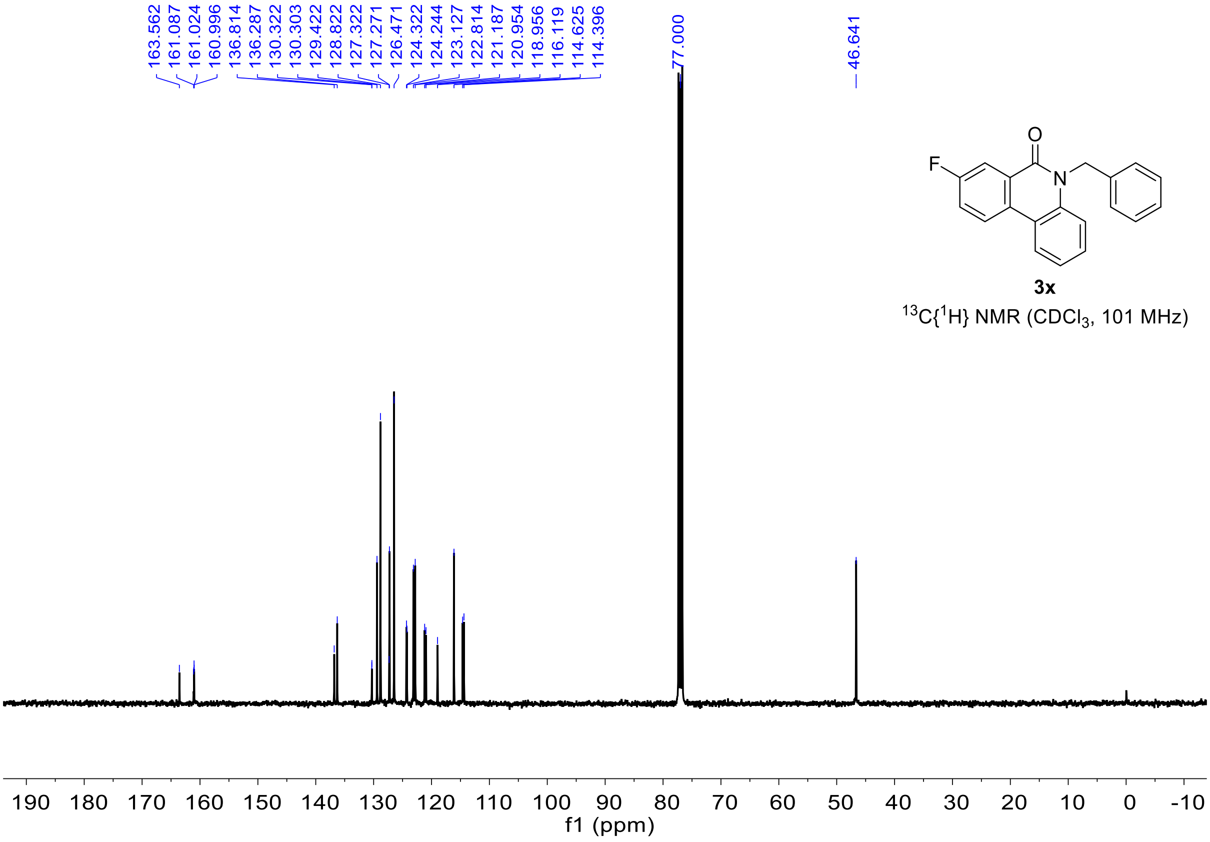
163.562
161.087
161.024
160.996
136.814
136.287
130.322
130.303
129.422
128.822
127.322
127.271
126.471
124.322
124.244
123.127
122.814
121.187
120.954
118.956
116.119
114.625
114.396
77.000
46.641
F
O
N
3x
^{13}C{^{1}H} NMR (CDCl$_3$, 101 MHz)
190 180 170 160 150 140 130 120 110 100 90 80 70 60 50 40 30 20 10 0 -10
f1 (ppm)

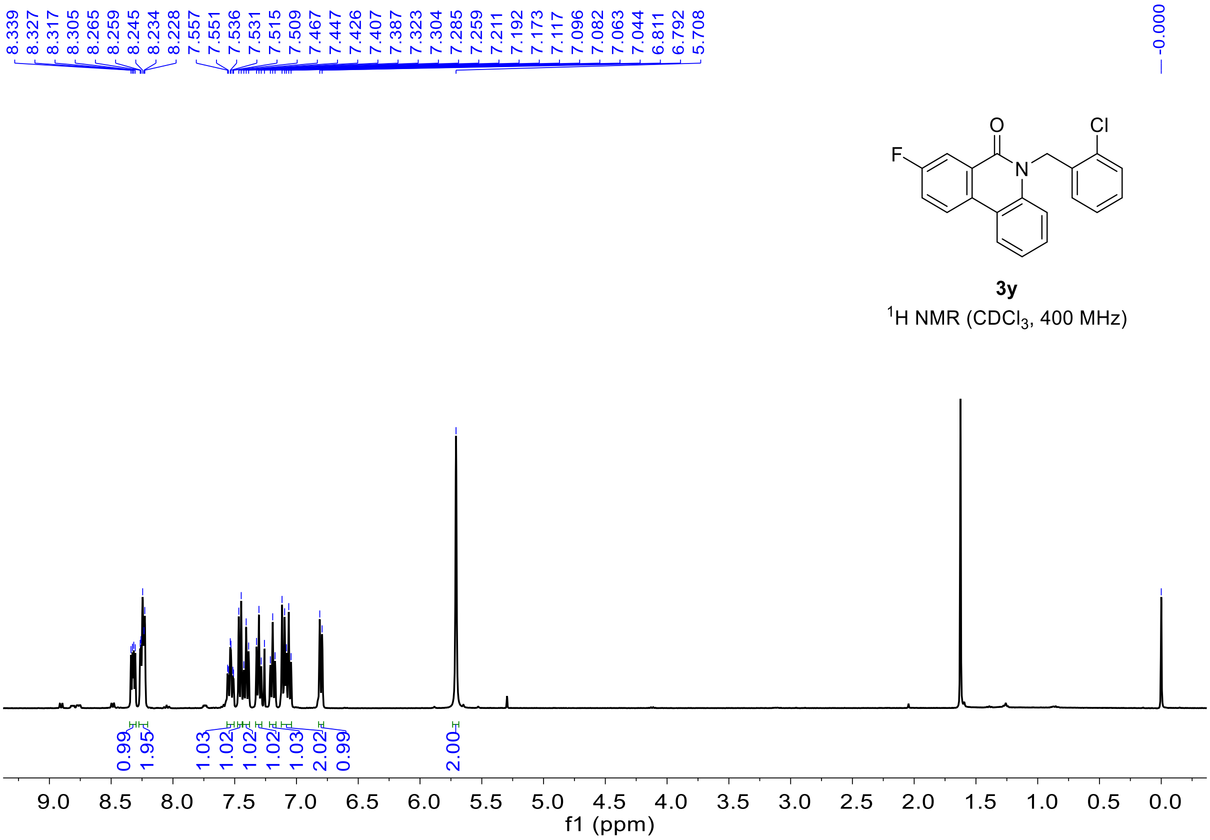
8.339
8.327
8.317
8.305
8.265
8.259
8.245
8.234
8.228
7.557
7.551
7.536
7.531
7.515
7.509
7.467
7.447
7.426
7.407
7.387
7.323
7.304
7.285
7.259
7.211
7.192
7.173
7.117
7.096
7.082
7.063
7.044
6.811
6.792
5.708
-0.000
F
O
N
Cl
3y
^{1}H NMR (CDCl$_3$, 400 MHz)
0.99
1.95
1.03
1.02
1.02
1.02
1.03
2.02
0.99
2.00
9.0 8.5 8.0 7.5 7.0 6.5 6.0 5.5 5.0 4.5 4.0 3.5 3.0 2.5 2.0 1.5 1.0 0.5 0.0
f1 (ppm)

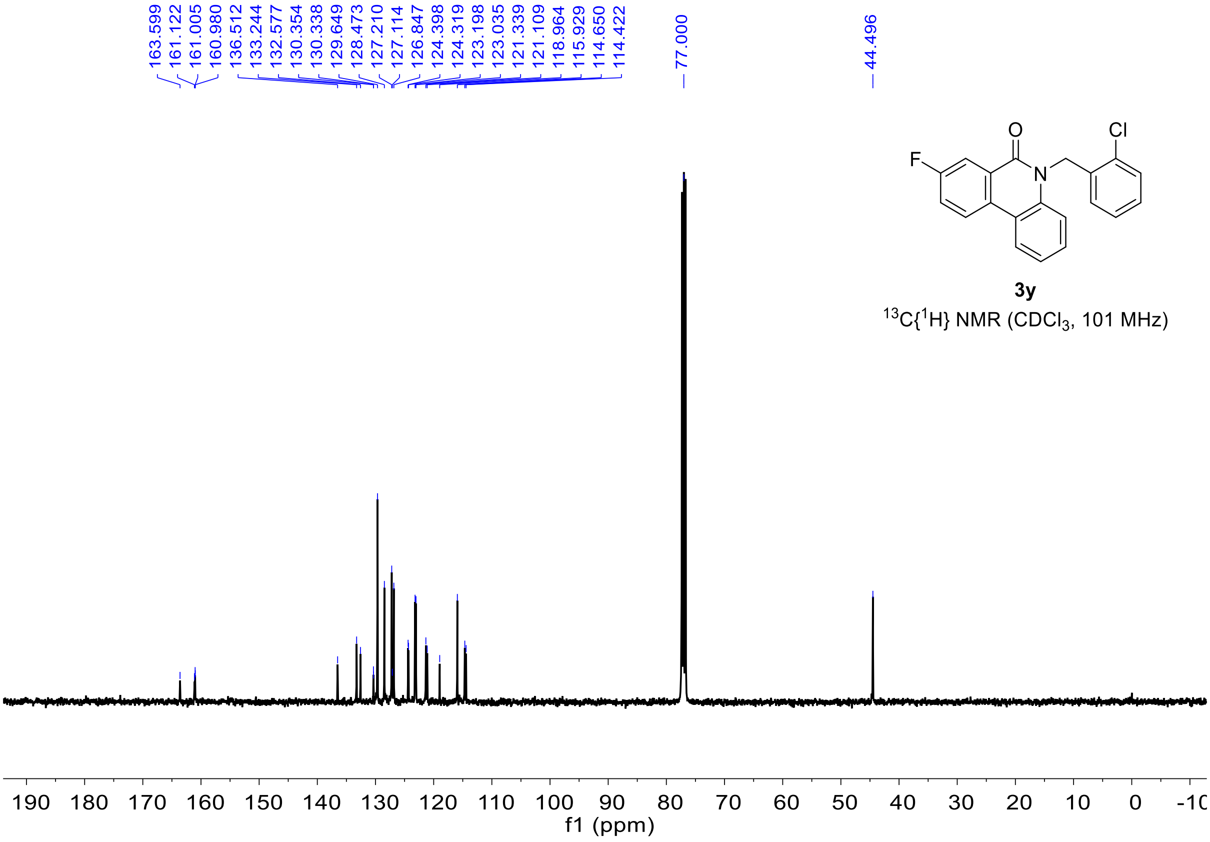
163.599
161.122
161.005
160.980
136.512
133.244
132.577
130.354
130.338
129.649
128.473
127.210
127.114
126.847
124.398
124.319
123.198
123.035
121.339
121.109
118.964
115.929
114.650
114.422
77.000
44.496
F
O
N
Cl
3y
^{13}C{^{1}H} NMR (CDCl$_3$, 101 MHz)
190 180 170 160 150 140 130 120 110 100 90 80 70 60 50 40 30 20 10 0 -10
f1 (ppm)

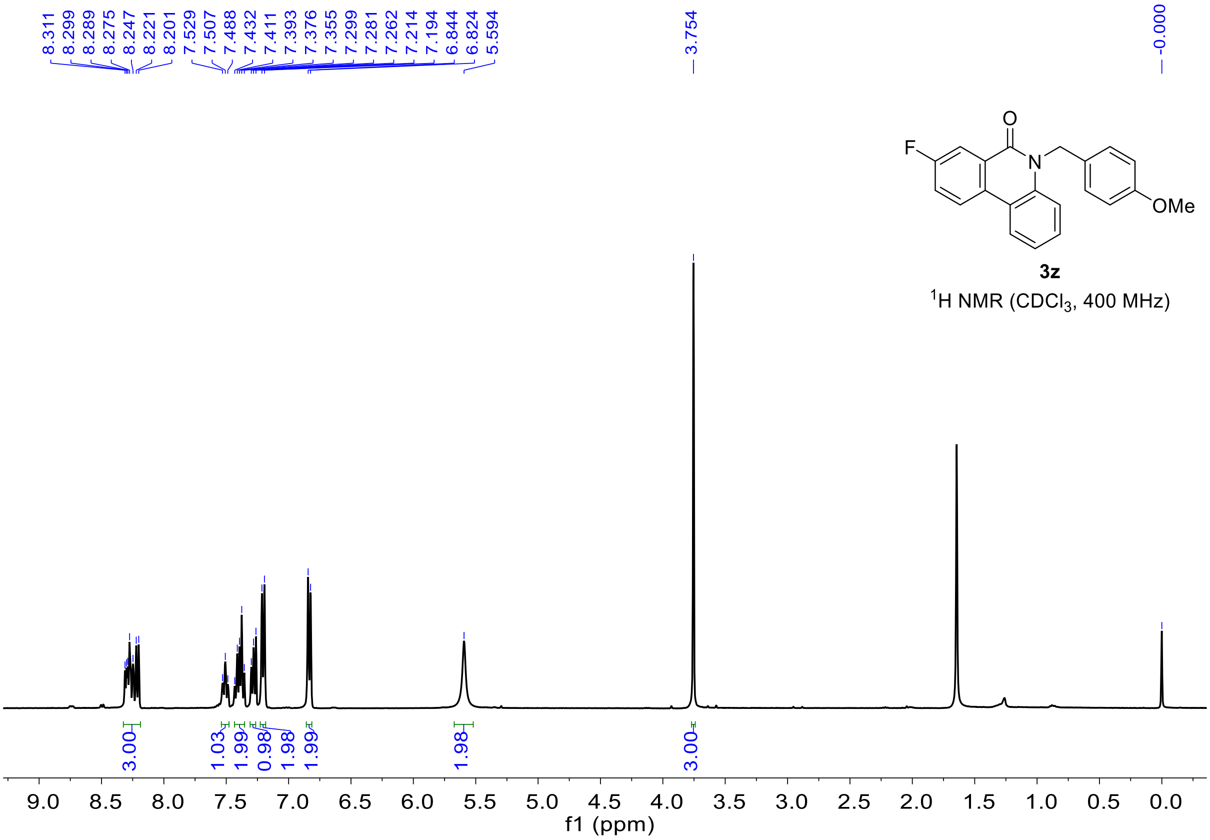
8.311
8.299
8.289
8.275
8.247
8.221
8.201
7.529
7.507
7.488
7.432
7.411
7.393
7.376
7.355
7.299
7.281
7.262
7.214
7.194
6.844
6.824
5.594
3.754
-0.000
F
O
N
OMe
3z
1H NMR (CDCl3, 400 MHz)
3.00
1.03
1.99
0.98
1.98
1.99
1.98
3.00
9.0 8.5 8.0 7.5 7.0 6.5 6.0 5.5 5.0 4.5 4.0 3.5 3.0 2.5 2.0 1.5 1.0 0.5 0.0
f1 (ppm)

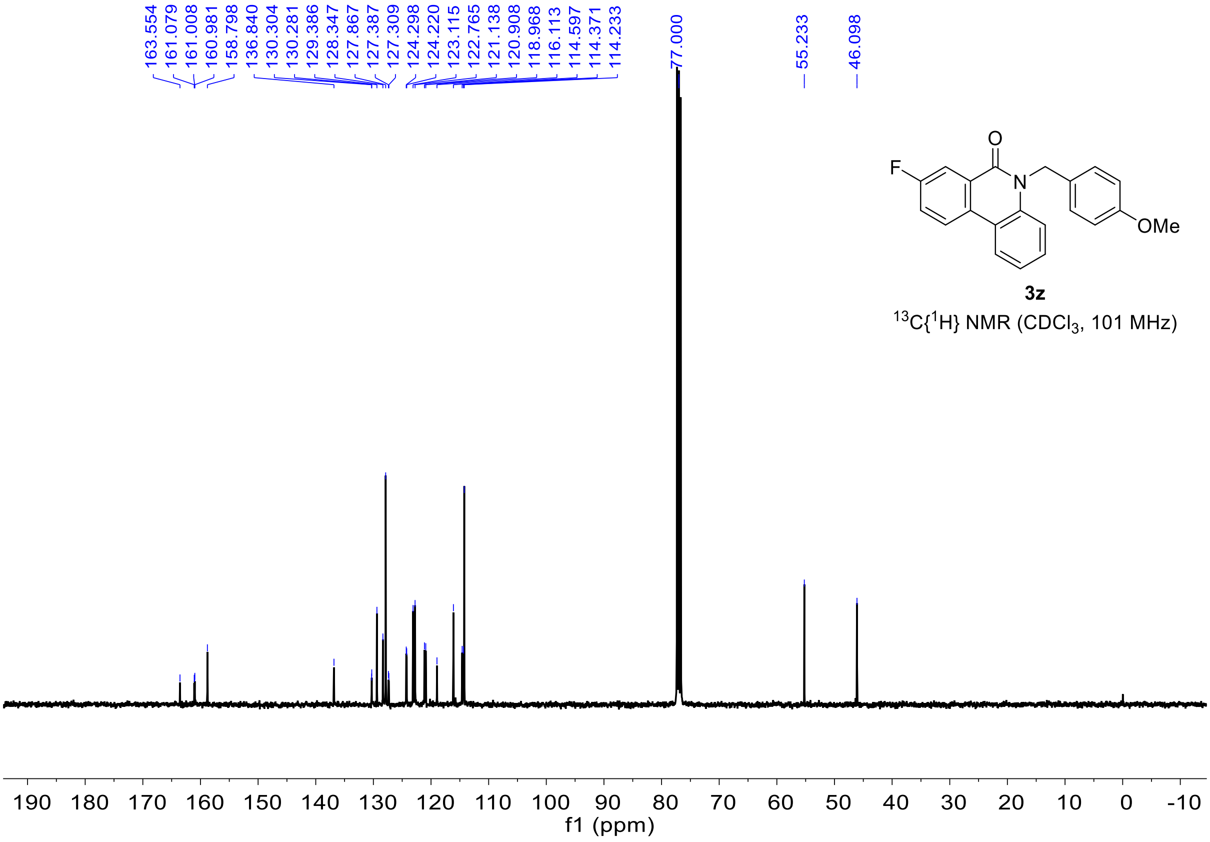
163.554
161.079
161.008
160.981
158.798
136.840
130.304
130.281
129.386
128.347
127.867
127.387
127.309
124.298
124.220
123.115
122.765
121.138
120.908
118.968
116.113
114.597
114.371
114.233
77.000
55.233
46.098
F
O
N
OMe
3z
13C{1H} NMR (CDCl3, 101 MHz)
190 180 170 160 150 140 130 120 110 100 90 80 70 60 50 40 30 20 10 0 -10
f1 (ppm)

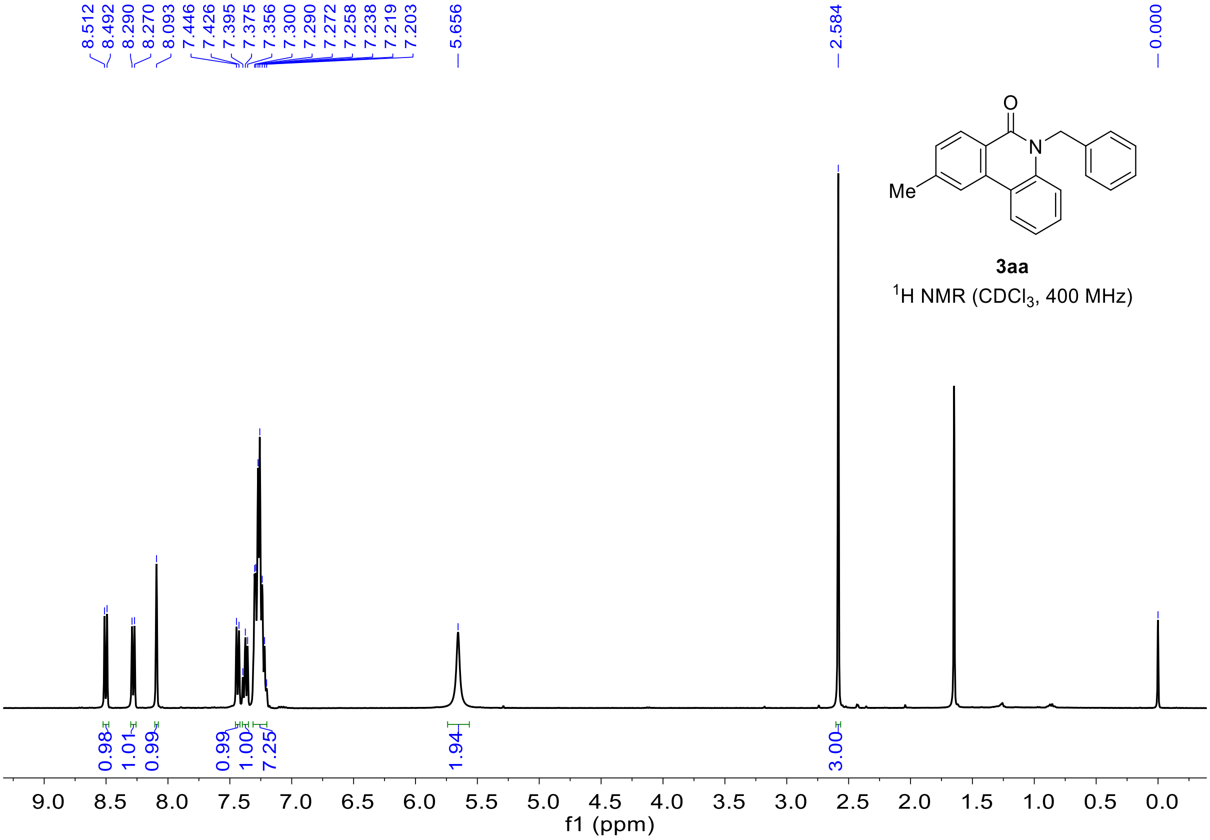

3aa

^{1}H NMR ($CDCl_3$, 400 MHz)

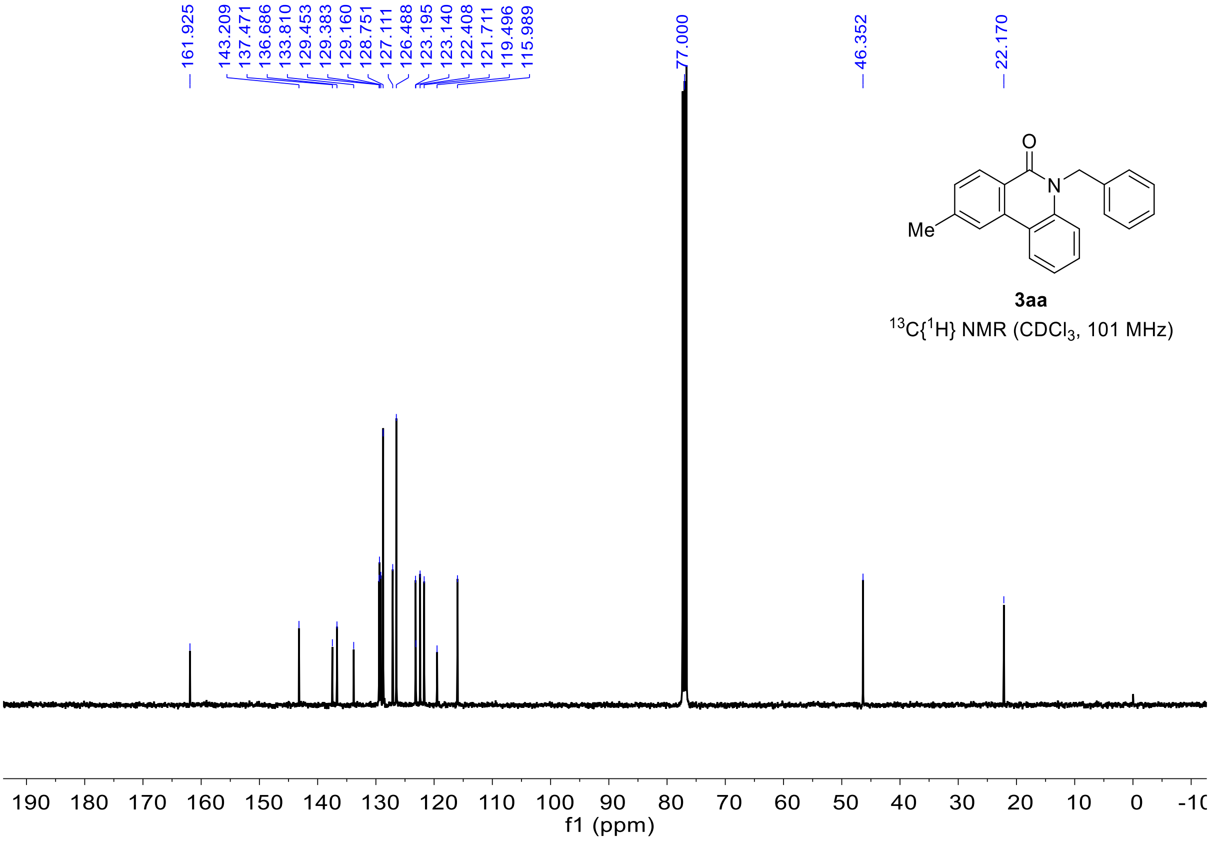

3aa

$^{13}C\{^1H\}$ NMR ($CDCl_3$, 101 MHz)

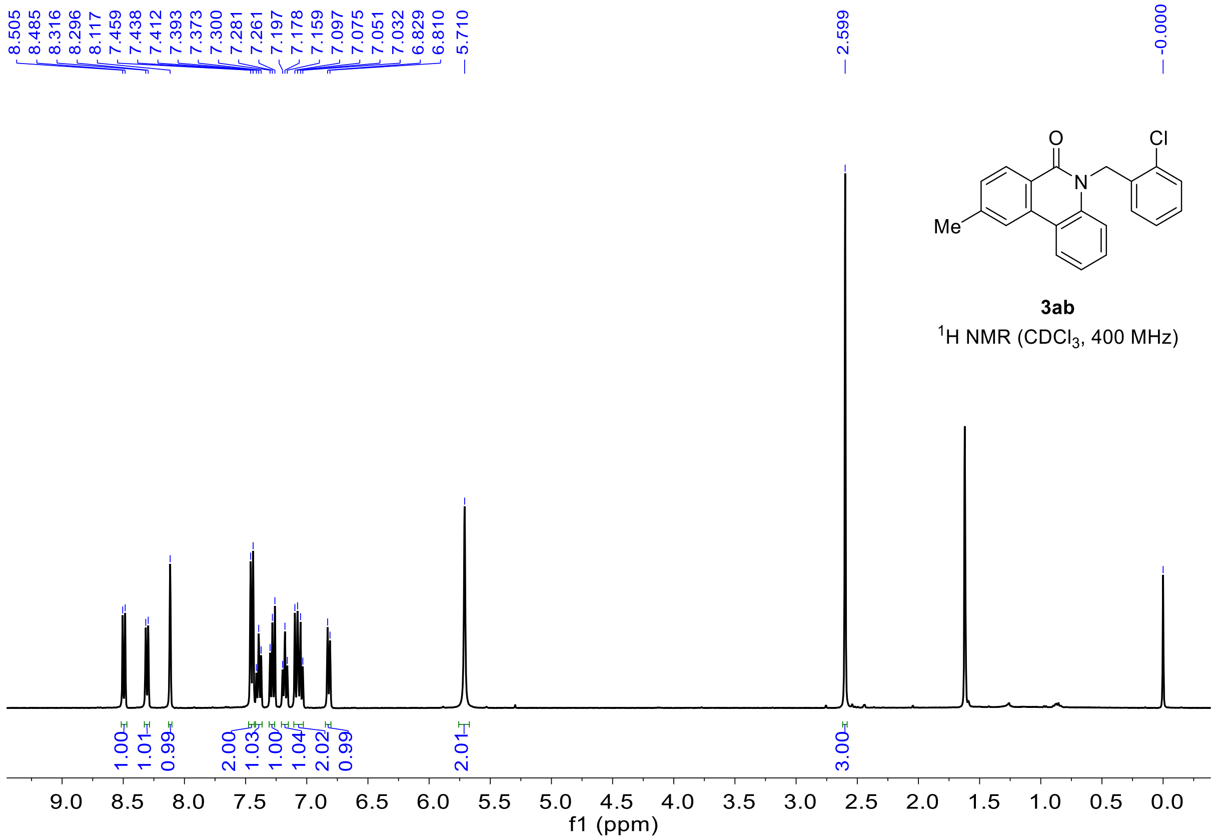

3ab

^{1}H NMR ($CDCl_3$, 400 MHz)

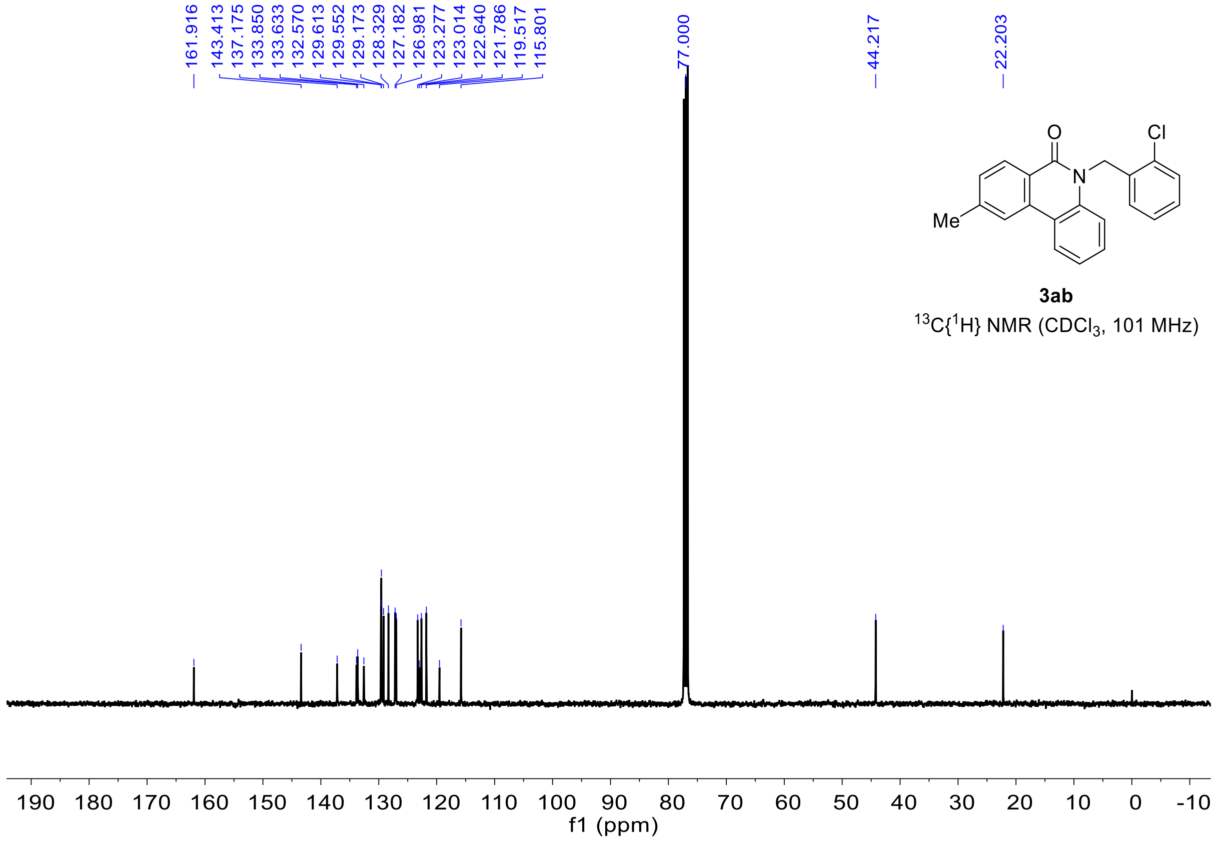

3ab

^{13}C{^{1}H} NMR ($CDCl_3$, 101 MHz)

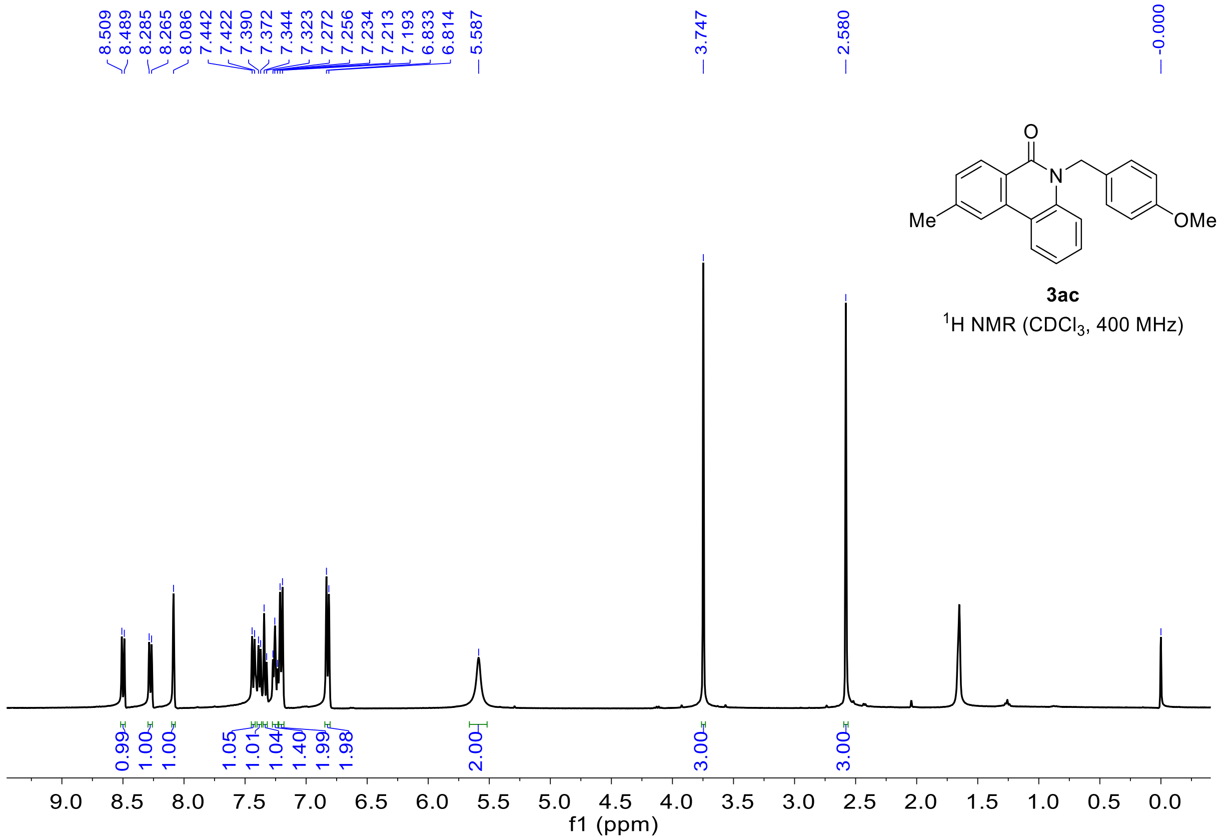
8.509 8.489 8.285 8.265 8.086 7.442 7.422 7.390 7.372 7.344 7.323 7.272 7.256 7.234 7.213 7.193 6.833 6.814 5.587
3.747
2.580
-0.000
O
N
Me
OMe
3ac
^{1}H NMR (CDCl$_3$, 400 MHz)
0.99 1.00 1.00 1.05 1.01 1.04 1.40 1.99 1.98 2.00 3.00 3.00
9.0 8.5 8.0 7.5 7.0 6.5 6.0 5.5 5.0 4.5 4.0 3.5 3.0 2.5 2.0 1.5 1.0 0.5 0.0
f1 (ppm)

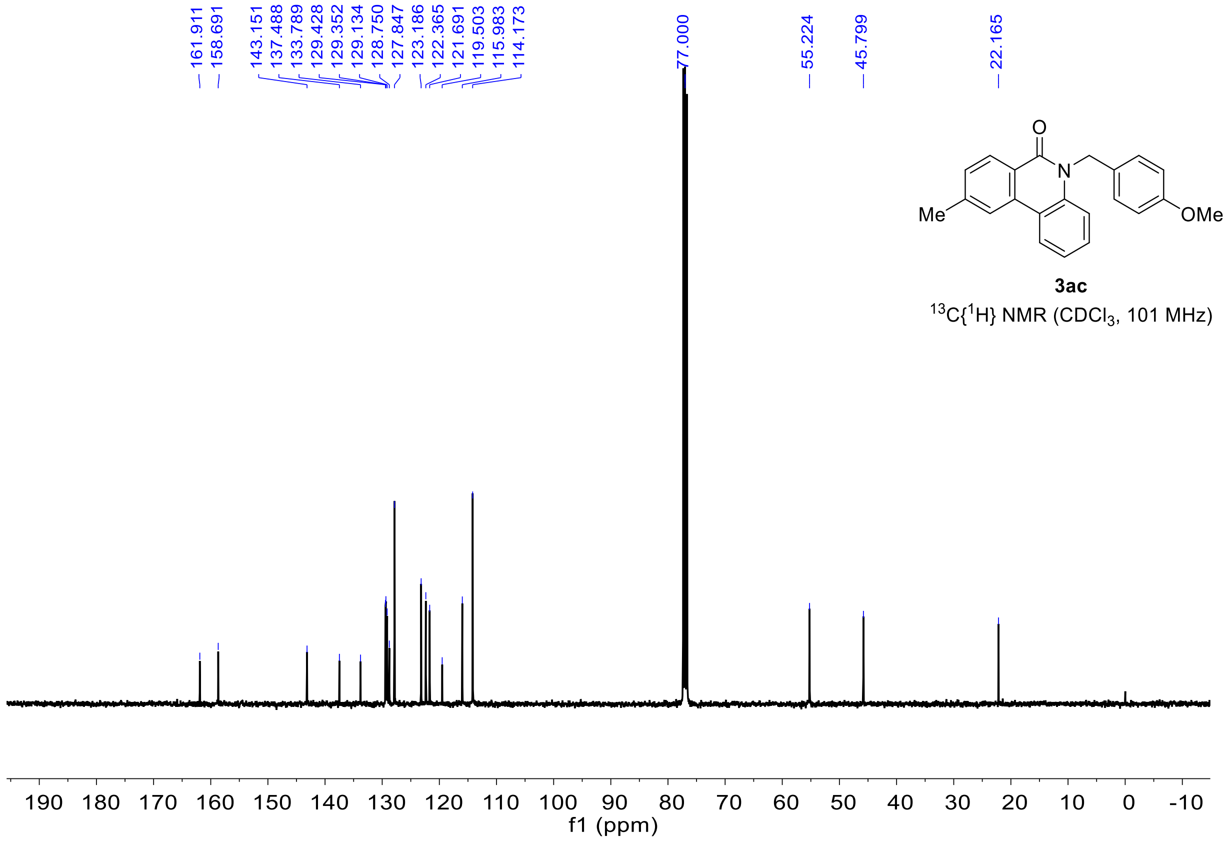
161.911 158.691
143.151 137.488 133.789 129.428 129.352 129.134 128.750 127.847 123.186 122.365 121.691 119.503 115.983 114.173
77.000
55.224
45.799
22.165
O
N
Me
OMe
3ac
^{13}C{^{1}H} NMR (CDCl$_3$, 101 MHz)
190 180 170 160 150 140 130 120 110 100 90 80 70 60 50 40 30 20 10 0 -10
f1 (ppm)

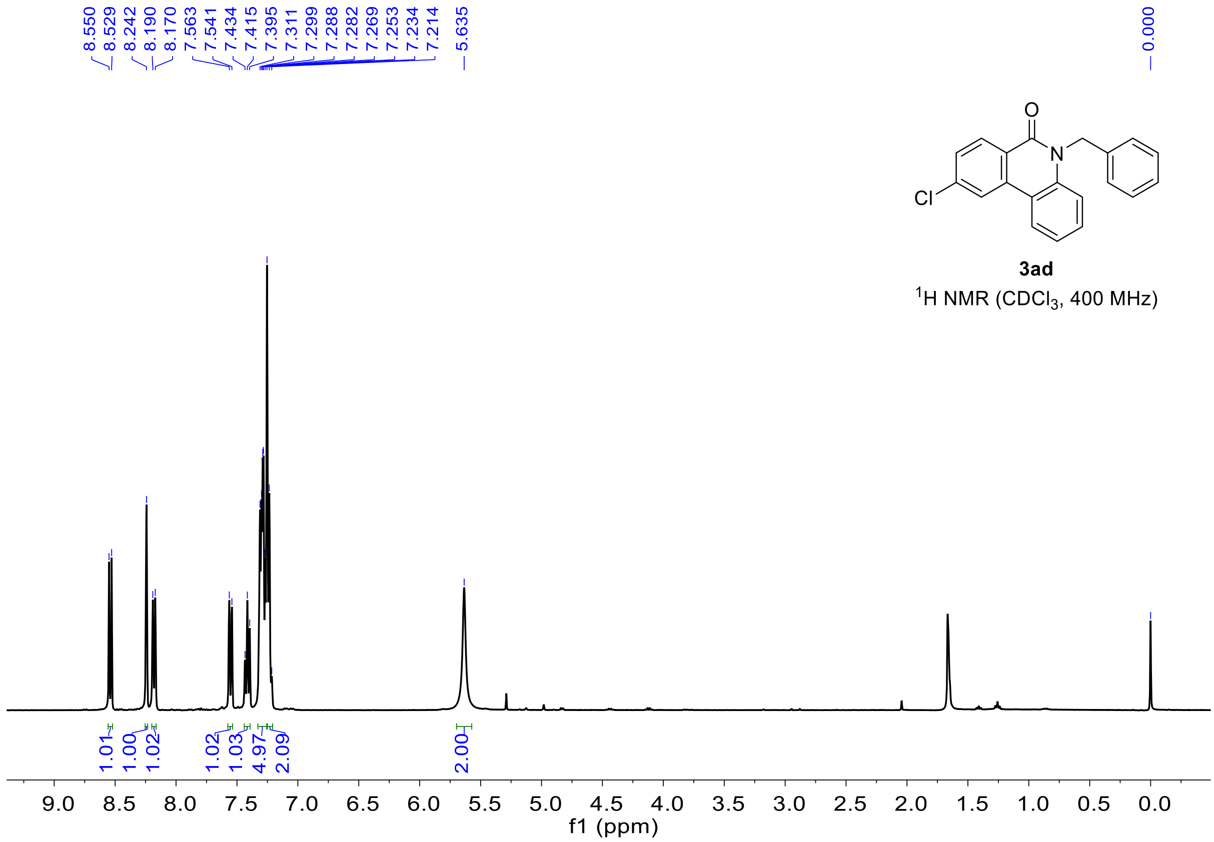
8.550
8.529
8.242
8.190
8.170
7.563
7.541
7.434
7.415
7.395
7.311
7.299
7.288
7.282
7.269
7.253
7.234
7.214
5.635
0.000
Cl
O
N
3ad
1H NMR (CDCl3, 400 MHz)
1.01
1.00
1.02
1.02
1.03
4.97
2.09
2.00
9.0 8.5 8.0 7.5 7.0 6.5 6.0 5.5 5.0 4.5 4.0 3.5 3.0 2.5 2.0 1.5 1.0 0.5 0.0
f1 (ppm)

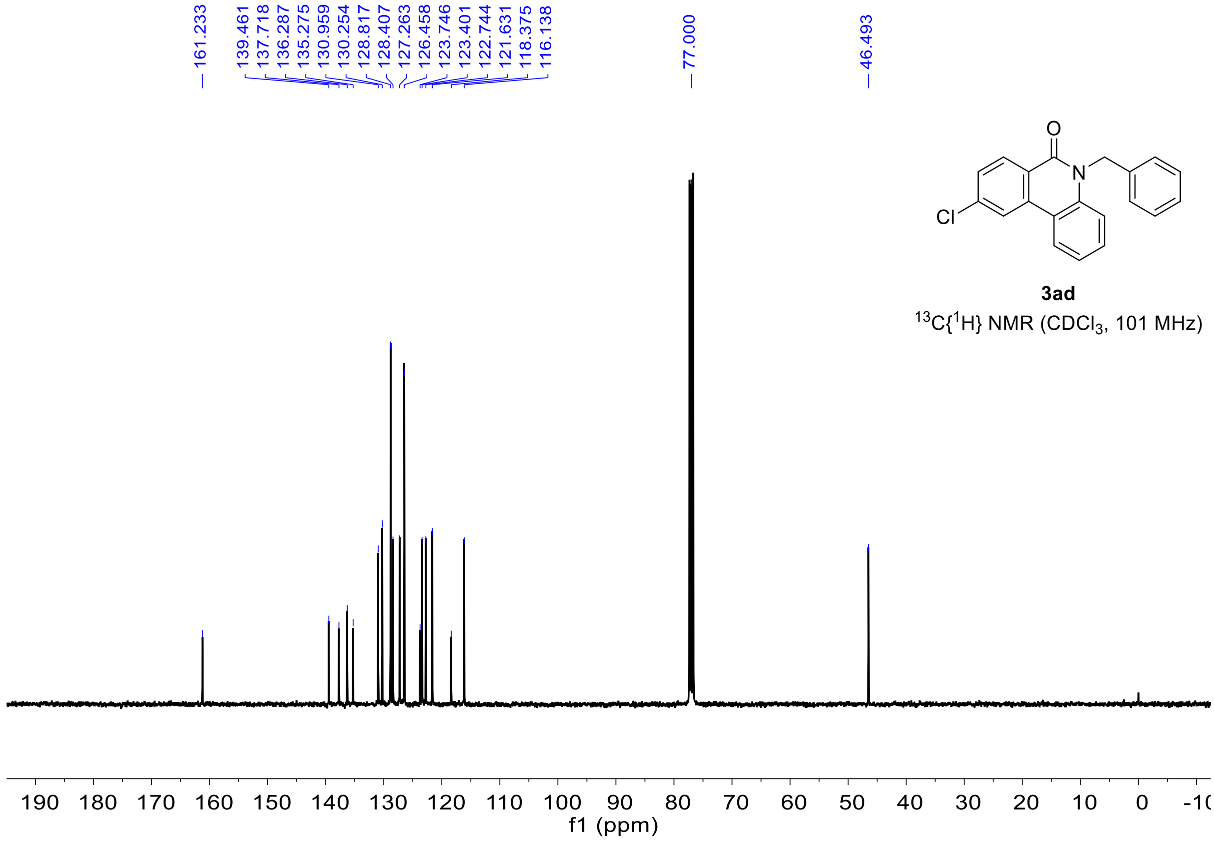
161.233
139.461
137.718
136.287
135.275
130.959
130.254
128.817
128.407
127.263
126.458
123.746
123.401
122.744
121.631
118.375
116.138
77.000
46.493
Cl
O
N
3ad
13C{1H} NMR (CDCl3, 101 MHz)
190 180 170 160 150 140 130 120 110 100 90 80 70 60 50 40 30 20 10 0
f1 (ppm)

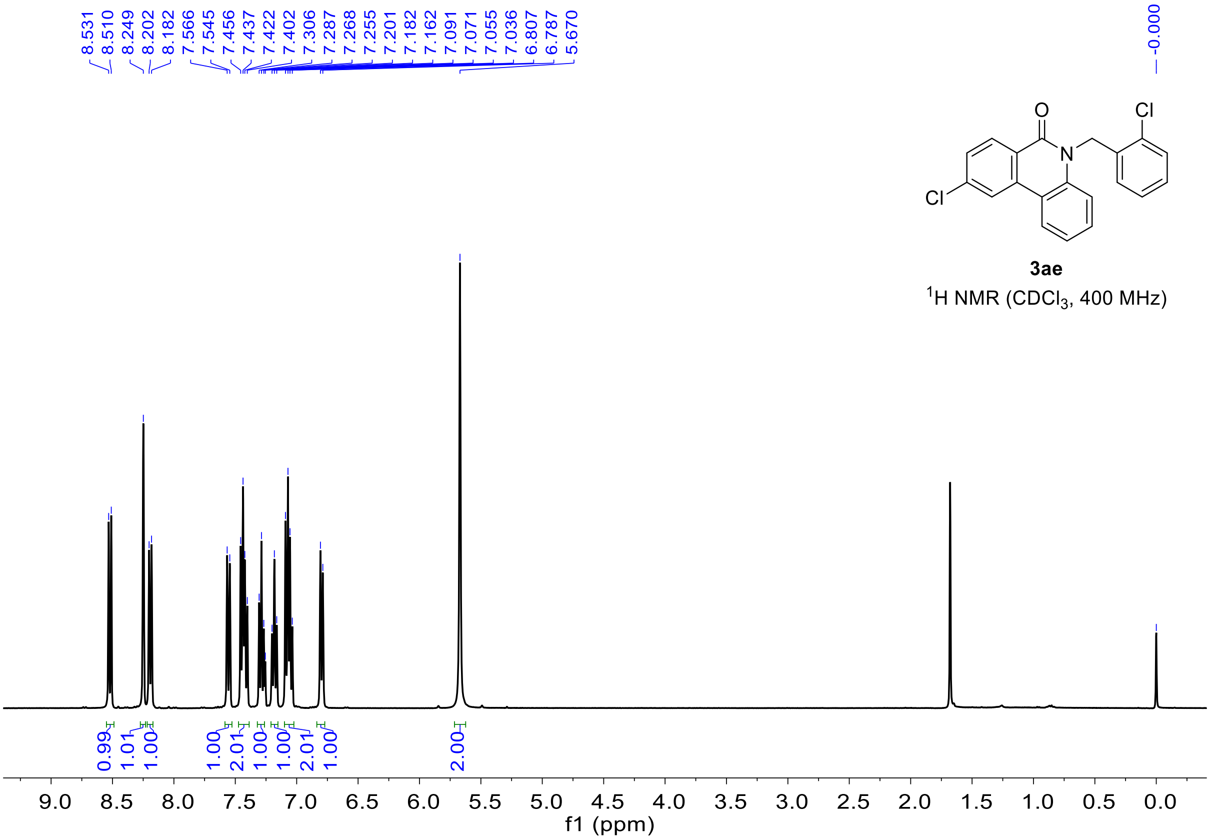

3ae
^{1}H NMR ($CDCl_3$, 400 MHz)

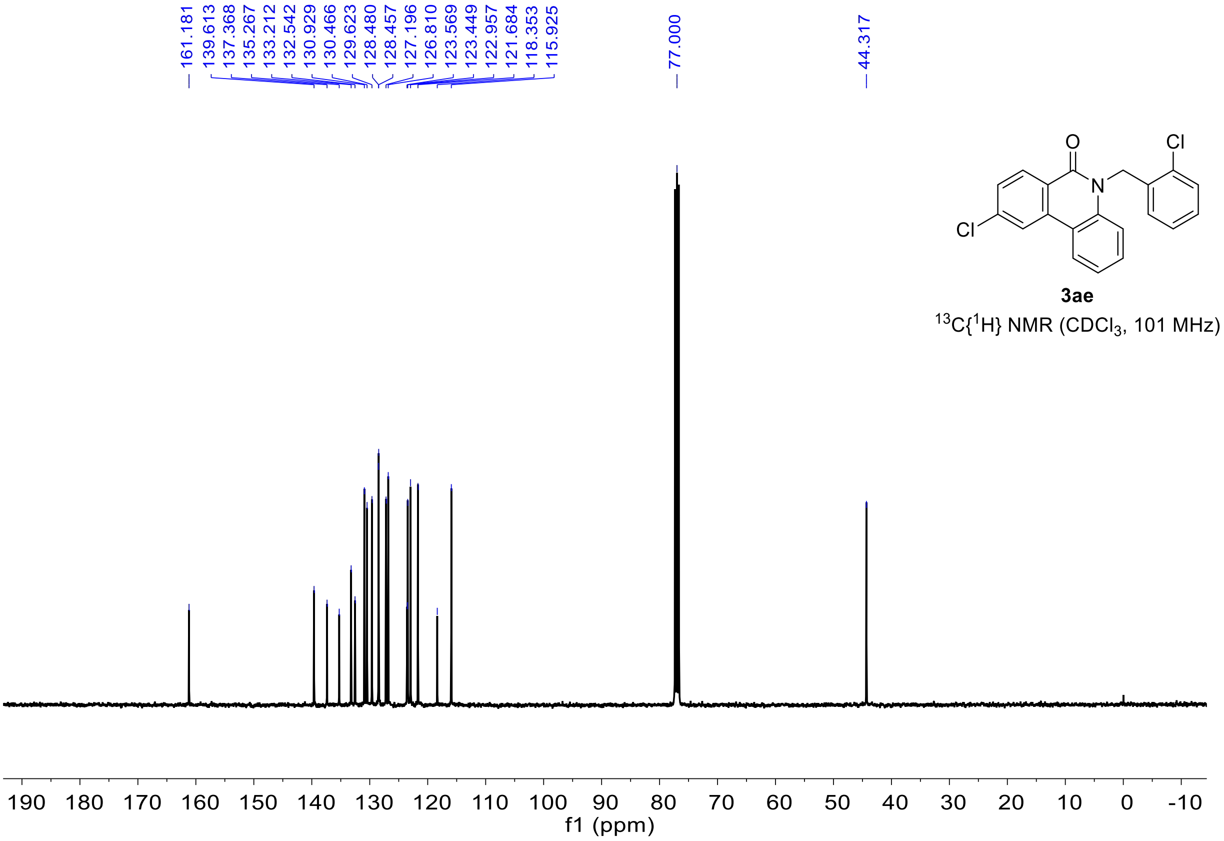

3ae
$^{13}C\{^1H\}$ NMR ($CDCl_3$, 101 MHz)

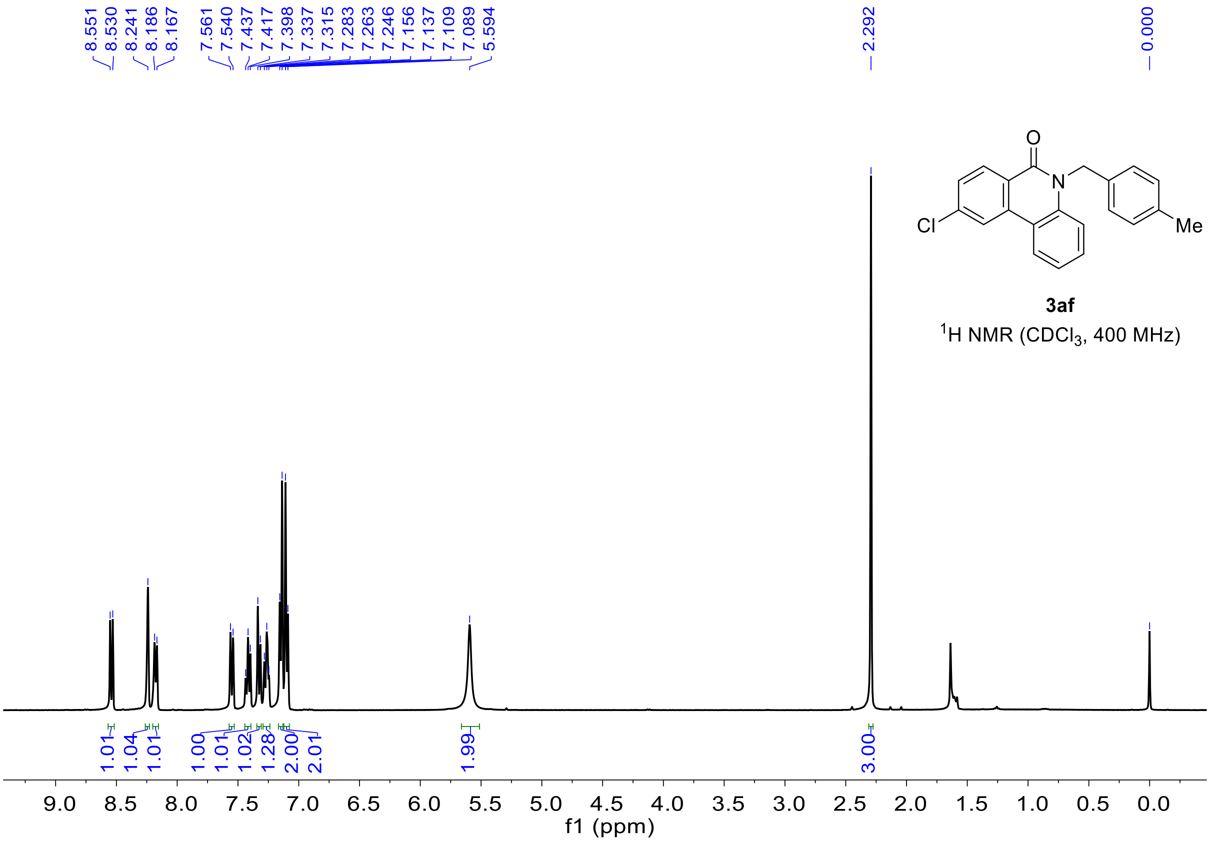

3af

^{1}H NMR ($CDCl_3$, 400 MHz)

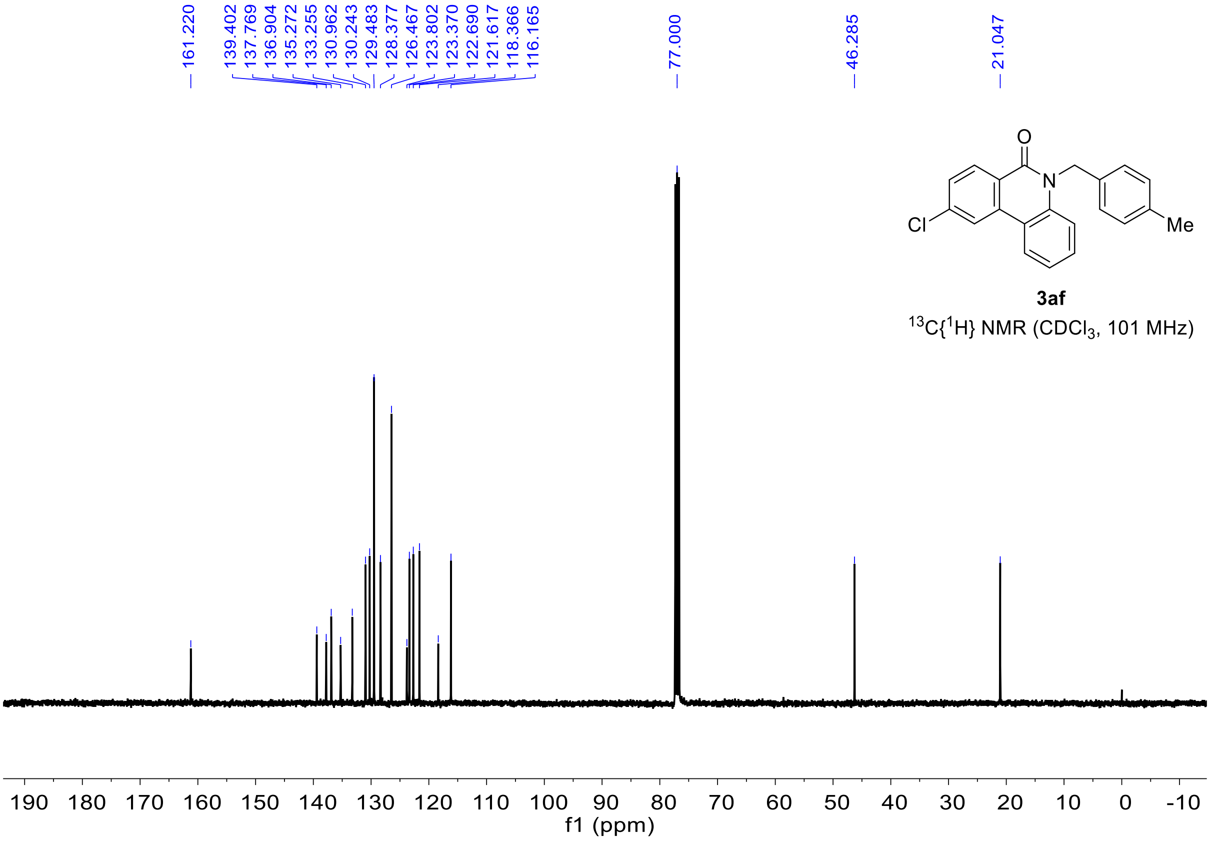

3af

$^{13}C\{^{1}H\}$ NMR ($CDCl_3$, 101 MHz)

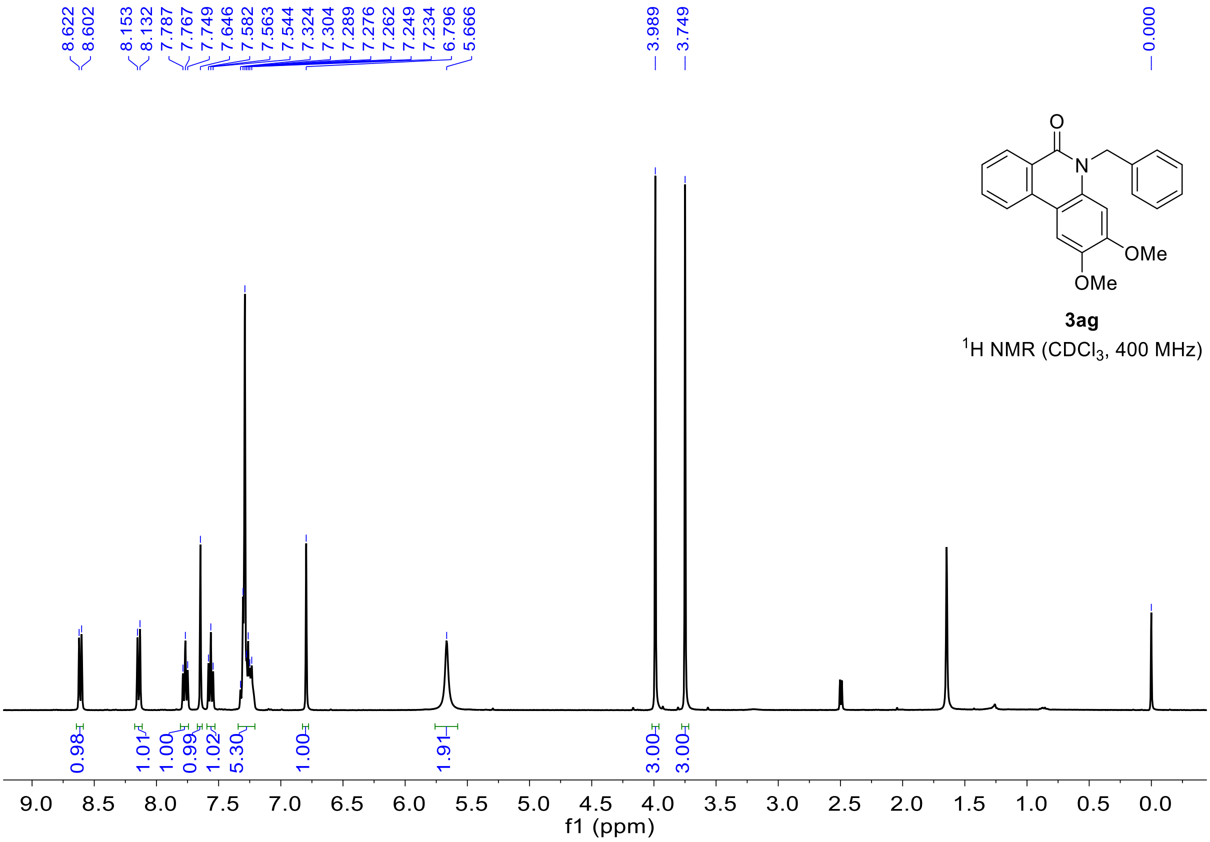
8.622
8.602
8.153
8.132
7.787
7.767
7.749
7.646
7.582
7.563
7.544
7.324
7.304
7.289
7.276
7.262
7.249
7.234
6.796
5.666
3.989
3.749
0.000
O
N
OMe
OMe
3ag
^{1}H NMR ($CDCl_3$, 400 MHz)
0.98
1.01
1.00
0.99
1.02
5.30
1.00
1.91
3.00
3.00
9.0 8.5 8.0 7.5 7.0 6.5 6.0 5.5 5.0 4.5 4.0 3.5 3.0 2.5 2.0 1.5 1.0 0.5 0.0
f1 (ppm)

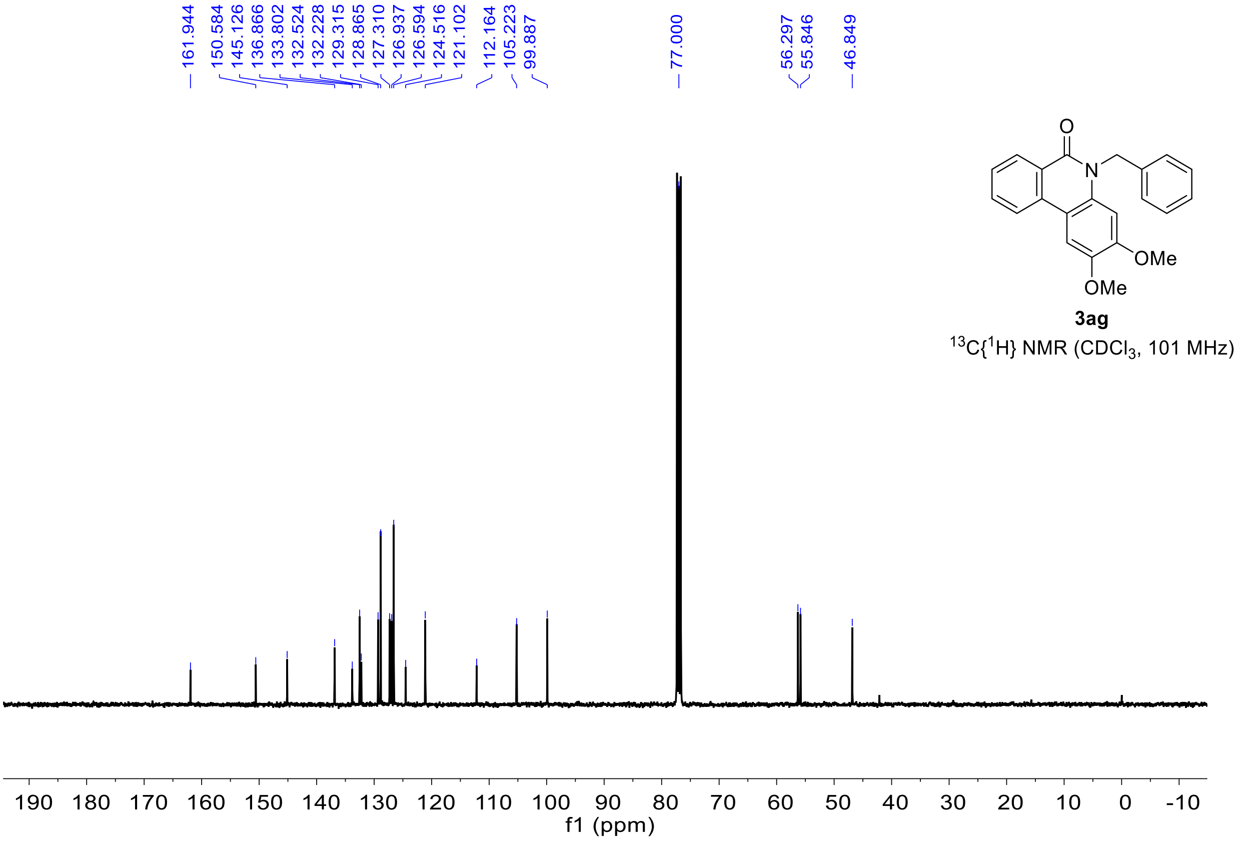
161.944
150.584
145.126
136.866
133.802
132.524
132.228
129.315
128.865
127.310
126.937
126.594
124.516
121.102
112.164
105.223
99.887
77.000
56.297
55.846
46.849
O
N
OMe
OMe
3ag
$^{13}C\{^1H\}$ NMR ($CDCl_3$, 101 MHz)
190 180 170 160 150 140 130 120 110 100 90 80 70 60 50 40 30 20 10 0 -10
f1 (ppm)

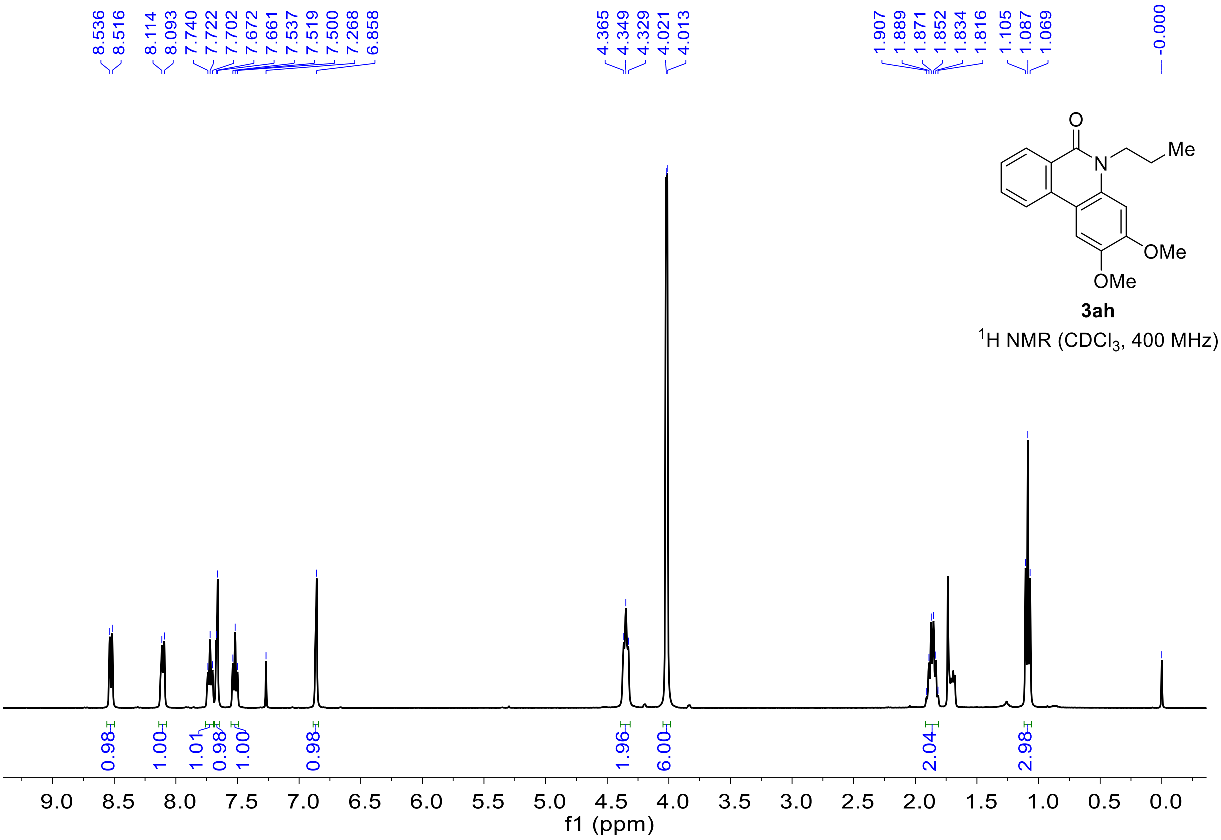
8.536
8.516
8.114
8.093
7.740
7.722
7.702
7.672
7.661
7.537
7.519
7.500
7.268
6.858
4.365
4.349
4.329
4.021
4.013
1.907
1.889
1.871
1.852
1.834
1.816
1.105
1.087
1.069
-0.000
O
N
Me
OMe
OMe
3ah
^{1}H NMR (CDCl$_3$, 400 MHz)
0.98
1.00
1.01
0.98
1.00
0.98
1.96
6.00
2.04
2.98
9.0 8.5 8.0 7.5 7.0 6.5 6.0 5.5 5.0 4.5 4.0 3.5 3.0 2.5 2.0 1.5 1.0 0.5 0.0
f1 (ppm)

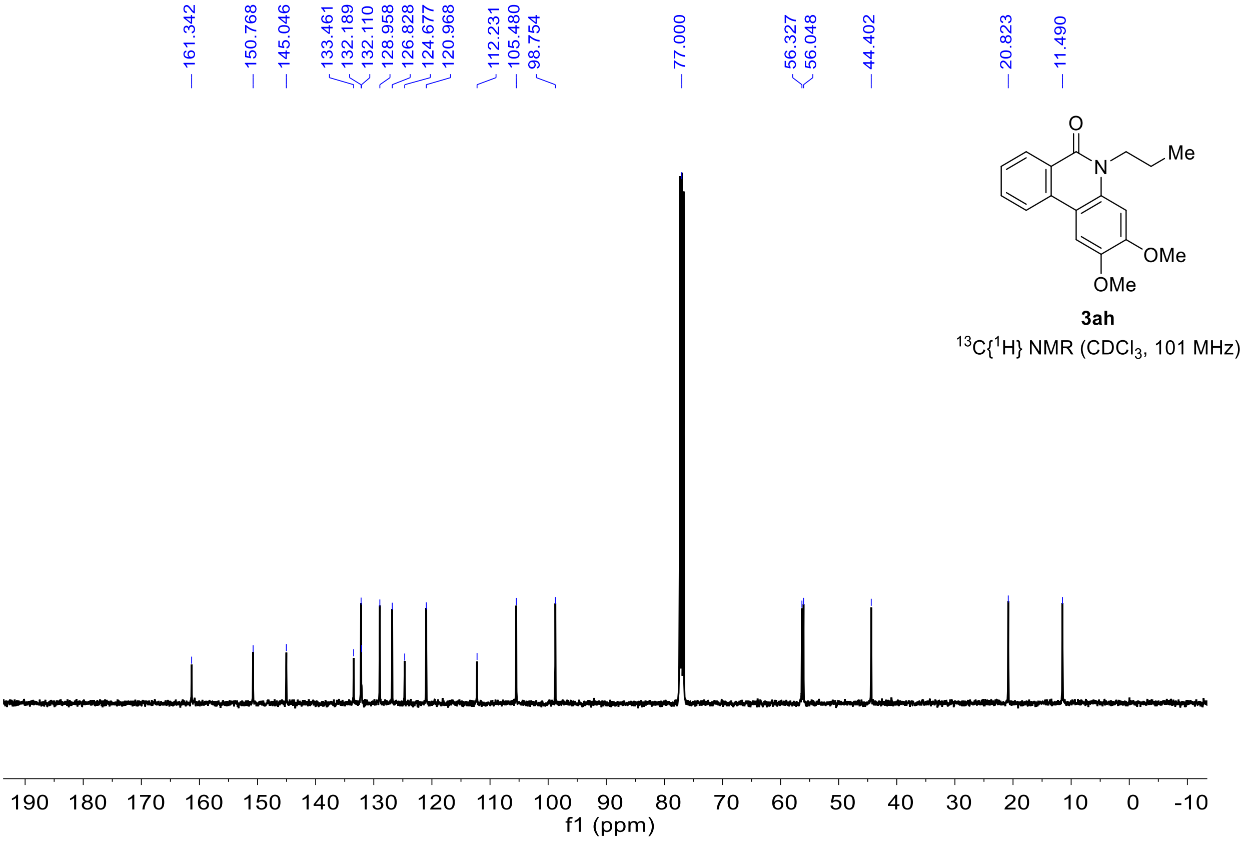
161.342
150.768
145.046
133.461
132.189
132.110
128.958
126.828
124.677
120.968
112.231
105.480
98.754
77.000
56.327
56.048
44.402
20.823
11.490
O
N
Me
OMe
OMe
3ah
^{13}C{^{1}H} NMR (CDCl$_3$, 101 MHz)
190 180 170 160 150 140 130 120 110 100 90 80 70 60 50 40 30 20 10 0 -10
f1 (ppm)

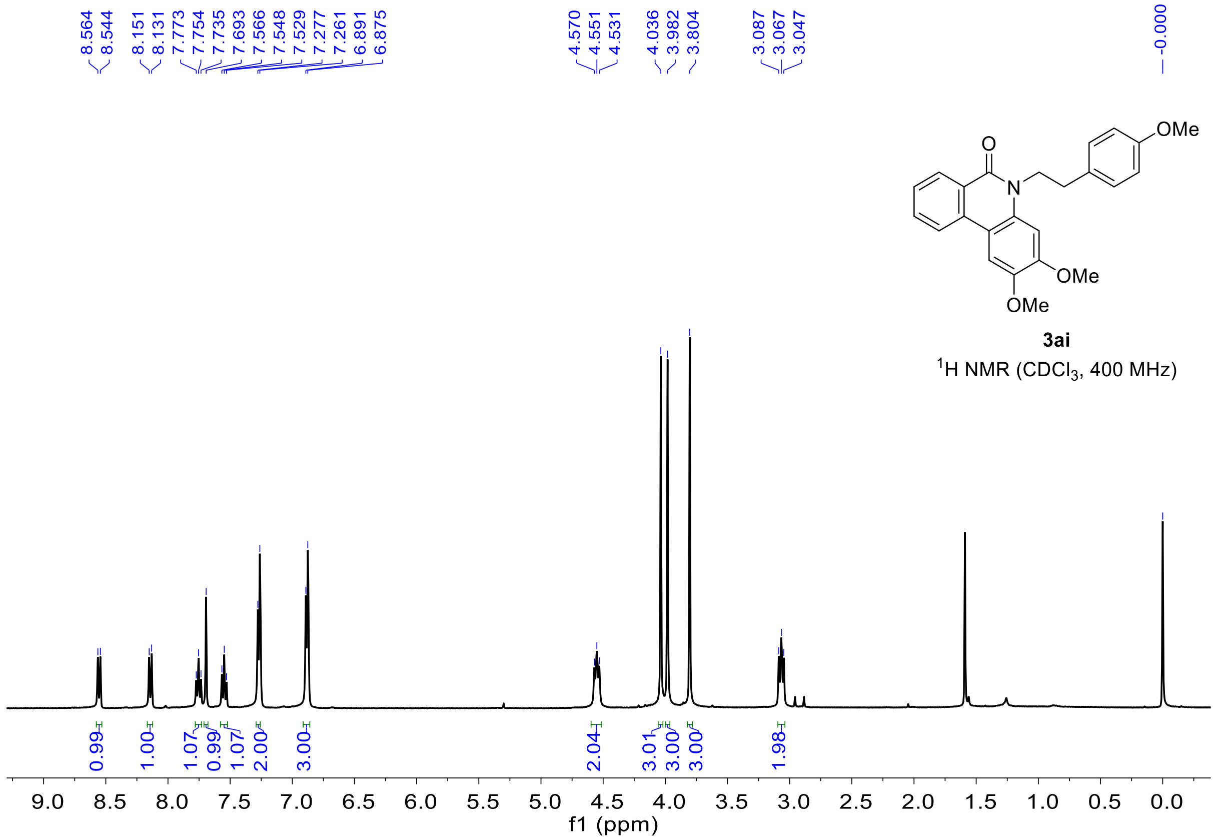
8.564
8.544
8.151
8.131
7.773
7.754
7.735
7.693
7.566
7.548
7.529
7.277
7.261
6.891
6.875
4.570
4.551
4.531
4.036
3.982
3.804
3.087
3.067
3.047
-0.000
OMe
O
N
OMe
OMe
3ai
^{1}H NMR (CDCl$_3$, 400 MHz)
0.99
1.00
1.07
0.99
1.07
2.00
3.00
2.04
3.01
3.00
3.00
1.98
f1 (ppm)

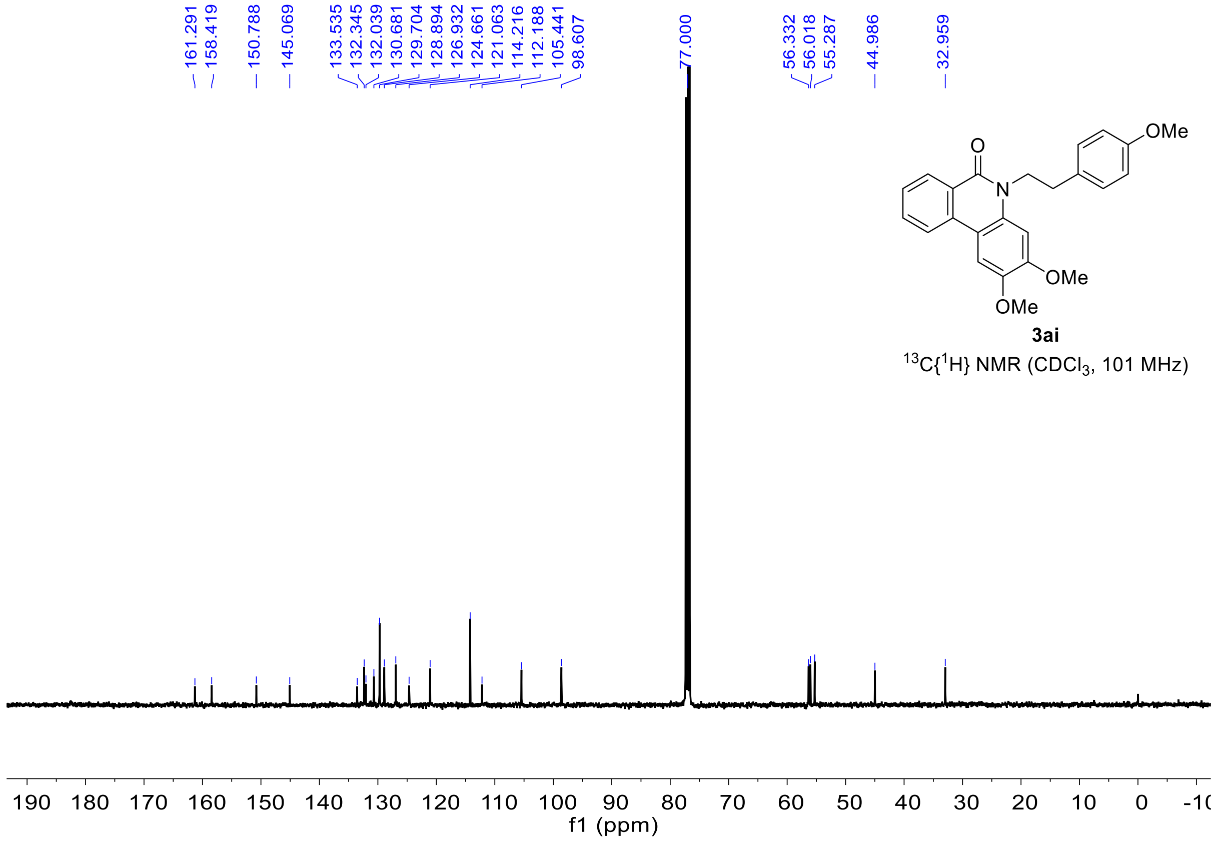
161.291
158.419
150.788
145.069
133.535
132.345
132.039
130.681
129.704
128.894
126.932
124.661
121.063
114.216
112.188
105.441
98.607
77.000
56.332
56.018
55.287
44.986
32.959
OMe
O
N
OMe
OMe
3ai
^{13}C{^{1}H} NMR (CDCl$_3$, 101 MHz)
f1 (ppm)

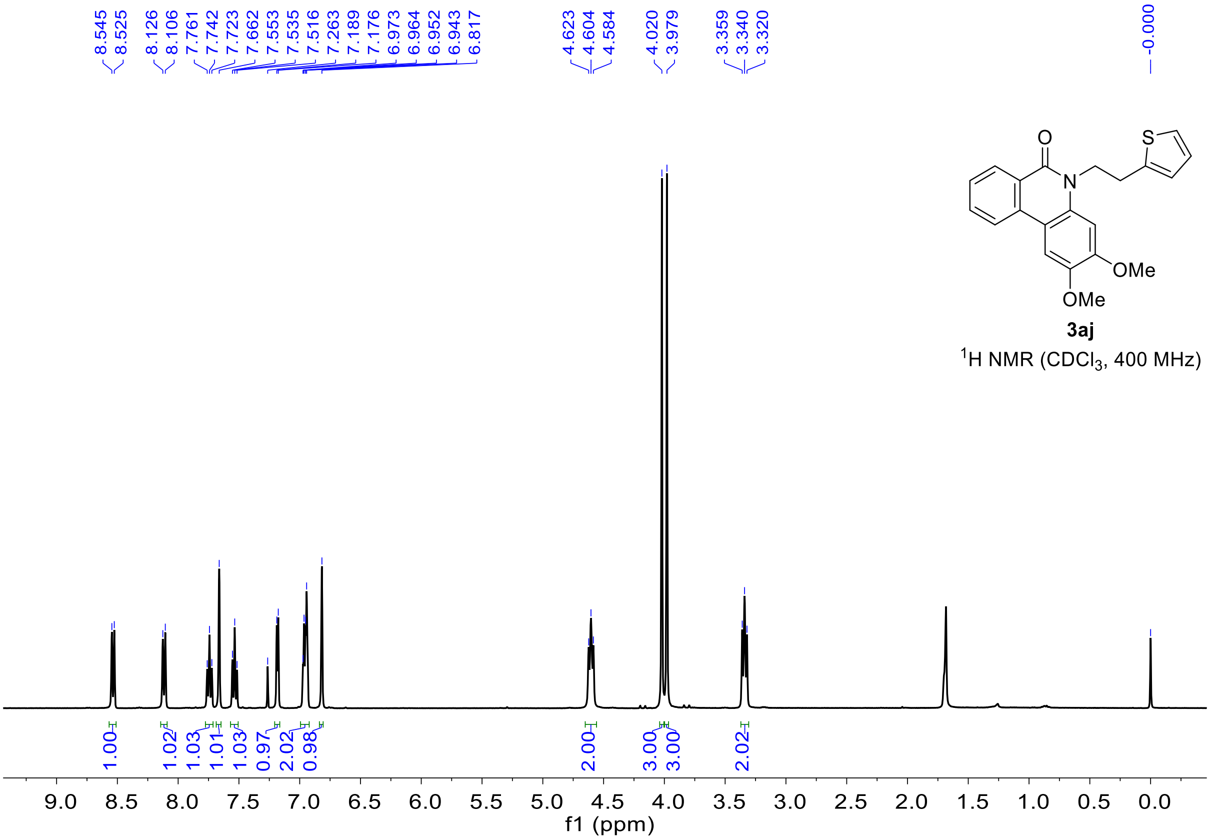
8.545 8.525 8.126 8.106 7.761 7.742 7.723 7.662 7.553 7.535 7.516 7.263 7.189 7.176 6.973 6.964 6.952 6.943 6.817
4.623 4.604 4.584
4.020 3.979
3.359 3.340 3.320
-0.000
O
S
N
OMe
OMe
3aj
^{1}H NMR ($CDCl_3$, 400 MHz)
1.00 1.02 1.03 1.01 1.03 0.97 2.02 0.98 2.00 3.00 3.00 2.02
9.0 8.5 8.0 7.5 7.0 6.5 6.0 5.5 5.0 4.5 4.0 3.5 3.0 2.5 2.0 1.5 1.0 0.5 0.0
f1 (ppm)

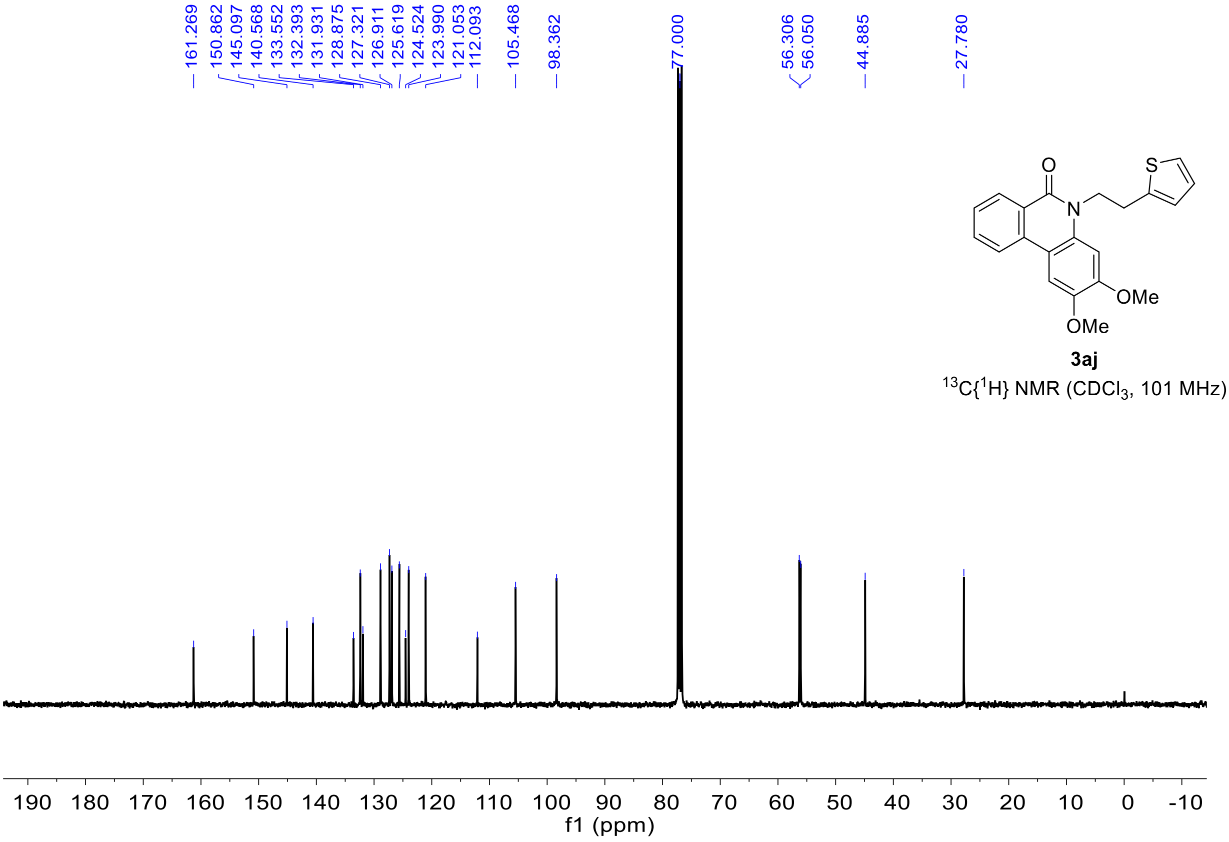
161.269
150.862 145.097 140.568 133.552 132.393 131.931 128.875 127.321 126.911 125.619 124.524 123.990 121.053 112.093
105.468
98.362
77.000
56.306 56.050
44.885
27.780
O
S
N
OMe
OMe
3aj
^{13}C{^{1}H} NMR ($CDCl_3$, 101 MHz)
190 180 170 160 150 140 130 120 110 100 90 80 70 60 50 40 30 20 10 0 -10
f1 (ppm)

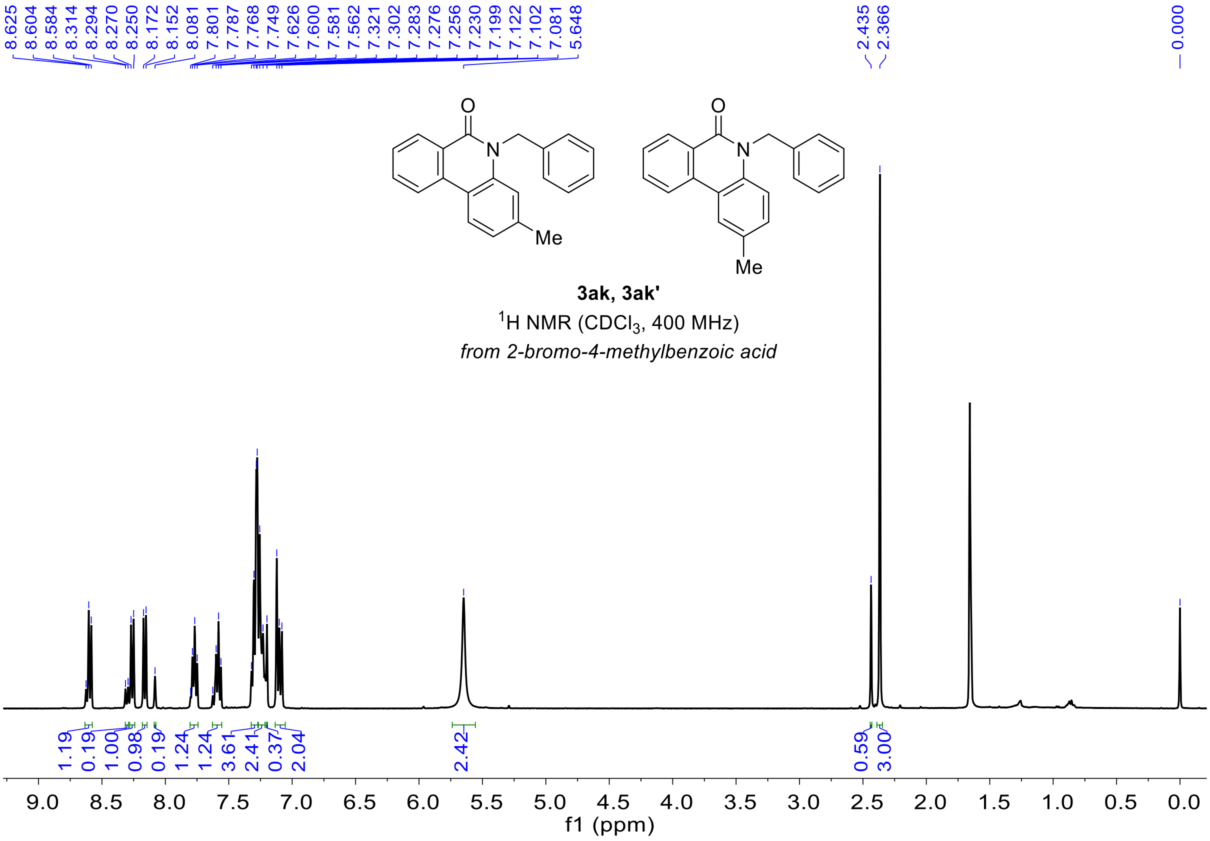
8.625
8.604
8.584
8.314
8.294
8.270
8.250
8.172
8.152
8.081
7.801
7.787
7.768
7.749
7.626
7.600
7.581
7.562
7.321
7.302
7.283
7.276
7.256
7.230
7.199
7.122
7.102
7.081
5.648
2.435
2.366
0.000
O
N
Me
3ak, 3ak'
1H NMR (CDCl3, 400 MHz)
from 2-bromo-4-methylbenzoic acid
1.19
0.19
1.00
0.98
0.19
1.24
1.24
3.61
2.41
0.37
2.04
2.42
0.59
3.00
9.0 8.5 8.0 7.5 7.0 6.5 6.0 5.5 5.0 4.5 4.0 3.5 3.0 2.5 2.0 1.5 1.0 0.5 0.0
f1 (ppm)

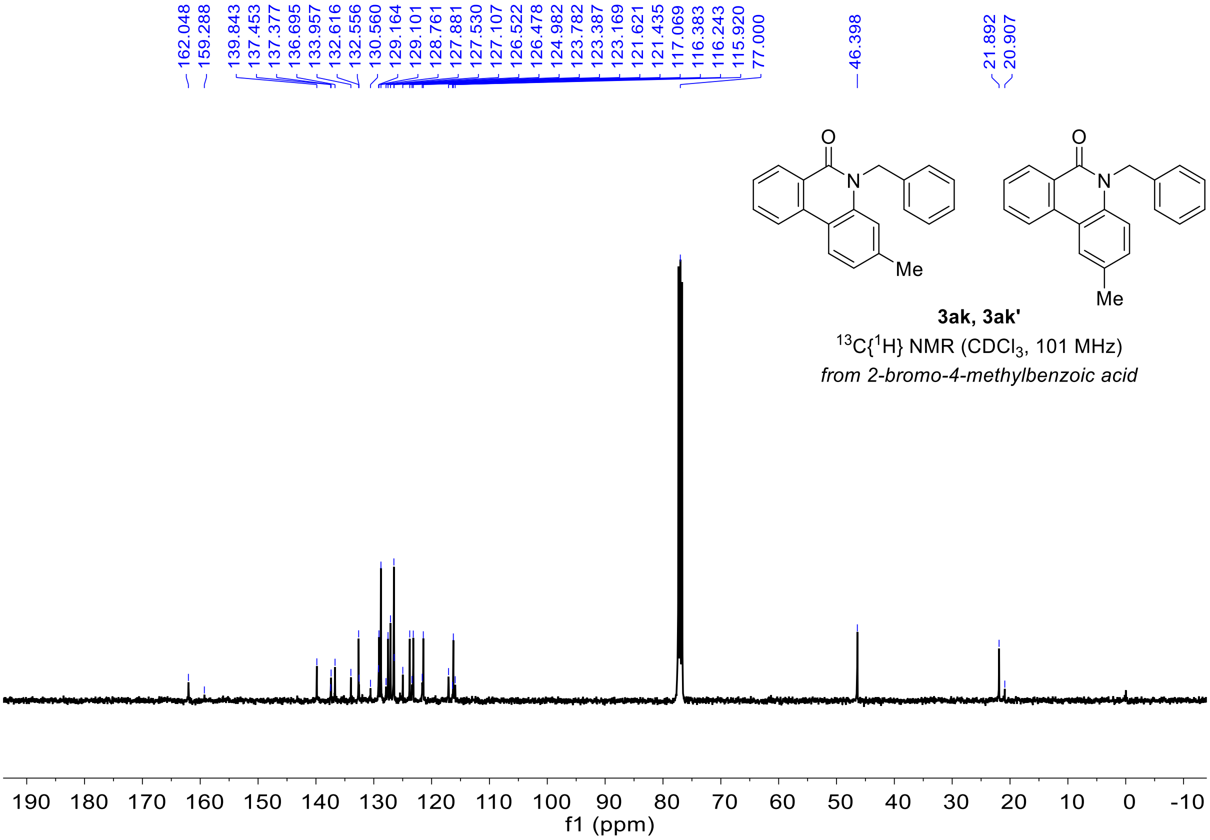
162.048
159.288
139.843
137.453
137.377
136.695
133.957
132.616
132.556
130.560
129.164
129.101
128.761
127.881
127.530
127.107
126.522
126.478
124.982
123.782
123.387
123.169
121.621
121.435
117.069
116.383
116.243
115.920
77.000
46.398
21.892
20.907
O
N
Me
3ak, 3ak'
13C{1H} NMR (CDCl3, 101 MHz)
from 2-bromo-4-methylbenzoic acid
190 180 170 160 150 140 130 120 110 100 90 80 70 60 50 40 30 20 10 0 -10
f1 (ppm)

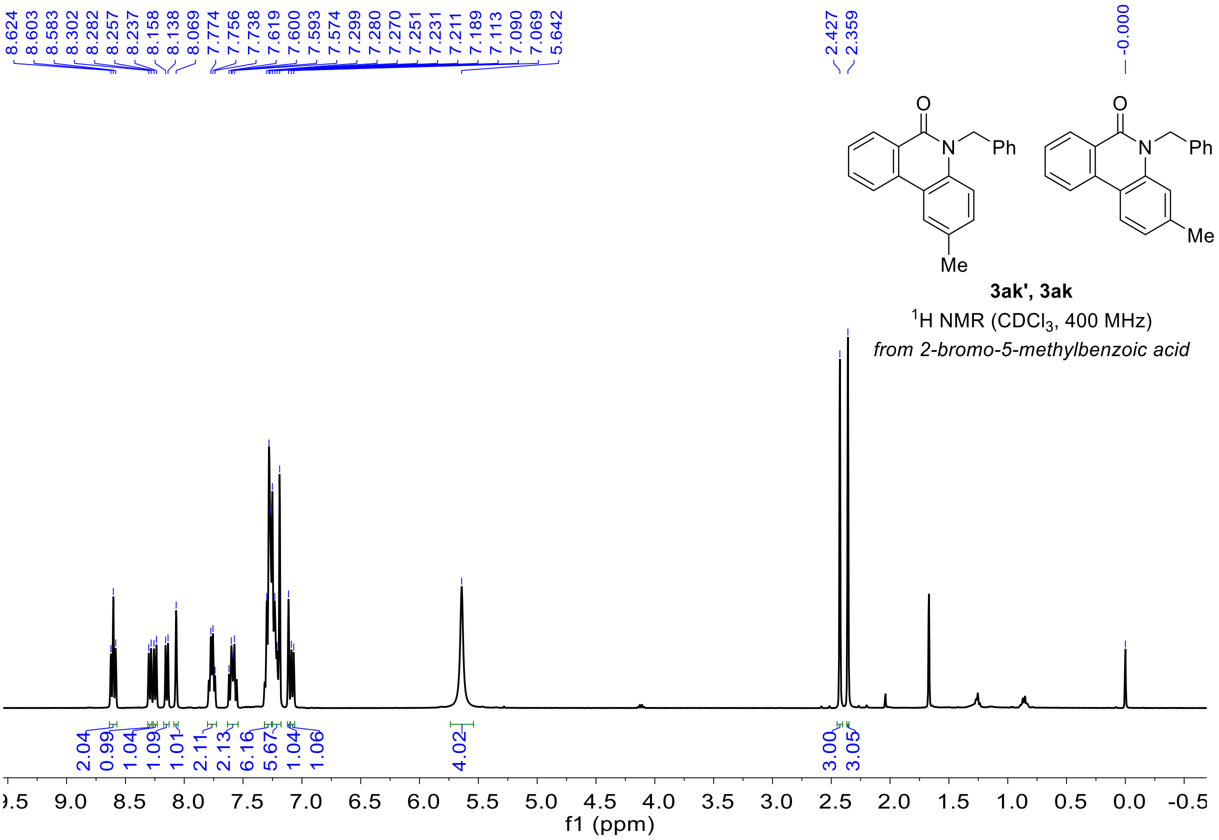

3ak', 3ak
^{1}H NMR ($CDCl_3$, 400 MHz)
from 2-bromo-5-methylbenzoic acid

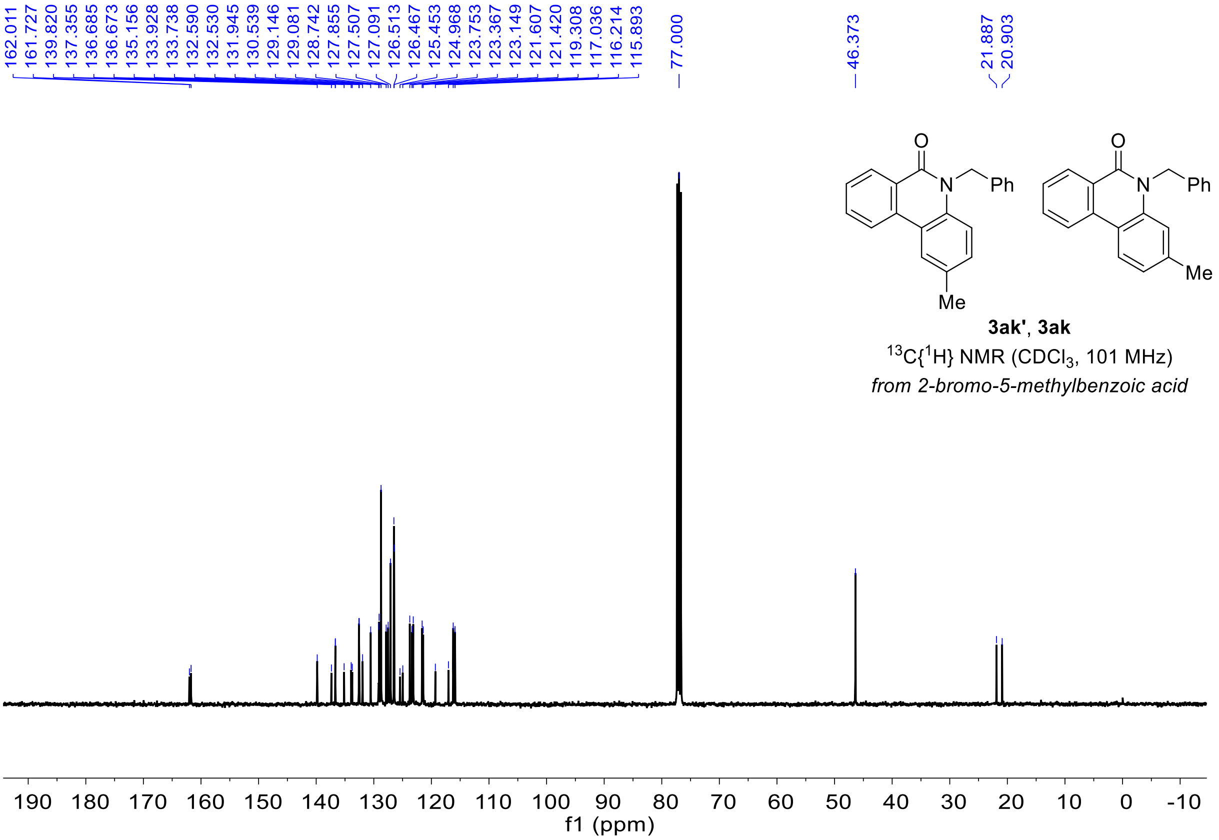

3ak', 3ak
^{13}C{^{1}H} NMR ($CDCl_3$, 101 MHz)
from 2-bromo-5-methylbenzoic acid

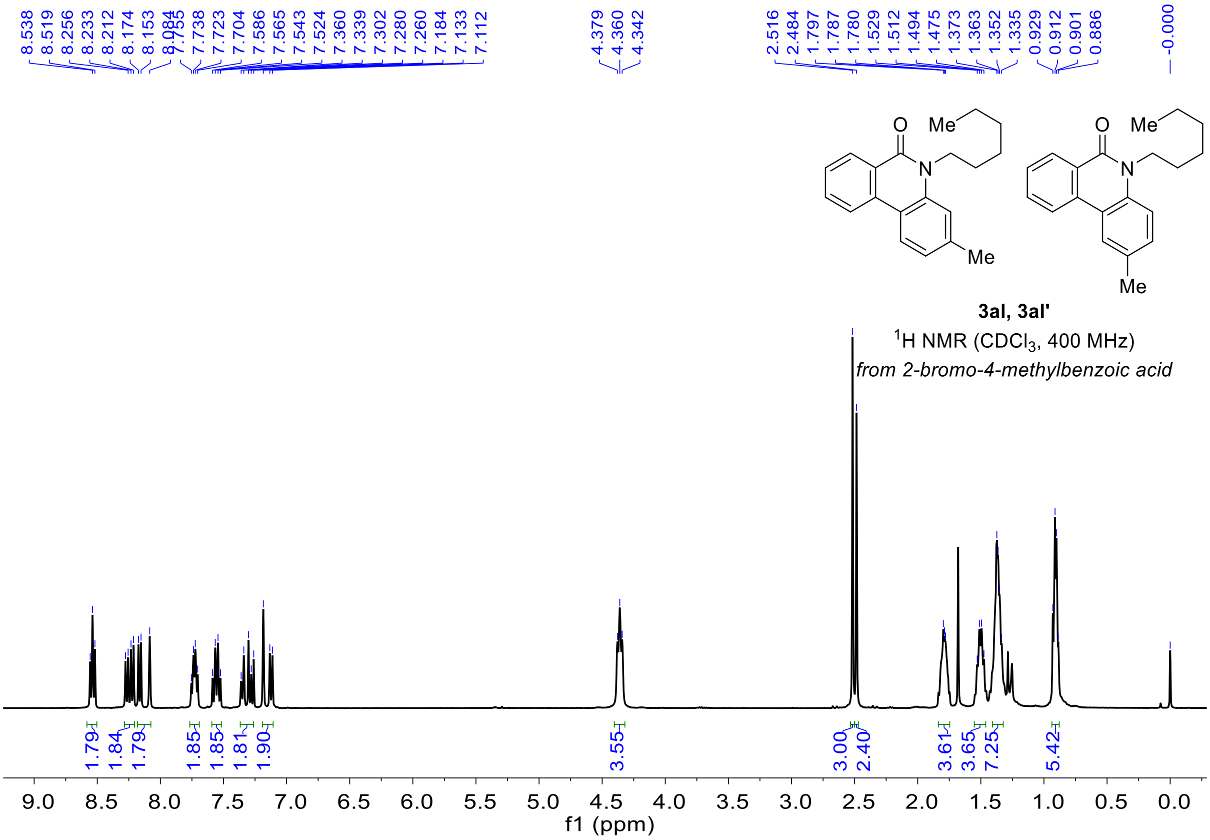
8.538 8.519 8.256 8.233 8.212 8.174 8.153 8.084 7.755 7.738 7.723 7.704 7.586 7.565 7.543 7.524 7.360 7.339 7.302 7.280 7.260 7.184 7.133 7.112
4.379 4.360 4.342
2.516 2.484 1.797 1.787 1.780 1.529 1.512 1.494 1.475 1.373 1.363 1.352 1.335 0.929 0.912 0.901 0.886
-0.000
O
Me
N
Me
3al, 3al'
^{1}H NMR (CDCl$_3$, 400 MHz)
from 2-bromo-4-methylbenzoic acid
1.79 1.84 1.79 1.85 1.85 1.81 1.90 3.55 3.00 2.40 3.61 3.65 7.25 5.42
9.0 8.5 8.0 7.5 7.0 6.5 6.0 5.5 5.0 4.5 4.0 3.5 3.0 2.5 2.0 1.5 1.0 0.5 0.0
f1 (ppm)

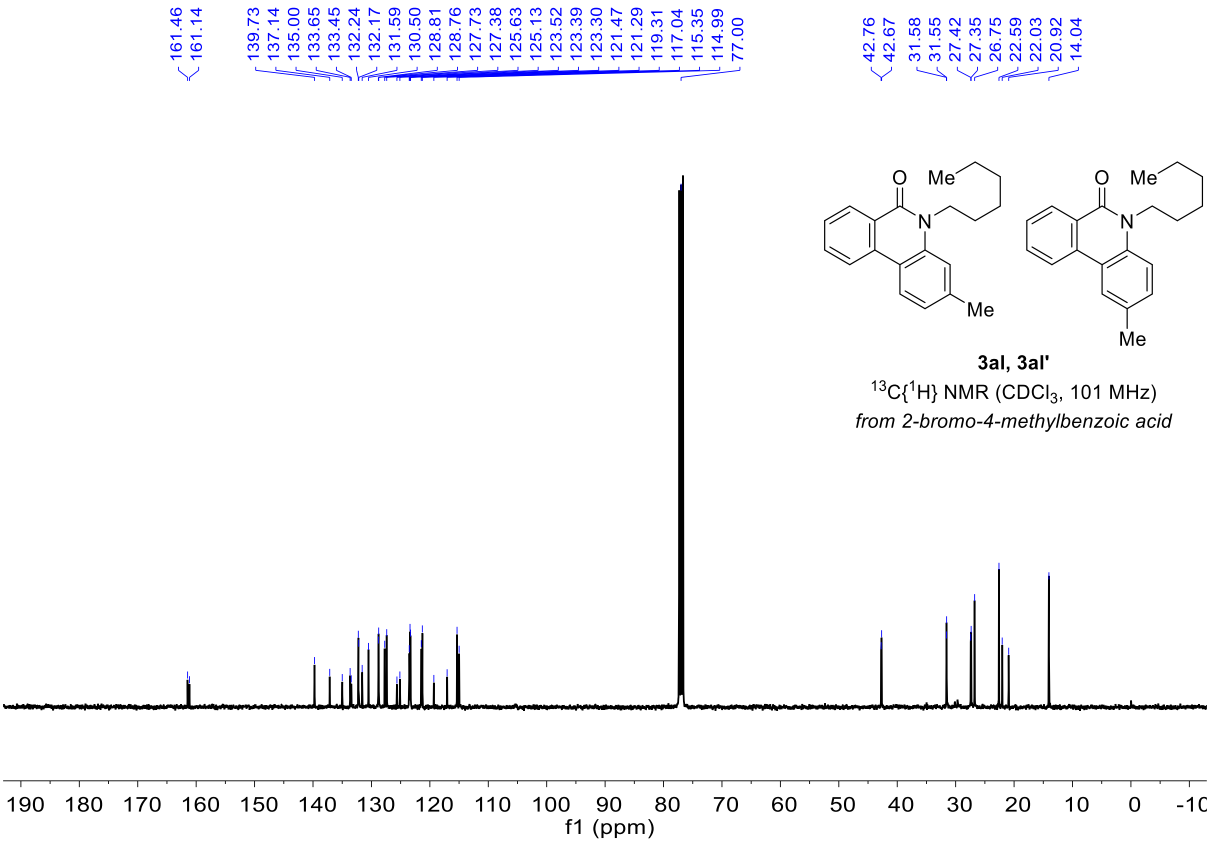
161.46 161.14
139.73 137.14 135.00 133.65 133.45 132.24 132.17 131.59 130.50 128.81 128.76 127.73 127.38 125.63 125.13 123.52 123.39 123.30 121.47 121.29 119.31 117.04 115.35 114.99 77.00
42.76 42.67 31.58 31.55 27.42 27.35 26.75 22.59 22.03 20.92 14.04
O
Me
N
Me
3al, 3al'
^{13}C{^{1}H} NMR (CDCl$_3$, 101 MHz)
from 2-bromo-4-methylbenzoic acid
190 180 170 160 150 140 130 120 110 100 90 80 70 60 50 40 30 20 10 0 -10
f1 (ppm)

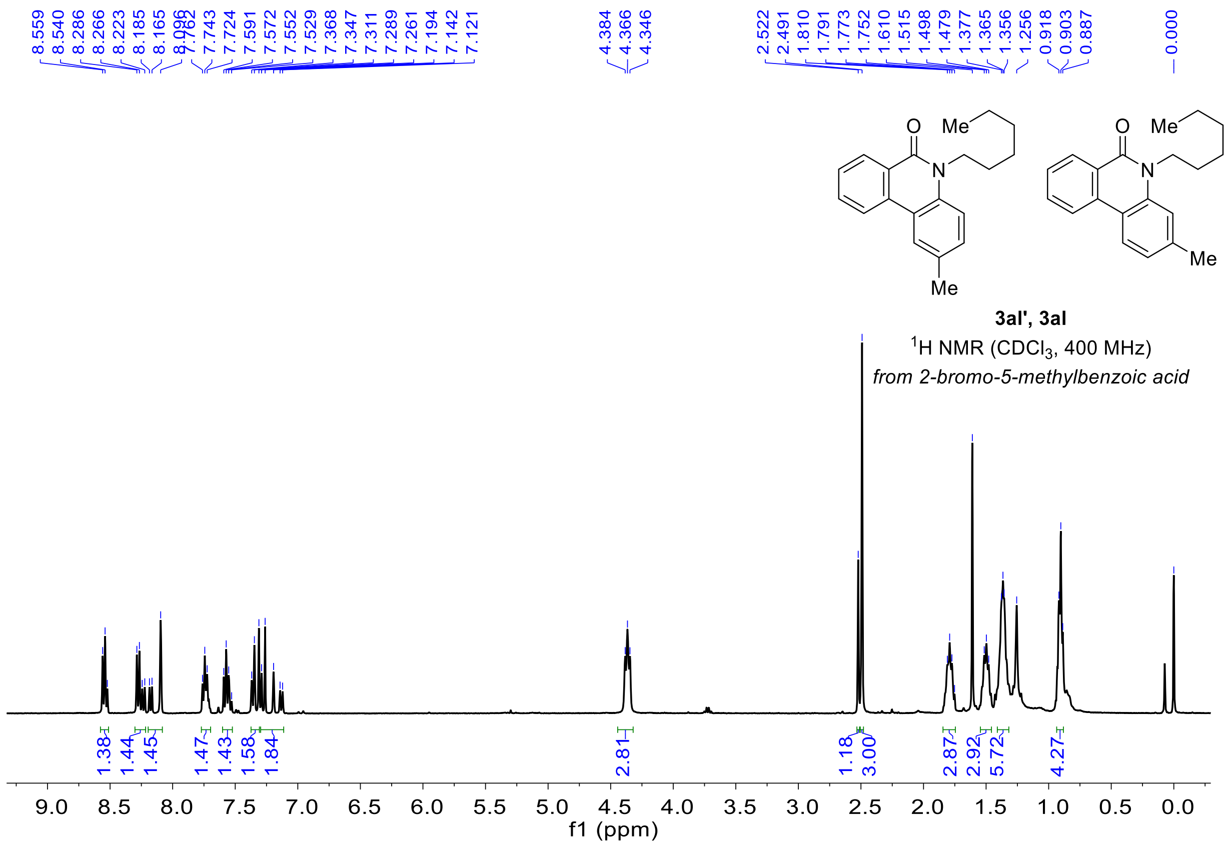
8.559 8.540 8.286 8.266 8.223 8.185 8.165 8.096 7.762 7.743 7.724 7.591 7.572 7.552 7.529 7.368 7.347 7.311 7.289 7.261 7.194 7.142 7.121
4.384 4.366 4.346
2.522 2.491 1.810 1.791 1.773 1.752 1.610 1.515 1.498 1.479 1.377 1.365 1.356 1.256 0.918 0.903 0.887
0.000
O
Me
N
Me
3al', 3al
^{1}H NMR ($CDCl_3$, 400 MHz)
from 2-bromo-5-methylbenzoic acid
1.38 1.44 1.45 1.47 1.43 1.58 1.84 2.81 1.18 3.00 2.87 2.92 5.72 4.27
9.0 8.5 8.0 7.5 7.0 6.5 6.0 5.5 5.0 4.5 4.0 3.5 3.0 2.5 2.0 1.5 1.0 0.5 0.0
f1 (ppm)

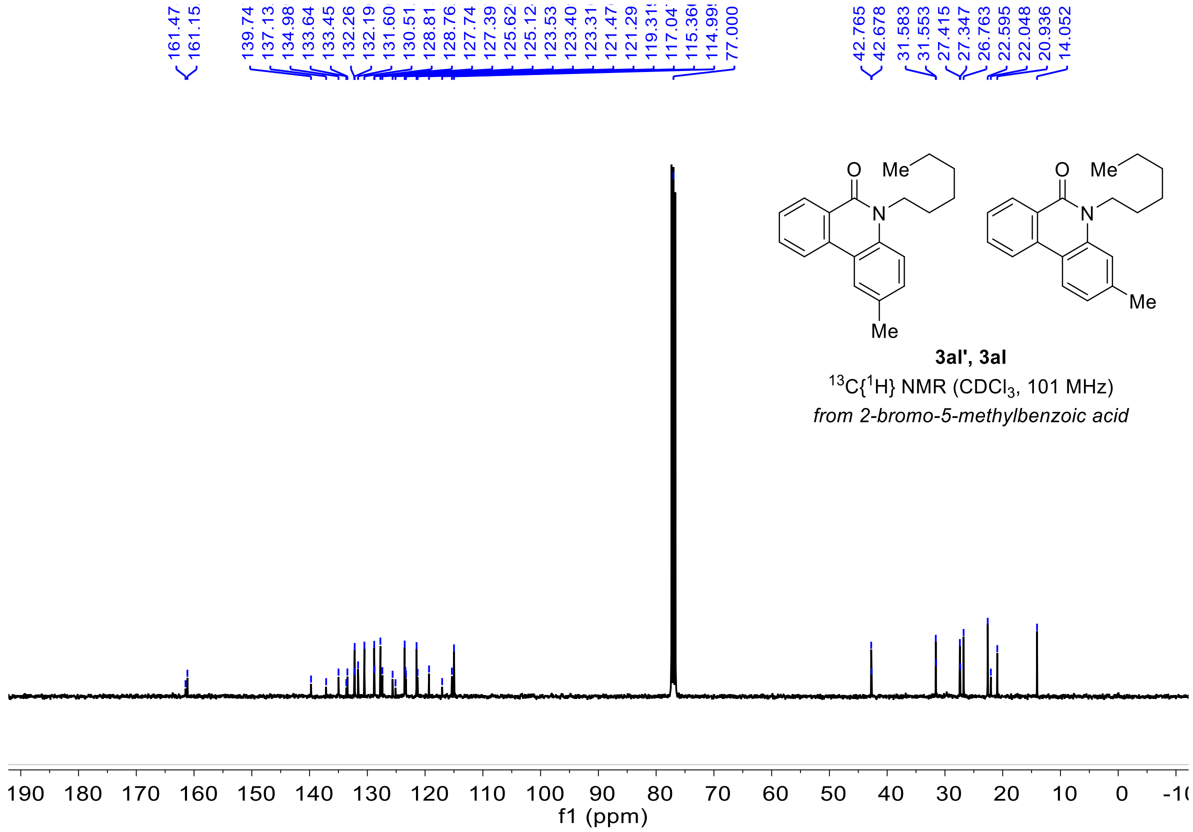
161.47 161.15
139.74 137.13 134.98 133.64 133.45 132.26 132.19 131.60 130.51 128.81 128.76 127.74 127.39 125.62 125.12 123.53 123.40 123.31 121.47 121.29 119.31 117.04 115.36 114.99 77.000
42.765 42.678 31.583 31.553 27.415 27.347 26.763 22.595 22.048 20.936 14.052
O
Me
N
Me
3al', 3al
^{13}C{^{1}H} NMR ($CDCl_3$, 101 MHz)
from 2-bromo-5-methylbenzoic acid
190 180 170 160 150 140 130 120 110 100 90 80 70 60 50 40 30 20 10 0
f1 (ppm)

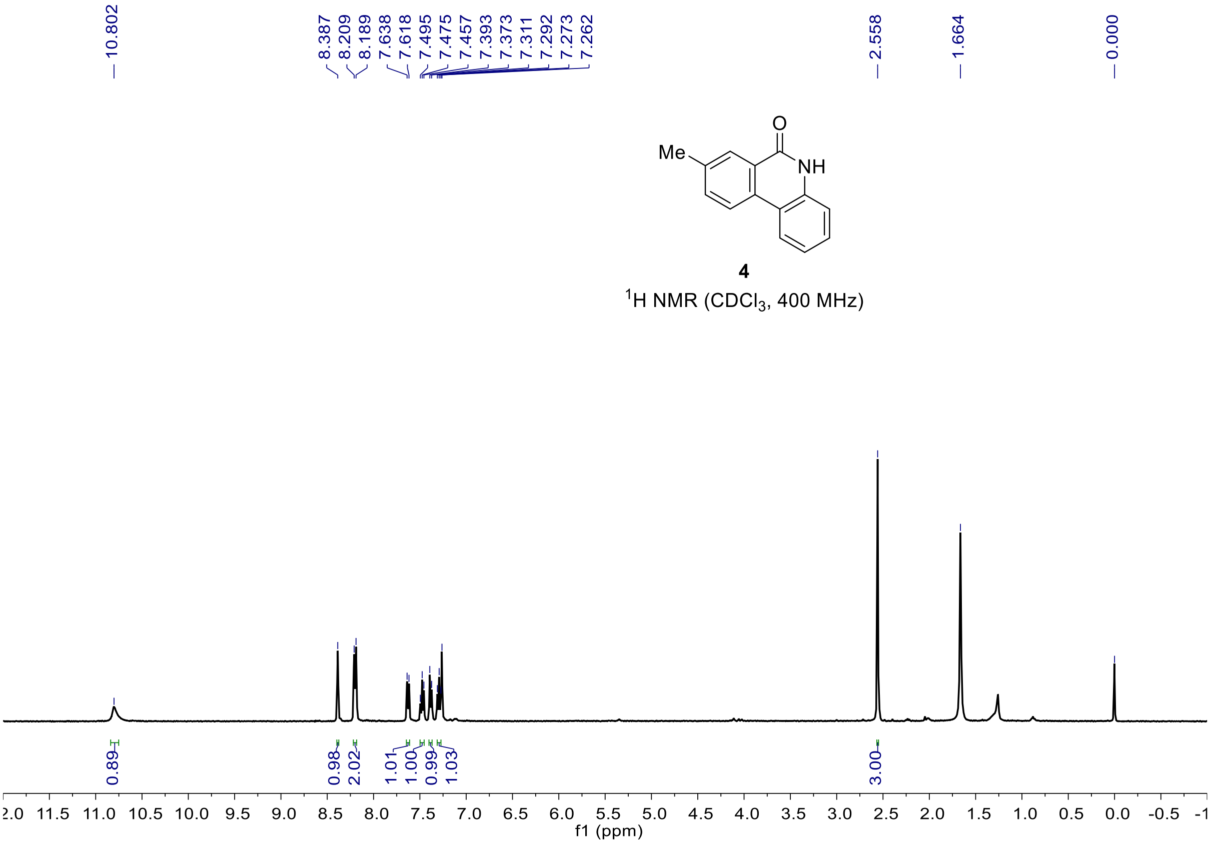
10.802
8.387
8.209
8.189
7.638
7.618
7.495
7.475
7.457
7.393
7.373
7.311
7.292
7.273
7.262
2.558
1.664
0.000
Me
O
NH
4
1H NMR (CDCl3, 400 MHz)
0.89
0.98
2.02
1.01
1.00
0.99
1.03
3.00
f1 (ppm)

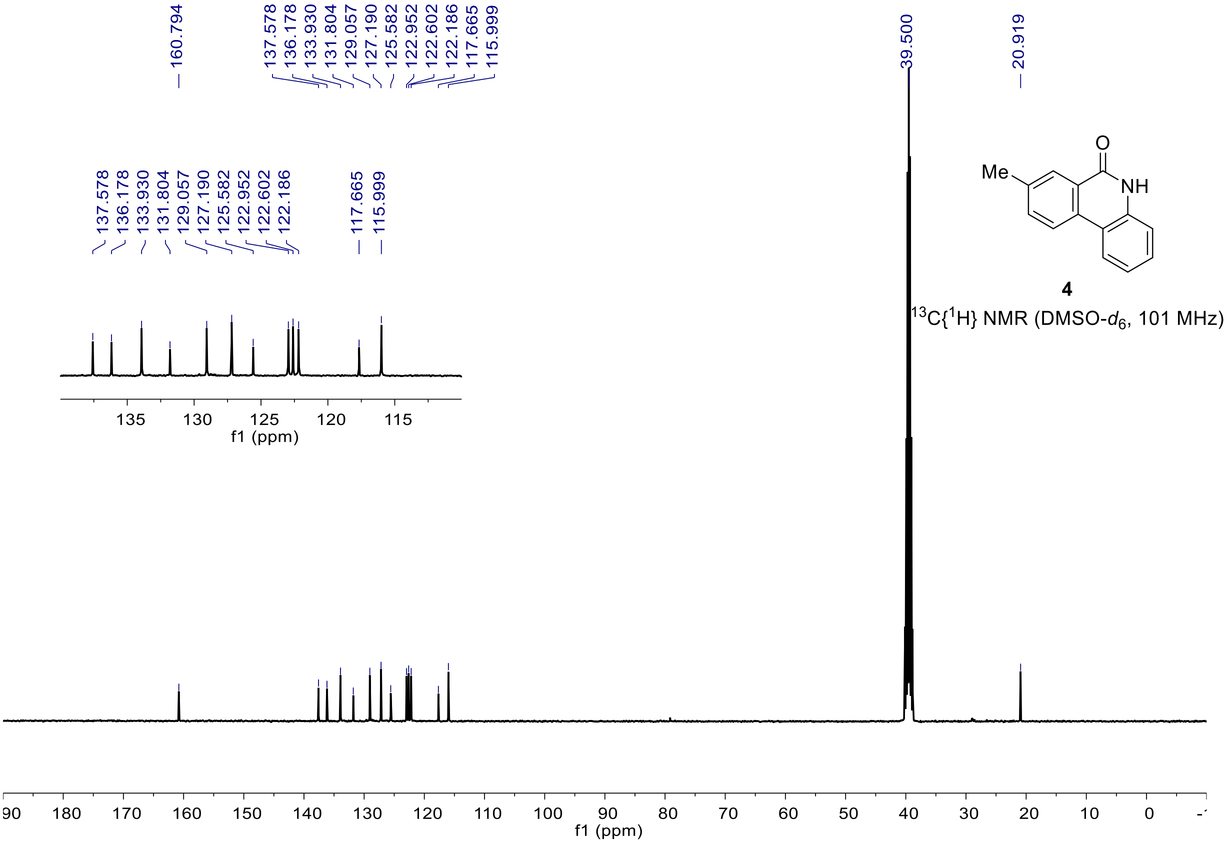
160.794
137.578
136.178
133.930
131.804
129.057
127.190
125.582
122.952
122.602
122.186
117.665
115.999
39.500
20.919
Me
O
NH
4
13C{1H} NMR (DMSO-d6, 101 MHz)
f1 (ppm)

V. References

1. Thorat, V. H.; Upadhyay, N. S.; Murakami, M.; Cheng, C.-H. Nickel-Catalyzed Denitrogenative Annulation of 1,2,3-Benzotriazin-4-(3*H*)-ones with Benzynes for Construction of Phenanthridinone Scaffolds. *Adv*. *Synth*. *Catal*. **2018**, *360*, 284-289.
2. Lu, C.; Dubrovskiy, A. V.; Larock, R. C. Palladium-Catalyzed Annulation of Arynes by *o*-Halobenzamides: Synthesis of Phenanthridinones. *J*. *Org*. *Chem*. **2012**, *77*, 8648-8656.
3. Verma, A.; Singh Banjara, L.; Meena, R.; Kumar, S. Transition-Metal-Free Synthesis of N-Substituted Phenanthridinones and Spiro-isoindolinones: C(sp^2)−N and C(sp^2)−O Coupling through Radical Pathway. *Asian*. *J*. *Org*. *Chem*. **2020**, *9*, 105-110.
4. Nageswar Rao, D.; Rasheed, S.; Das, P. Palladium/Silver Synergistic Catalysis in Direct Aerobic Carbonylation of C(sp^2)−H Bonds Using DMF as a Carbon Source: Synthesis of Pyrido-Fused Quinazolinones and Phenanthridinones. *Org*. *Lett*. **2016**, *18*, 3142-3145.
